# Management of hepatocellular carcinoma: an overview of major findings from meta-analyses

**DOI:** 10.18632/oncotarget.9157

**Published:** 2016-05-04

**Authors:** Xingshun Qi, Yan Zhao, Hongyu Li, Xiaozhong Guo, Guohong Han

**Affiliations:** ^1^ Department of Gastroenterology and Meta-analysis Study Interest Group, General Hospital of Shenyang Military Area, Shenyang, 110840 China; ^2^ Department of Liver Diseases and Digestive Interventional Radiology, Xijing Hospital of Digestive Diseases, Fourth Military Medical University, Xi'an, 710032 China; ^3^ Department of Gastroenterology, First Affiliated Hospital of the Medical College, Xi'an Jiaotong University, Xi'an, 710000 China

**Keywords:** hepatocellular carcinoma, treatment, management, meta-analysis, systematic review

## Abstract

This paper aims to systematically review the major findings from meta-analyses comparing different treatment options for hepatocellular carcinoma (HCC). A total of 153 relevant papers were searched via the PubMed, EMBASE, and Cochrane library databases. They were classified according to the mainstay treatment modalities (i.e., liver transplantation, surgical resection, radiofrequency ablation, transarterial embolization or chemoembolization, sorafenib, and others). The primary outcome data, such as overall survival, diseases-free survival or recurrence-free survival, progression-free survival, and safety, were summarized. The recommendations and uncertainties regarding the treatment of HCC were also proposed.

## INTRODUCTION

Hepatocellular carcinoma (HCC) is one of the most common causes of cancer-related death [1–3]. Currently, the most widely accepted therapeutic algorithm is derived from BCLC staging system [4–5], in which the mainstay treatment options for HCC include liver transplantation (LT), surgical resection, radiofrequency ablation (RFA), percutaneous ethanol injection (PEI), transarterial embolization (TAE) or chemoembolization (TACE), and sorafenib. Several novel therapeutic modalities have been also explored, such as percutaneous acetic acid injection (PAI), three-dimensional conformal radiation therapy (3D-CRT), argon-helium cryotherapy system (AHCS), traditional Chinese medicine (TCMs), cytokine-induced killer (CIK) cell therapy, and portal vein embolization (PVE), etc. It remains unclear about whether or such novel therapeutic modalities could be applied to the clinical practice. Meta-analysis can provide the highest level of evidence for our clinical decisions by combining all scattered data [6–7]. Herein, we systematically reviewed the major findings from all meta-analyses regarding the treatment of HCC and attempted to propose the evidence-based recommendations and uncertainties.

## RESULTS

**Figure 1:**
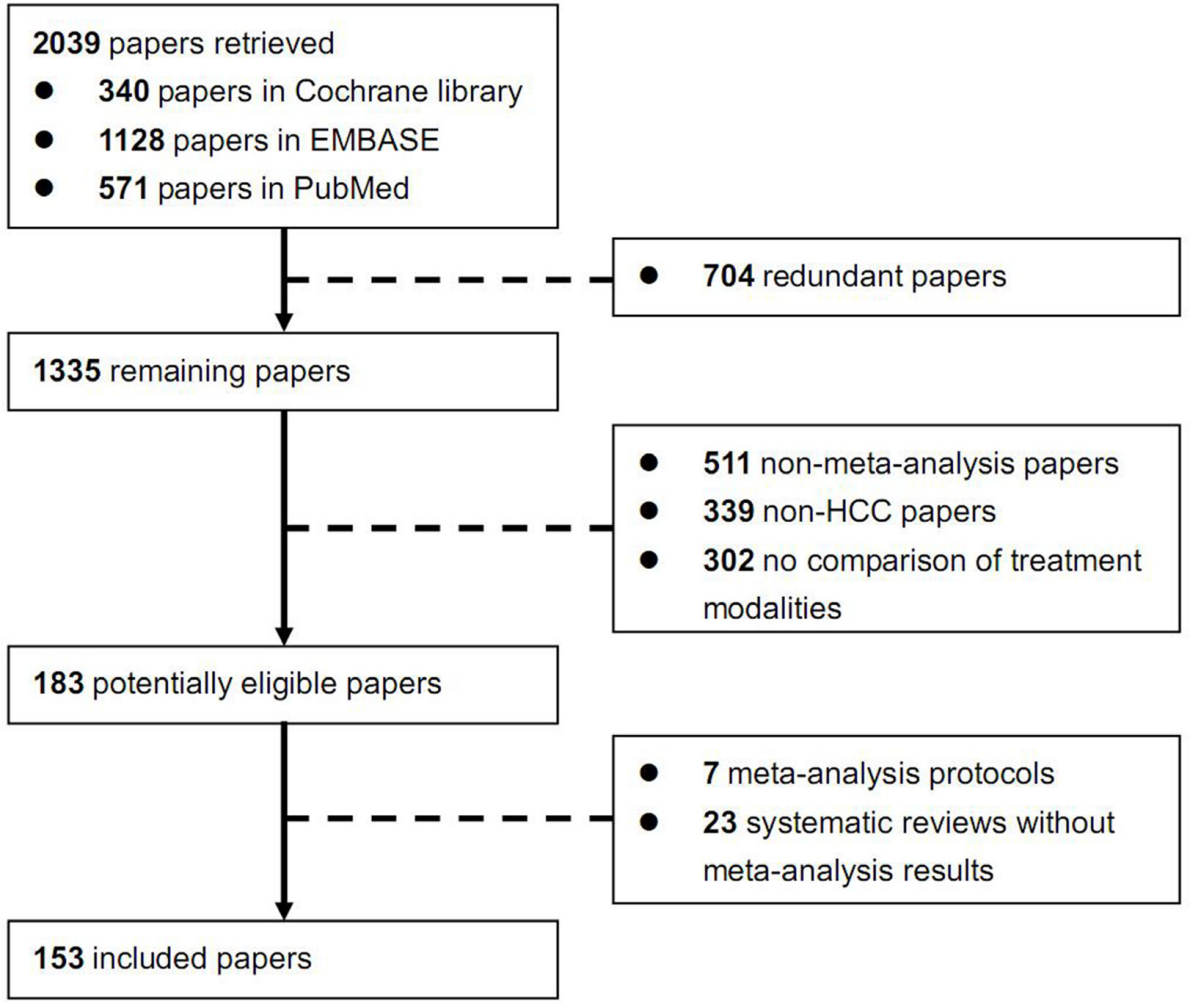
Flowchart of study inclusion

Overall, 2039 papers were identified. Among them, 153 meta-analysis papers were finally included [8–160] (Figure 1). The number of relevant papers was gradually increased over years (Supplementary Figure S1). The characteristics of these included papers were shown in Table 1. Their major findings were summarized according to the treatment modalities (Tables 2–5 and Supplementary Tables S1–S8).

**Table 1:** Study characteristics: An overview of included studies

First author	Journal (Year)	Country	Type of participants	No. included studies/pts.	Comparisons	Type of studies	
						RCT	Non-RCT
Al Hasan	Liver Transpl (2014)	Saudi Arabia	Unselected HCC	7/1388	Living donor LT vs deceased donor LT	0	7
Bouza	BMC Gastroenterol (2009)	Spain	Early, small HCC	6/787	RFA vs PEI	6	0
Breitenstein	Br J Surg (2009)	Switzerland	Unselected HCC	7/620	Interferon after resection or ablation	7	0
Cai	HPB (2013)	China	HCC ≤ 5 cm	5/NA	RFA vs surgical resection	NA	NA
Camma	Radiology (2002)	Italy	Unresectable HCC	18/2466	TACE vs non-active treatment; different transarterial modalities of therapy (TACE, TCE, TAE)	5; 13	0
Cao	Ultrasound Med Biol (2011)	China	Unselected HCC	9/736	TACE plus HIFU vs TACE alone	0	9
Chen	Dig Dis Sci (2011)	China	Unselected HCC	9/1503	Anatomic vs nonanatomic resection	0	9
Chen	Zhonghua Wai Ke Za Zhi (2008)	China	HCC ≤ 5 cm, number of lesions ≤ 3	6/697	RFA vs surgical resection	1	5
Chen	Chinese-German Journal of Clinical Oncology (2013)	China	Unresectable HCC	9/870	CIK cell + TACE	9	0
Cheng	J Cancer Res Clin Oncol (2014)	China	Resectable HCC	10/909	Preoperative TACE vs control; postoperative TACE vs control	4; 6	0; 0
Cheung	Evid Based Complement Alternat Med (2013)	China	Unresectable HCC	67/5211	TACE + Chinese medicines vs TACE alone	67	0
Cho	Hepatology (2009)	Korea	Unselected HCC	4/652	RFA vs PEI	4	0
Cho	Expert Opin Investig Drugs (2009)	China	HCC patients receiving TACE	30/2428	TACE + Chinese herbal therapy vs TACE alone	30	0
Chu	Asian J Surg (2010)	China	Post-operative HCC	5/206	Vitamin analogues (vitamin A and K2) after hepatic resection or local ablative therapy	5	0
Cinco	Hepatology International (2011)	Philippines	Advanced HCC	2/828	Sorafenib vs placebo	9	0
Cucchetti	Ann Surg Oncol (2012)	Italy	Unselected HCC	18/9036	Anatomic vs nonanatomic resection	0	18
Cucchetti	J Hepatol (2013)	Italy	Early HCC ≤ 5 cm	17/8420	RFA vs surgical resection	NA	NA
Dhir	HPB (2012)	USA	Early HCC within Milan Criteria	10/1763	LT vs resection	0	10
Dong	World J Gastroenterol (2014)	China	Unselected HCC	22/NA	Surgical resection vs nonsurgical-resection ablation therapies; RFA vs PEI; RFA vs RFA+TACE	2; 5; 0	10; 0; 5
Duan	World J Surg Oncol (2013)	China	Unselected HCC	12/8612	RFA vs surgical resection	2	12
Duffy	Hepatology (2013)	USA	Unselected HCC	6/2464	Antiangiogenic agents vs placebo	6	21
Estanislao	J Gastroenterol Hepatol (2009)	Philippine	Advanced HCC	NA/NA	Octreotide vs no octreotide	NA	NA
Fancellu	J Surg Res (2011)	Italy	Resectable HCC	9/590	Minimally-invasive vs open hepatectomy	0	9
Feng	J Cancer Res Clin Oncol (2014)	China	Small HCC (1 lesion < 6.5 cm; no more than 3 lesions < 4.5 cm)	23/15482	RFA vs surgical resection	3	20
Flores	J Gastroenterol Hepatol (2009)	Philippines	Post-operative HCC	2/236	Adjuvant immunotherapy in combination with surgical resection	2	0
Fu	Hepato-gastroenterology (2014)	China	Small HCC (single < 6.5 cm, or ≤ 3 lesions, ≤ 4.5 cm)	5/776	RFA vs surgical resection	5	0
Fu	J Cancer Res Clin Oncol (2014)	China	Unselected HCC	9/900	TACE + sorafenib vs TACE alone	5	4
Fu	J Cancer Res Ther (2014)	China	Unresectable HCC	9/608	Kanglaite injection plus hepatic arterial intervention vs hepatic arterial intervention alone	0	9
Furtado	Ann Surg Oncol (2014)	Australia	Unselected HCC	5/334	Surgery + Adjuvant I(131) lipiodol vs surgery alone	2	3
Gao	Hepato-gastroenterology (2013)	China	Unresectable HCC	7/693	DEB-TACE vs conventional TACE	0	7
Germani	J Hepatol (2010)	UK	Unselected HCC	8/1035	RFA vs PEI; Percutaneous acetic acid injection vs PEI	5; 2	0; 0
Geschwind	Am J Clin Oncol (2003)	USA	Unselected HCC	4/268	Therapeutic embolization vs supportive care alone	4	0
Gong	Nucl Med Commun (2014)	China	Unselected HCC	6/466	Adjuvant therapy with intra-arterial iodine-131-labeled lipiodol ((131)I-lipiodol) to hepatic resection	2	8
Grant	Clin Transplant (2013)	Canada	Unselected HCC	16/2202	Living donor LT vs deceased donor LT	0	16
Gu	J Cancer Res Clin Oncol (2014)	China	Unselected HCC	18/2120	TACE + local ablative therapy vs monotherapy	7	11
Guo	J Cancer Res Clin Oncol (2009)	China	Advanced HCC	6/352	Octreotide vs placebo or best supportive care	6	0
Han	Journal of Interventional Radiology (China) (2013)	China	Unselected HCC	8/698	RFA + TACE vs TACE	0	8
Han	PLoS One (2014)	China	Unresectable HCC	5/454	DEB-TACE vs conventional TACE	3	2
Hoshida	Hepatology (2000)	Japan	Non-advanced HCC	17/NA	Surgical resection vs PEI; Surgical resection vs LT	0; 0	5; 12
Hu	HPB (2013)	China	Unselected HCC	18/NA	RFA vs surgical resection	4	14
Huang	Hepato-gastroenterology (2013)	China	Unselected HCC	4/433	RFA vs cryosurgery ablation	0	4
Huang	J Gastroenterol Hepatol (2014)	China	Unresectable HCC	7/700	DEB-TACE vs conventional TACE	2	5
Huang	J Viral Hepat (2013)	China Taiwan	Unselected HCC	22/3156	Adjuvant interferon therapy after curative treatment	10	13
Huang	Zhonghua Nei Ke Za Zhi (2008)	China	Early HCC within Milan Criteria	6/862	RFA vs other therapeutic methods	6	0
Ji	Med Sci Monit (2011)	China	Advanced HCC	9/759	Octreotide vs placebo or no treatment	9	0
Jiang	Tumour Biol (2014)	China	Unselected HCC	19/1728	RFA + TACE vs RFA alone	8	11
Jiang	World J Surg Oncol (2013)	China	Unselected HCC	10/1029	Adjuvant interferon therapy after treatment with surgical resection or TACE	8	2
Kong	Tumour Biol (2014)	China	Unselected HCC	19/1728	RFA + TACE vs RFA alone	8	11
Lan	Journal of Gastroenterology and Hepatology Research (Hong Kong) (2013)	China	Unselected HCC	10/701	Lamivudine treatment vs no antiviral therapy after liver resection or RFA	0	10
Leng	ANZ J Surg (2014)	China	HCC patients with PVTT	5/600	TACE vs control treatment	2	3
Li	World J Gastroenterol (2012)	China	Unselected HCC	11/1013	Primary LT vs salvage LT	0	11
Li	Hepatol Res (2012)	China	Unselected HCC	10/627	Laparoscopic vs open liver resection	0	10
Li	Hepato-gastroenterology (2011)	China	Unselected HCC	4/776	Anatomic vs nonanatomic resection	0	4
Li	J Gastroenterol Hepatol (2012)	China	Unselected HCC	6/877	RFA vs surgical resection	2	4
Li	Chinese Journal of Evidence-Based Medicine (2012)	China	Intermediate-advanced stage	17/907	TACE + thermotherapy vs TACE alone	17	0
Li	Chinese Journal of Evidence-Based Medicine (2013)	China	Intermediate-advanced stage	16/1467	TACE + argon-helium cryotherapy system (AHCS) vs TACE alone; TACE + argon-helium cryotherapy system vs AHCS	15; 7	0
Li	Chinese Journal of Cancer Prevention and Treatment (2013)	China	Unselected HCC	8/818	Adjuvant IFN vs without IFN after curative treatment	8	0
Li	Clin Res Hepatol Gastroenterol (2014)	China	Unresectable HCC	11/936	CIK+TACE+RFA vs TACE+RFA; CIK+TACE vs TACE	6	5
Liang	Liver Transpl (2012)	China	Unselected HCC	7/1310	Living donor LT vs deceased donor LT	0	7
Liang	Liver Transpl (2012)	China	Unselected HCC	5/2950	Sirolimus-based immunosuppression (SRL) after LT vs SRL-free	0	5
Liao	PLoS One (2013)	China	Unselected HCC	28/2497	TACE+PEI vs TACE; TACE+RT vs TACE; TACE+ three-dimensional conformal radiation therapy (3D-CRT) vs TACE; TACE+RFA vs TACE; TACE+HIFU vs TACE	4; 3; 1; 1; 1	5; 4; 5; 0; 4
Liu	World J Gastroenterol (2010)	China	Unselected HCC	10/1522	RFA vs surgical resection	0	10
Liu	Surg Laparosc Endosc Percutan Tech (2010)	China	Unselected HCC	8/1188	RFA vs surgical resection	8	0
Liu	Tumour Biol (2014)	China	Unselected HCC	7/571	RFA + TACE vs RFA alone	7	0
Liu	PLoS One (2014)	China	Unresectable HCC	17/676	TACE + sorafenib vs monotherapy	3	14
Llovet	Hepatology (2003)	Spain	Unresectable HCC	14/1443	TACE vs control treatment; Tamoxifen vs control treatment	7; 7	0; 0
Lu	Eur J Gastroenterol Hepatol (2013)	China	Unselected HCC	7/574	RFA + TACE vs RFA alone	7	0
Ma	Experimental Hematology and Oncology (2012)	China	Unresectable HCC	13/1212	CIK cell therapy vs non-CIK therapy	13	0
Ma	Chinese Journal of Cancer Prevention and Treatment (2011)	China	Post-operative HCC	4/423	Hepatic resection or RFA alone vs surgery + adoptive immunotherapy	4	0
Marelli	Cardiovasc Intervent Radiol (2007)	UK	Unselected HCC	12/NA	TACE vs control treatment; TACE vs TAE alone	9; 3	0
Mathurin	Aliment Pharmacol Ther (2003)	France	Post-operative HCC	21/NA	Adjuvant therapy after curative liver resection	10	11
Meng	Radiother Oncol (2009)	China	Unselected HCC	17/1476	TACE + radiotherapy vs TACE alone	5	12
Meng	Hepatology International (2011)	China	Post-operative HCC	4/209	Vitamin K2 vs placebo after curative treatment	4	0
Meng	J Altern Complement Med (2008)	China	Unresectable HCC	37/2653	TACE + Traditional Chinese Medicine vs TACE alone	NA	NA
Meng	Explore (NY) (2011)	China	Unresectable HCC	12/1008	TACE + Traditional Chinese Medicine vs TACE alone	11	0
Menon	Aliment Pharmacol Ther (2013)	UK	Unselected HCC	5/474	Sirolimus-based immunosuppression (SRL) after LT vs SRL-free	0	5
Miao	World J Gastroenterol (2010)	China	Unselected HCC	16/1224	Adjuvant antiviral therapy after curative therapy	5	8
Miyake	J Viral Hepat (2010)	Japan	Unselected HCC	5/355	Interferon-alpha after curative therapy	3	2
Moriguchi	Hepatology (2006)	Japan	Unselected HCC	4/604	Tumor ablation plus interferon therapy	4	0
Ni	J Cancer Res Clin Oncol (2013)	China	Unselected HCC	10/21494	RFA/PEI (PAT) vs surgical resection	6	4
Ni	World J Gastroenterol (2013)	China	Unselected HCC	8/598	RFA + TACE vs RFA alone	8	0
Ni	J Cancer Res Clin Oncol (2013)	China	Unselected HCC	6/376	RFA + TACE vs RFA or TACE alone	6	0
Nowak	Cochrane Database Syst Rev (2004); Cancer (2005)	Australia	Unresectable HCC	10/1709	Tamoxifen vs placebo/no intervention	10	0
Oliveri	Cochrane Database Syst Rev (2011)	Denmark	Unresectable HCC	9/645	TACE or TAE vs placebo, sham, or no intervention	9	0
Ono	Cancer (2001)	Japan	Post-operative HCC	3/108	Adjuvant chemotherapy after resection	3	0
Orlando	Am J Gastroenterol (2009)	Italy	Small HCC	5/701	RFA vs PEI	5	0
Pang	Chinese Journal of Evidence-Based Medicine (2010)	China	Unselected HCC	7/309	Laparoscopic vs conventional open hepatectomy	0	7
Parks	HPB (Oxford) (2014)	USA	Unselected HCC	15/1002	Laparoscopic vs open liver resection	0	15
Proneth	Ann Surg Oncol (2014)	Germany	Unselected HCC	9/1572	LT vs resection	0	7
Qi	J Clin Gastroenterol (2014)	China	Early-stage HCC	3/559	RFA vs surgical resection	3	0
Rahman	J Gastrointest Surg (2012)	USA	Unselected HCC	9/2279	LT vs resection	0	9
Riaz	BMC Gastroenterol (2012)	Pakistan	Post-operative HCC	5/754	Vitamin K2 or its analogues vs placebo or No vitamin K	5	0
Schoppmeyer	Cochrane Database Syst Rev (2009)	Germany	Early HCC	3/261	PEI vs percutaneous acetic acid injection; PEI vs surgery	2; 1	0; 0
Shen	J Gastroenterol Hepatol (2013)	China	Small HCC < 3 cm	4/766	RFA vs PEI	4	0
Shen	J Clin Gastroenterol (2013)	China	Unresectable HCC	5/1462	Sorafenib vs placebo	5	0
Shen	J Hepatol (2010)	China Taiwan	Unselected HCC	13/1180	Adjuvant interferon therapy after curative therapy	9	4
Shu	Integr Cancer Ther (2005)	USA	Unresectable HCC	26/2079	Chinese herbal medicine + chemotherapy vs chemotherapy alone	24	2
Singal	Aliment Pharmacol Ther (2010)	USA	Unselected HCC	10/645	Interferon after resection or ablation	5	5
Sun	World Chinese Journal of Digestology (2011)	China	Small HCC	11/2965	RFA vs surgical resection	2	9
Sun	PLoS One (2014)	China	Unselected HCC	13/6350	Nucleot(s)ide analogues vs placebo or no treatment after curative treatment	1	12
Sun	Afr J Tradit Complement Altern Med (2012)	China	Unresectable HCC	10/726	TACE + Compound Kushen Injection vs TACE alone	0	10
Tang	Hepato-gastroenterology (2012)	China	Unselected HCC	5/799	Hepatectomy with a margin aiming at 2 cm vs a margin aiming at 1 cm	1	4
Tang	Hepato-gastroenterology (2013)	China	Resectable HCC	12/1829	Anatomic vs nonanatomic resection	0	12
Twaij	World J Gastroenterol (2014)	United Kingdom	Unselected HCC	4/420	Laparoscopic vs open liver resection	0	4
Wang	Hepato-gastroenterology (2011)	China	Unselected HCC	3/257	Preoperative TACE vs control	3	0
Wang	PLoS One (2014)	China	Early HCC	28/11873	RFA vs surgical resection	3	25
Wang N	Med Oncol (2011)	China	Unselected HCC	7/623	TACE + PEI vs TACE alone	7	0
Wang W	Liver Int (2010)	China	Unselected HCC	10/595	TACE + percutaneous ablation therapy (RFA or PEI) vs TACE or percutaneous ablation therapy alone	10	0
Wang	Asian Pac J Cancer Prev (2013)	China	Unselected HCC	4/1382	Sorafenib with or without chemotherapy vs placebo with or without chemotherapy	4	0
Wang	Can J Gastroenterol (2012)	China	Unresectable HCC	4/210	Prophylactic antibiotic treatment vs no prophylactic antibiotic treatment after transarterial therapy	3	1
Wang	Can J Gastroenterol (2013)	China	Post-operative HCC	27/2614	Different adjuvant therapy after potentially curative treatment	27	0
Weis	Cochrane Database Syst Rev (2013)	Germany	Unselected HCC	11/NA	RFA vs surgical resection; RFA vs PEI or RFA vs acetic acid injection; RFA vs microwave ablation; RFA vs laser ablation	3; 6; 1; 1	0; 0; 0; 0
Wong	Aliment Pharmacol Ther (2011)	China	Unselected HCC	9/551	Antiviral treatment vs no anti-viral treatment	0	9
Wu	J Exp Clin Cancer Res (2009)	China	Unresectable HCC	45/3236	Traditional Chinese Medicines vs other treatment	45	0
Wu	J Cancer Res Ther (2014)	China	Unresectable HCC	9/659	TACE + Cinobufacini vs TACE only	0	9
Xie	Tumour Biol (2014)	China	Advanced HCC	5/582	TACE vs TAE	5	0
Xie	J Cancer Res Clin Oncol (2012)	China	Unresectable HCC	13/1840	TACE vs microsphere embolization	7	6
Xie	PLoS One (2012)	China	Post-operative HCC	6/494	Adoptive immunotherapy vs non-immunotherapy after surgery	6	0
Xiong	World J Gastroenterol (2012)	China	Unselected HCC	9/550	Laparoscopic vs open liver resection	0	15
Xu	Journal of Xi'an Jiaotong University (Medical Sciences) (2012)	China	Unselected HCC	9/2145	LT vs resection	0	9
Xu	Hepatobiliary Pancreat Dis Int (2014)	China	Unselected HCC	17/4238	LT vs resection	0	17
Xu	World J Surg Oncol (2012)	China	Early HCC	13/2535	RFA vs surgical resection	2	11
Xu	Eur J Med Res (2014)	China	Small HCC < 5 cm	6/983	RFA vs PEI	6	0
Xu	Hepatol Res (2014)	China	Unselected HCC	9/1565	Adjuvant interferon therapy after surgical treatment	5	4
Xue	BMC Gastroenterol (2013)	China	Advanced HCC with PVTT	8/1601	TACE vs conservative treatment	0	8
Yan	Dig Dis Sci (2012); Dig Dis Sci (2013)	China	Unselected HCC	19/1728	RFA + TACE vs RFA alone	8	11
Yang	Mol Biol Rep (2014)	China	Unresectable HCC	6/1181	TACE + sorafenib vs TACE alone	3	3
Yao	Chinese Journal of Evidence-Based Medicine (2013)	China	Unselected HCC	13/701	Laparoscopic vs open hepatectomy	0	13
Ye	Asian Pac J Cancer Prev (2012)	China	Unselected HCC	11/1576	Anatomic vs nonanatomic resection	0	11
Yin	Ann Surg Oncol (2013)	China	Unselected HCC	15/1238	Laparoscopic vs open hepatectomy	0	15
Yu	Chinese-German Journal of Clinical Oncology (2013)	China	Unselected HCC	7/1347	Preoperative TACE vs control	0	7
Zhang	PLoS One (2014)	China	Unresectable/advanced HCC	6/1254	TACE + sorafenib vs TACE alone	2	4
Zhang	Anticancer Drugs (2010)	China	Advanced HCC	3/924	Sorafenib-based therapy with other agent-based therapy	3	0
Zhang	Hepatobiliary Pancreat Dis Int (2012)	China	Advanced HCC	6/1164	Sorafenib vs placebo	3	3
Zhang	Molecular and Clinical Oncology (2014)	China	Unselected HCC	14/1385	Adjuvant interferon after curative surgery or ablation therapy	9	5
Zhang	Int J Cancer (2009)	China	Post-surgical or ablative HCC	6/600	IFN-alpha with placebo or no treatment after tumor resection or ablation	6	0
Zhao	Journal of Interventional Radiology (China) (2013)	China	Unselected HCC	21/2339	RFA + TACE vs RFA or TACE alone	21	0
Zhao	Zhonghua Wai Ke Za Zhi (2008)	China	Unselected HCC	9/494	Preoperative portal vein embolization (PVE) vs no PVE for extended hepatectomy	0	9
Zheng	Transplantation (2014)	China	Unselected HCC	62/10170	LT vs resection	0	62
Zheng	Int J Cancer (2014)	China	Unselected HCC	48/4747	Hepatectomy + adjuvant chemotherapy vs hepatectomy alone	13	35
Zhong	Hepatol Res (2010)	China	UICC TNM stage 3A HCC	6/659	Postoperative adjuvant TACE	6	0
Zhong	World Chinese Journal of Digestology (2012); PLoS One (2013)	China	Post-operative HCC	6/930	Curative treatments alone vs curative treatments + oral vitamin K2 analogs	6	0
Zhong	Molecular and Clinical Oncology (2014)	China	Post-operative HCC	3/286	Adjuvant conventional oral systemic chemotherapy after curative hepatic resection	3	0
Zhou	Dig Dis Sci (2011)	China	Unselected HCC	10/494	Laparoscopic vs open resection	0	10
Zhou	Langenbecks Arch Surg (2011)	China	Unselected HCC	16/2917	Anatomic vs nonanatomic resection	0	16
Zhou	BMC Gastroenterol (2013)	China	Unselected HCC	21/3210	Preoperative TACE vs no preoperative TACE	4	17
Zhou	Zhonghua Wai Ke Za Zhi (2011)	China	Small HCC (Milan criteria)	4/539	RFA vs surgical resection	4	0
Zhou	BMC Gastroenterol (2010)	China	Small HCC (Yao's criteria)	10/1411	RFA vs surgical resection	1	9
Zhou	World J Surg (2014)	China	Unselected HCC	20/8204	Antiviral therapy after curative resection	1	19
Zhu	Transplant Proc (2013)	China	Unselected HCC	14/1508	Primary LT vs salvage LT	0	14
Zhuang	PLoS One (2013)	China	Unselected HCC	13/1344	Interferon after curative therapy	13	0
Zhuang	Zhonghua Gan Zang Bing Za Zhi (2012)	China	Unselected HCC	8/857	Interferon after curative therapy	8	0
Zou	Acta Academiae Medicinae Sinicae (2011)	China	Advanced HCC	2/828	Sorafenib vs placebo	2	0

**Table 2:** Findings of meta-analyses: An overview of included studies regarding LT

First author	Journal (Year)	Comparisons	OS	DFS, RFS, TTP, PFS	Recurrence, time to recurrence	Other endpoints	Major comments
**Living donor LT (LDLT) versus deceased donor LT (DDLT)**							
Al Hasan	Liver Transpl (2014)	LDLT vs DDLT	OS: 1-, 3-, 5-year: statistically similar.	DFS: 1-, 3-, 5-year: statistically similar.	Recurrence: 1-, 3-year: statistically similar. 5-year: favor DDLT.	NA.	OS and DFS are comparable, but long-term recurrence is higher in LDLT.
Grant	Clin Transplant (2013)	LDLT vs DDLT	OS: statistically similar.	DFS: favor DDLT.	NA.	NA.	LDLT has lower DFS than DDLT.
Liang	Liver Transpl (2012)	LDLT vs DDLT	OS: 1-, 3-, 5-year: statistically similar.	RFS: 1-, 3-, 5-year: statistically similar.	Recurrence: statistically similar.	NA.	LDLT is an acceptable option especially for patients within Milan criteria.
**Primary LT versus salvage LT**							
Li	World J Gastroenterol (2012)	Primary LT vs salvage LT	OS: 1-, 3-, 5-year: statistically similar.	DFS: 1-, 3-, 5-year: statistically similar.	NA.	NA.	OS and DFS were not significantly different.
Zhu	Transplant Proc (2013)	Primary LT vs salvage LT	OS: 1-, 3-, 5-year: statistically similar.	DFS: 1-, 3-year: statistically similar. 5-year: favor primary LT.	NA.	Operative time: longer in salvage LT. Intraoperative blood loss: increased in salvage LT. Number of transfused units of packed red blood cells: larger in salvage LT. Length of hospital stay and ICU stay: statistically similar. Peri-operative mortality: statistically similar.	Salvage LT achieves the same short- and long-term outcomes as primary LT.
**Sirolimus-based immunosuppression (SRL) after LT**							
Liang	Liver Transpl (2012)	SRL after LT vs SRL-free	OS: 1-, 3-, 5-year: favor SRL.	DFS: 1-year: favor SRL.	Recurrence: 1-year: favor SRL.	Major SRL-related post-transplant complications: statistically similar.	SRL is safe and prolongs survival and decrease tumor recurrence.
Menon	Aliment Pharmacol Ther (2013)	SRL after LT vs SRL-free	Overall mortality: favor SRL.	RFS: better in SRL (no statistical comparison). Recurrence-related mortality: low in SRL.	Recurrence: favor SRL.	NA.	SRL has lower recurrence rate, lower overall mortality and longer RFS and OS.
**LT versus surgical resection**							
Dhir	HPB (2012)	LT vs resection	OS: 5-year: early HCC: favor LT. early HCC with well compensated cirrhosis: favor LT. early HCC using ITT strategy: statistically similar. early HCC with well compensated cirrhosis using ITT strategy: favor LT.	NA.	NA.	NA.	LT has favorable survival advantage in some settings.
Hoshida	Hepatology (2000)	Surgical resection vs PEI and LT (including LT vs surgical resection)	OS: Surgical resection vs LT: 3-year: statistically similar.	DFS: Surgical resection vs LT: 3-year: favor LT.	NA.	NA.	LT improved 3-year DFS for HCC patients.
Proneth	Ann Surg Oncol (2014)	LT vs resection	OS: 5-year: statistically similar.	NA.	NA.	NA.	LR is a good alternative to LT in patients with resectable HCC in whom both seem feasible.
Rahman	J Gastrointest Surg (2012)	LT vs resection	OS: 1-year: all studies: favor resection. non-ITT analysis: statistically similar. ITT analysis: favor resection. 5-year: all studies: statistically similar. non-ITT analysis: statistically similar. ITT analysis: favor LT. 10-year: all studies: favor LT. non-ITT analysis: favor LT. ITT analysis: favor LT.	DFS: 1-year: all studies: statistically similar. non-ITT analysis: statistically similar. ITT analysis: statistically similar. 5-year survival: all studies: favor LT. non-ITT analysis: favor LT. ITT analysis: favor LT. 10-year: all studies: favor LT. non-ITT analysis: favor LT. ITT analysis: statistically similar.	NA.	NA.	LT results in increased DFS and OS.
Xu	Journal of Xi'an Jiaotong University (2012)	LT vs resection	OS: 1-year: statistically similar. 3-, 5-year survival: favor LT.	DFS: 5-year: favor LT.	Recurrence: favor LT.	NA.	Both treatments were effective. But LT has a better prognosis than resection.
Xu	Hepatobiliary Pancreat Dis Int (2014)	LT vs resection	OS: 1-, 3-year: statistically similar. 5-year survival: favor LT.	DFS: 1-, 3-, 5-year: favor LT.	NA.	Surgery-related morbidity: higher in LT. Surgery-related mortality: higher in LT.	Long-term survival and tumor-free survival are higher in LT than in resection.
Zheng	Transplantation (2014)	LT vs resection	OS: 1-year: statistically similar. 3-, 5-year: favor LT.	DFS: 1-, 3-, 5-year: favor LT.	Recurrence: favor LT.	NA.	LT provides increased survival and lower recurrence rates than resection.

**Table 3:** Findings of meta-analyses: An overview of included studies regarding surgical resection

First author	Journal (Year)	Comparisons	OS	DFS, RFS, TTP, PFS	Recurrence, time to recurrence	Other endpoints	Major comments
**Surgical resection margin**							
Tang	Hepato-gastroenterology (2012)	Hepatectomy with a margin aiming at 2 cm vs a margin aiming at 1 cm	OS:RCT:1-year: statistically similar.3-, 5-year: favor hepatectomy with a margin aiming at 2 cm.Non-RCT:1-, 3-, 5-year: statistically similar.	DFS:RCT: favor hepatectomy with a margin aiming at 2 cm.Non-RCT: statistically similar.	NA.	Post-operative morbidity: statistically similar.Post-operative mortality: statistically similar.Blood loss: statistically similar.	Survival was similar between resection aiming at 2 cm and 1 cm.
**Laparoscopic resection (LR) versus open liver resection (OR)**							
Fancellu	J Surg Res (2011)	Minimally-invasive vs open hepatectomy	OS:1-, 2-, 3-, 5-year: statistically similar.	DFS:1-, 2-, 3-, 5-year: statistically similar.	NA.	Operative time: statistically similar. Blood loss: less in minimally-invasive hepatectomy. Transfusion: lower in minimally-invasive hepatectomy.Peri-operative complications: lower in minimally-invasive hepatectomy. Postoperative stay: shorter in minimally-invasive hepatectomy. Positive margin: lower in minimally-invasive hepatectomy.	Minimally-invasive hepatectomy was associated with adverse events after procedure.
Li	Hepatol Res (2012)	LR vs OR	NA.	NA.	Tumor recurrence: statistically similar.	Operative time: statistically similar.Blood loss: less in LR.Need for blood transfusion: fewer in LR.Postoperative complications: fewer in LR. Hospital stay: shorter in LR.	LR is a safe and feasible choice for selected HCC.
Pang	Chinese Journal of Evidence-Based Medicine (2010)	LR vs OR	In-hospital mortality: statistically similar.	NA.	NA.	Operation time: longer in LR. Blood loss: less in LR. Blood transfusion: statistically similar.Postoperative complications: less in LR. Length of stay: shorter in LR.	LR is associated with less complications.
Parks	HPB (Oxford) (2014)	LR vs OR	OS:1-, 3-, 5-year: statistically similar.	NA.	NA.	Operative time: statistically similar. Blood loss: lower in LR.	LR should be an acceptable alternative treatment.
Twaij	World J Gastroenterol (2014)	LR vs OR	NA.	NA.	NA.	Operative time: statistically similar. Blood loss: less in LR.Transfusion: greater in OR.Post-operative morbidity: less in LR.In-hospital length: shorter in LR. Tumor margins: larger in LR.	LR is safe and may provide improved patient outcomes when compared to the open technique.
Xiong	World J Gastroenterol (2012)	LR vs OR	NA.	NA.	Tumor recurrence: statistically similar.	Mean operation time: statistically similar.Blood loss: less in LR.Blood transfusions requirement: less in LR.Postoperative complications: statistically similar. Liver failure: less in LR.Ascites: less in LR.Pulmonary complications: statistically similar.Length of hospital stay: shorter in LR. Positive margins: statistically similar.	LR appears to be a safe and feasible option for resection of HCC in selected patients.
Yao	Chinese Journal of Evidence-Based Medicine (2013)	LR vs OR	OS:3-, 5-year: statistically similar.Peri-operative mortality: statistically similar.	Tumor-free survival:3-, 5-year: statistically similar.	NA.	Operative time: statistically similar. Intraoperative bleeding: less in LR. Postoperative complications: lower in LR. Hospital stay: shorter in LR.	LR is safe and feasible for treating HCC.
Yin	Ann Surg Oncol (2013)	LR vs OR	OS:1-, 3-, 5-year: statistically similar.	RFS:1-, 3-, 5-year: statistically similar.	NA.	Operative time: statistically similar. Blood loss: less in LR.Blood transfusion: less in LR.Hospital stay: shorter in LR. Surgery margin: statistically similar.	LR may have short-term advantages in terms of blood loss and postoperative morbidity for HCC. Both procedures have similar long-term outcomes.
Zhou	Dig Dis Sci (2011)	LR vs OR	OS: statistically similar.	DFS: statistically similar.	NA	Operative time: statistically similar.Blood loss: less in LR.Blood transfusion: less in LR.Cirrhotic decompensation/ascites: lower in LR.Liver failure: lower in LR.Bile leakage and bleeding: statistically similar.Pulmonary complications: less in LR. Hospital stay: shorter in LR.	LR may be an alternative choice for treatment of HCC.
**Anatomic resection (AR) versus nonanatomic resection (NAR)**							
Chen	Dig Dis Sci (2011)	AR vs NAR	OS:5-year: statistically similar.	DFS:5-year: favor AR.	Local recurrence:statistically similar.	NA.	AR is associated with better DFS than NAR.
Cucchetti	Ann Surg Oncol (2012)	AR vs NAR	OS: 5-year: favor AR.	DFS: 5-year: favor AR.	NA.	Postoperative morbidity: statistically similar.	Patient survival and DFS after AR seem to be superior to NAR.
Li	Hepato-Gastroenterology (2011)	AR vs NAR	OS:1-, 3-, 5-year: statistically similar.	DFS:1-, 3-, 5-year: statistically similar.	NA.	NA.	AR can extend 3-year DFS of patients with small HCC in the sensitivity analysis.
Tang	Hepato-gastroenterology (2013)	AR vs NAR	OS:1-, 3-, 5-year: statistically similar.	NA.	Recurrence: statistically similar.	Postoperative complications and blood loss: statistically similar.	AR does not provide significant benefit in the survival, recurrence and morbidity.
Ye	Asian Pac J Cancer Prev (2012)	AR vs NAR	OS: statistically similar.	DFS: favor AR.	Local intrahepatic recurrence:lower in AR.Overall intrahepatic recurrence: lower in AR.Early intrahepatic recurrence: lower in AR.Late intrahepatic recurrence: statistically similar.	Safety:Postoperative mortality: statistically similar.Postoperative morbidity: statistically similar.	AR was superior to NAR in terms of local recurrence and 5-year DFS.
Zhou	Langenbecks Arch Surg (2011)	AR vs NAR	OS:3-year: statistically similar.5-year: favor AR.	DFS:3-, 5-year: favor AR.	Local intrahepatic recurrence: lower in AR.Early recurrence:lower in AR.	Postoperative morbidity (liver failure, bile leakage, intra-abdominal bleeding, ascites, intra-abdominal abscess, upper gastrointestinal bleeding, pulmonary problem, pleural effusion, and wound problem): statistically similar.Postoperative mortality: statistically similar.Length of hospital stay: statistically similar.	AR was superior to NAR in terms of better survival and preventing local recurrence.
**Surgical resection + I**^131^**lipiodol versus resection**							
Furtado	Ann Surg Oncol (2014)	Surgery + adjuvant I^131^ lipiodol vs surgery alone	OS:1-, 2-, 3-, 5-year: favor surgery + adjuvant I^131^ lipiodol.	DFS:1-, 2-, 3-, 5-year: favor surgery + adjuvant I^131^ lipiodol.	NA.	NA.	There is strong evidence for the use of adjuvant I^131^ lipiodol to prolong DFS and OS, up to 5 years after resection.
Gong	Nucl Med Commun (2014)	Adjuvant therapy with I^131^ lipiodol to hepatic resection	OS:3-, 5-year: favor surgery + adjuvant I^131^ lipiodol.	NA.	Recurrence: 2-, 5-year: lower in surgery + adjuvant I^131^ lipiodol.	NA.	Postoperative adjuvant therapy with intra-arterial I^131^ lipiodol to hepatic resection of HCC significantly improves OS and DFS rates and reduces recurrence rates.
**Surgical resection + TACE versus resection**							
Cheng	J Cancer Res Clin Oncol (2014)	Preoperative and postoperative TACE vs control	Preoperative TACE - OS:statistically similar.Mean tumor size ≥ 5 cm: statistically similar.Mean tumor size < 5 cm: statistically similar.Postoperative TACE - OS:favor postoperative TACE.Mean tumor size ≥ 5 cm: favor postoperative TACE.Mean tumor size < 5 cm: NA.	Preoperative TACE - DFS:statistically similar.Mean tumor size ≥ 5 cm: statistically similar.Mean tumor size < 5 cm: statistically similar.Postoperative TACE - DFS:favor postoperative TACE.Mean tumor size ≥ 5 cm: favor postoperative TACE.Mean tumor size < 5 cm: statistically similar.	NA.	NA.	Preoperative TACE did not improve DFS and OS for curative resection of HCC.Postoperative TACE offers potential benefits for curative HCC (tumor size > 5 cm).
Wang	Hepato-gastroenterology (2011)	Preoperative TACE vs control	OS:statistically similar.	DFS:statistically similar.	NA.	NA.	There are no significant benefits for 5-year OS and DFS.
Yu	Chinese-German J Clinical Oncology (2013)	Preoperative TACE vs control	OS:5-year: favor preoperative TACE.	DFS:3-year: statistically similar.5-year: favor preoperative TACE.	NA.	NA.	Preoperative TACE can improve the 5-year DFS and OS rate.
Zhong	Hepatol Res (2010)	Postoperative adjuvant TACE	Mortality:1-, 3-year: favor postoperative TACE. 5-year: statistically similar.	NA.	Tumor recurrence: less in postoperative TACE.	NA.	Postoperative adjuvant TACE seems promising for HCC with risk factors (multiple nodules of > 5 cm or vascular invasion).
Zhou	BMC Gastroenterol (2013)	Preoperative TACE vs no preoperative TACE	OS:5-year: statistically similar.	DFS:5-year: statistically similar.	Total recurrence: statistically similar.Intrahepatic recurrence:statistically similar.Extrahepatic recurrence:statistically similar.	Overall morbidity and in-hospital mortality: statistically similar.	Preoperative TACE does not seem to improve prognosis for resectable HCC.
**Surgical resection + adjuvant chemotherapy versus resection**							
Mathurin	Aliment Pharmacol Ther (2003)	Adjuvant therapy + curative liver resection	OS:Pre-operative transarterial chemotherapy:RCTs:1-, 2-, 3-year: statistically similar.Both RCTs and Non-RCTs:1-, 2-, 3-year: statistically similar.Post-operative transarterial chemotherapy:RCTs:1-year: statistically similar.2-, 3-year: favor post-operative transarterial chemotherapy.Both RCTs and Non-RCTs:1-, 2-, 3-year: favor post-operative transarterial chemotherapy.Oral 5-fluorouracil:Both RCTs and Non-RCTs:1-, 2-, 3-year: statistically similar.Combination of systemic and transarterial chemotherapy:1-, 2-, 3-year: statistically similar.	NA.	Cumulative probability of no recurrence:Pre-operative transarterial chemotherapy:RCTs:1-, 2-, 3-year: statistically similar.Both RCTs and Non-RCTs:2-year: favor pre-operative transarterial chemotherapy.1-, 3-year: statistically similar.Post-operative transarterial chemotherapy:RCTs:1-, 2-, 3-year: favor post-operative transarterial chemotherapy.Both RCTs and Non-RCTs:1-, 2-, 3-year: favor post-operative transarterial chemotherapy.Oral 5-fluorouracil:Both RCTs and Non-RCTs:1-, 3-year: statistically similar.Combination of systemic and transarterial chemotherapy:1-, 2-, 3-year: statistically similar.	NA.	Post-operative transarterial chemotherapy improved survival and decreased the cumulative probability of no recurrence.
Ono	Cancer (2001)	Adjuvant chemotherapy after resection	OS:worse in adjuvant chemotherapy after resection.	DFS:statistically similar.	NA.	NA.	Cancer recurrence in the remnant liver is enhanced and the long-term outcome is deteriorated by postoperative chemotherapy after resection of HCC in cirrhotic patients.
Wang	Can J Gastroenterol (2013)	Different adjuvant therapy after potentially curative treatment (including chemotherapy)	OS:Chemotherapy: statistically similar.	RFS:Chemotherapy: statistically similar.	NA.	Side effects: Only systematic reviews, but not meta-analyses.	Combination of systemic and transhepatic arterial chemotherapy is not recommended for HCC after potentially curative treatment.
Zheng	Int J Cancer (2014)	Hepatectomy + adjuvant chemotherapy vs hepatectomy alone	OS:1-, 2-, 3-year: favor hepatectomy + adjuvant chemotherapy.	DFS:1-, 2-, 3-year: favor hepatectomy + adjuvant chemotherapy.	NA.	NA.	Adjuvant chemotherapy is an effective treatment to improve the prognosis of primary HCC patients who underwent hepatectomy.
Zhong	Mol Clin Oncol (2014)	Adjuvant conventional oral systemic chemotherapy after curative hepatic resection	OS:1-, 3-, 5-year: statistically similar.	DFS:1-, 3-, 5-year: statistically similar.	NA.	Side effects: NA. (Only systematic reviews, but not meta-analyses.)	Adjuvant conventional oral systemic chemotherapy provides only marginal benefits for HCC patients undergoing curative hepatic resection.
**Surgical resection + immunotherapy versus resection**							
Flores	J Gastroenterol Hepatol (2009)	Adjuvant immunotherapy in combination with surgical resection	OS:statistically similar.	NA.	Recurrence: statistically similar.	NA.	Adjuvant immunotherapy only shows a trend towards a benefit in improving survival and decreasing risk of tumor recurrence among patients with HCC after hepatic resection.
Ma	Chinese J Cancer Prevention and Treatment (2011)	Hepatic resection or RFA alone vs surgery + adoptive immunotherapy	Mortality:1-, 3-year: statistically similar.	NA.	Recurrence:1-year: favor surgery + adoptive immunotherapy.3-year: statistically similar.	NA.	Adjuvant immunotherapy seems promising for patients with HCC after hepatic resection or radiofrequency ablation.
Wang	Can J Gastroenterol (2013)	Different adjuvant therapy after potentially curative treatment (including adoptive immunotherapy)	OS:Adoptive immunotherapy: statistically similar.	RFS:Adoptive immunotherapy: favor adoptive immunotherapy.	NA.	Side effects: Only systematic reviews, but not meta-analyses.	Adjuvant immunotherapy produce limited success for survival.
Xie	PLoS One (2012)	Adoptive immunotherapy vs non-immunotherapy after surgery	OS:3-year: statistically similar.	NA.	Recurrence:1-, 3-year: favor adoptive immunotherapy.	NA.	Adjuvant immunotherapy with cytokine induced killer cells or lymphokine activated killer cells may reduce recurrence in postoperative HCC, but may not improve survival.
**Surgical resection + portal vein embolization (PVE) versus resection**							
Zhao	Zhonghua Wai Ke Za Zhi (2008)	PVE vs no PVE for extended hepatectomy	OS:1-, 3-, 5-year: statistically similar.	NA.	Intrahepatic and distant recurrence:statistically similar.	Liver failure after resection:favor PVE.Post-operative mortality:statistically similar.	PVE is a safe and effective procedure to prevent postresection liver failure due to insufficient liver remnant.

**Table 4:** Findings of meta-analyses: An overview of included studies regarding ablation therapy

First author	Journal (Year)	Comparisons	OS	DFS, RFS, TTP, PFS	Recurrence, time to recurrence	Other endpoints	Major comments
**RFA versus resection**							
Cai	HPB (2013)	RFA vs surgical resection	OS:1-, 3-, 5-year: statistically similar.	DFS:1-, 3-, 5-year: favor resection.	Local recurrence: favor resection.	NA.	For solitary HCC ≤ 5 cm, RFA can achieve comparable OS as resection, but higher recurrence rate and lower DFS.
Chen	Zhonghua Wai Ke Za Zhi (2008)	RFA vs surgical resection	OS:1-, 3-, 4-year: statistically similar.	Tumor-free survival:1-year: statistically similar.3-year: favor resection.	NA.	NA.	RFA is similar to resection on small HCC, RFA is the first-line treatment choice.
Cucchetti	J Hepatol (2013)	RFA vs surgical resection	OS:A single nodule < 2 cm: statistically similar.A single nodule ≤ 3 cm: favor resection.A single nodule 3–5 cm: favor resection.2–3 nodules < 3 cm: statistically similar.	DFS:A single nodule < 2 cm: statistically similar.A single nodule ≤ 3 cm: favor resection.A single nodule 3–5 cm: favor resection.2–3 nodules < 3 cm: statistically similar.	Local recurrence:statistically similar.	Cost-effectiveness analysis is also performed.	For very early HCC with 2–3 nodules < 3 cm, RFA is more cost-effective than resection. For single larger early stage HCCs, surgical resection remains the best strategy.
Duan	World J Surg Oncol (2013)	RFA vs surgical resection	OS:1-, 3-, 5-year: favor resection.	DFS:1-, 3-, 5-year: favor resection.	NA.	Complications: less in RFA.Hospital stay: longer in resection.	The long-term efficacy of resection is better than that of RFA, but with more complications and a longer hospital stay.
Feng	J Cancer Res Clin Oncol (2014)	RFA vs surgical resection	OS:1-, 3-, 5-year: favor resection.	RFS:1-, 3-, 5-year: favor resection.	Recurrence:1-year: statistically similar.2-, 3-year: favor resection.	Complication: Morbidity: lower in RFA.Mortality: statistically similar.	Surgical resection leads to a higher OS and RFS rate in treating small HCC.
Fu	Hepato-gastroenterology (2014)	RFA vs surgical resection	OS:1-, 3-year: statistically similar.5-year: favor resection.	RFS:1-year: statistically similar.3-, 5-year: favor resection.	Recurrence:1-year: statistically similar.2-, 3-year: favor resection.	Complications: higher in resection.	Resection led to a higher long-term survival rate and a lower long-term recurrence rate.
Hu	HPB (2013)	RFA vs surgical resection	OS:3-, 5-year: favor resection.	NA.	Local recurrence:favor resection.	Complications: lower in RFA.	Resection has higher 3- and 5-year survival, lower recurrence, and more complications.
Li	J Gastroenterol Hepatol (2012)	RFA vs surgical resection	OS: 1-, 3-, 5-year: favor resection. HCC ≤ 3 cm: 1-, 5-year: statistically similar.3-year: favor resection.	RFS: favor resection.	Recurrence: favor resection.	Complications: statistically similar.	Resection was superior to RFA in the treatment of HCC.
Liu	World J Gastroenterol (2010)	RFA vs surgical resection	OS:1-year, 3-year, end of follow-up: statistically similar.	NA.	Recurrence:1-, 3-year: statistically similar.end of follow-up: favor RFA.	NA.	RFA did not decrease the number of overall recurrences, and had no effect on survival when compared with surgical resection in a selected group of patients.
Liu	Surg Laparosc Endosc Percutan Tech (2010)	RFA vs surgical resection	OS:1-, 2-year: statistically similar.3-, 5-year: favor resection.	RFS:1-, 3-, 5-year: favor resection.	Recurrence in previous sites: favor resection.Recurrence in new areas:favor RFA.Recurrence of extrahepatic areas:statistically similar.	NA.	RFA may have comparable results with surgical resection.
Ni	J Cancer Res Clin Oncol (2013)	RFA/PEI (PAT) vs surgical resection	OS:1-year: statistically similar.2-, 3-, 5-year: favor resection.Small HCC ≤ 3 cm:2-, 3-, 5-year: favor resection.	RFS:1-, 2-, 3-, 5-year: favor resection.	NA.	Complications: less in RFA.	Resection was superior to RFA and PEI for treatment of patients with early-stage HCC, but with more complications.
Qi	J Clin Gastroenterol (2014)	RFA vs surgical resection	OS:Favor resection.	RFS:Favor resection.	NA.	Complications: less in RFA.Hospital stay: longer in resection.	Resection might improve the OS and RFS in small HCC patients, but with more complications and longer hospital stay
Sun	World Chinese J Digestology (2011)	RFA vs surgical resection	OS:1-, 3-, 5-year: favor resection.	Tumor-free survival:1-, 3-, 5-year: favor resection.	NA.	Complications: less in RFA.	Resection has more complications, but a better overall efficacy.
Wang	PLoS One (2014)	RFA vs surgical resection	OS:RCT: 1-, 3-year: statistically similar.5-year: favor resection. NRCT: 1-, 3-, 5-year: favor resection.	DFS:RCT: statistically similar. NRCT: 1-, 3-, 5-year: favor resection.RFS:RCT: 1- and 3-year: statistically similar.5-year: favor resection.NRCT: 1-, 3-, 5-year: favor resection.	Recurrence: RCT: 1-year: statistically similar.3-, 5-year: favor resection.NRCT: favor resection.	In-hospital mortality:statistically similar.Complications: less in RFA.Hospital stay:longer in resection.	The effectiveness of RFA is comparable to resection with fewer complications but higher recurrence, especially for very early HCC.
Weis	Cochrane Database Syst Rev (2013)	RFA vs other therapeutic methods (including RFA vs surgical resection)	OS:RFA vs surgical resection: statistically similar (random effect model); favor resection (fixed effect model).	NA.	NA.	Duration of admission: RFA vs surgical resection: shorter in RFA.	Hepatic resection is superior to RFA regarding survival. However, RFA might be associated with fewer complications and a shorter hospital stay than hepatic resection.
Xu	World J Surg Oncol (2012)	RFA vs surgical resection	OS:1-, 3-, 5-year: favor resection.HCC < 3 cm:1-, 3-, 5-year: favor resection.	NA.	Recurrence: favor RFA.	Complications:less in RFA.	Resection had significantly improved survival benefits and lower complications for early HCC, especially for HCC ≤ 3 cm in diameter.
Zhou	Zhonghua Wai Ke Za Zhi (2011)	RFA vs surgical resection	OS:1-, 2-, 3-year: statistically similar.	DFS: 1-year: statistically similar.2-, 3-, 4-year: favor resection.	Recurrence: favor RFA.	Complications: less in RFA.	For small HCC within the Milan criteria, RFA had a similar OS to resection. RFA was less invasive with a lower postoperative morbidity.
Zhou	BMC Gastroenterol (2010)	RFA vs surgical resection	OS:1-, 2-, 5-year: statistically similar.3-year: favor resection.	DFS:1-, 3-, 5-year: favor resection.	Local intrahepatic recurrence: favor resection.Distant intrahepatic recurrence:statistically similar.	Postoperative morbidity: less in RFA.Postoperative mortality: statistically similar.	Resection was superior to RFA in the treatment of patients with small HCC eligible for surgical treatments, particularly for tumors > 3 cm.
**PEI vs resection**							
Hoshida	Hepatology (2000)	Surgical resection vs PEI and LT (including PEI vs resection)	OS:Surgical resection vs PEI: 3-year: statistically similar.	DFS:Surgical resection vs PEI: 3-year: statistically similar.	NA.	NA.	OS and DFS were comparable between PEI and resection.
Schoppmeyer	Cochrane Database Syst Rev (2009)	PEI vs PAI or surgery (including PEI vs surgery)	OS:PEI vs surgery:statistically similar.	RFS:PEI vs surgery:statistically similar.	NA.	NA.	Insufficient evidence for firm conclusions regarding comparison between PEI vs surgery.
**Non-surgical-resection ablation vs resection**							
Dong	World J Gastroenterol (2014)	Non-surgical-resection ablation vs surgical resection	OS:Non-surgical-resection ablation vs surgical resection:1-, 3-year: statistically similar.	DFS:Non-surgical-resection ablation vs surgical resection:1-, 3-year: statistically similar.	Local recurrence:Non-surgical-resection ablation vs surgical resection:At the end of follow-up: favor surgical resection.	Adverse events:Non-surgical-resection ablation vs surgical resection:Lower in surgical resection.	Surgical resection is superior to non-surgical ablation for the treatment of small HCC.
**RFA versus percutaneous ethanol injection (PEI) or percutaneous acetic acid injection (PAI)**							
Bouza	BMC Gastroenterol (2009)	RFA vs PEI	OS:1-, 2-, 3-, 4-year: favor RFA.	DFS: 1-, 2-, 3-year: favor RFA.	Local recurrence: less in RFA.Remote intrahepatic recurrence:statistically similar.	Tumor complete response:favor RFA.Total complications: less in PEI.Major complications: statistically similar.	The superiority of RFA versus PEI was supported, in terms of better survival and local control of the disease, for the treatment of patients with relatively preserved liver function and early-stage non-surgical HCC.
Cho	Hepatology (2009)	RFA vs PEI	OS:3-year: favor RFA.	NA.	NA.	NA.	RFA demonstrated significantly improved 3-year survival status for patients with HCC, when compared to PEI.
Dong	World J Gastroenterol (2014)	Surgical resection and non-surgical-resection ablation therapies (including RFA vs PEI)	OS:RFA vs PEI:1-year: statistically similar.2-, 3-year: favor RFA.	NA.	NA.	NA.	RFA is superior to PEI in term of 2- and 3-year OS.
Germani	J Hepatol (2010)	RFA, PEI, PAI (including RFA vs PEI and PAI)	OS:RFA vs PEI: favor RFA.RFA vs PAI: statistically similar.	NA.	Local recurrence:RFA vs PEI: less in RFA.RFA vs PAI: statistically similar.de novo tumours:RFA vs PEI: statistically similar.RFA vs PAI: statistically similar.	Complete necrosis:RFA vs PEI: less in RFA.Adverse events:RFA vs PEI: statistically similar.RFA vs PAI: statistically similar.Major complications:RFA vs PEI: statistically similar.	RFA seems to be a superior ablative therapy than PEI for HCC, particularly for tumours > 2 cm. RFA and PAI have similar survival rates.
Orlando	Am J Gastroenterol (2009)	RFA vs PEI	OS:1-, 2-, 3-year: favor RFA.	Cancer-free survival:1-, 2-, 3-year: favor RFA.	Local recurrence: less in RFA.	Complete tumor necrosis: better in RFA.	RFA is superior to PEI in the treatment of small HCC with respect to OS and DFS. RFA shows a significantly smaller risk of local recurrence.
Shen	J Gastroenterol Hepatol (2013)	RFA vs PEI	OS:3-year: favor RFA.	NA.	Local recurrence: less in RFA.	Major complications: less in PEI.	RFA appears superior to PEI with respect to 3-year survival for small HCCs < 3 cm. RFA was more feasible in patients with HCCs > 2 cm or Child–Pugh A liver function.
Weis	Cochrane Database Syst Rev (2013)	RFA vs other therapeutic methods (including RFA vs PEI or PAI)	OS:RFA vs PEI or PAI: favor RFA.	Event-free survival: RFA vs PEI: favor RFA.Local progression:RFA vs PEI: favor RFA.	NA.	Duration of admission: RFA vs PEI or PAI: statistically similar.	RFA seems superior to PEI regarding survival.
Xu	Eur J Med Res (2014)	RFA vs PEI	OS:1-, 2-, 3-year: favor RFA.	NA.	Local recurrence:1-, 2-, 3-year: less in RFA.	NA.	RFA is superior to PEI in better survival and local disease control for small HCCs < 5 cm in diameter.
**RFA versus cryosurgery ablation (CSA)**							
Huang	Hepato-gastroenterology (2013)	RFA vs CSA	OS:statistically similar.	NA.	Local recurrence:less in RFA.	Complications: less in RFA.	RFA is significantly superior to CSA.
**RFA versus laser ablation**							
Weis	Cochrane Database Syst Rev (2013)	RFA vs other therapeutic methods (including RFA vs laser ablation)	OS:RFA vs laser ablation: statistically similar.	Event-free survival: RFA vs laser ablation: statistically similar.Local progression:RFA vs laser ablation: statistically similar.	NA.	NA.	RFA seems to be similar to laser ablation. (Only one trial was identified.)
**RFA vs microwave ablation**							
Weis	Cochrane Database Syst Rev (2013)	RFA vs other therapeutic methods (including RFA vs microwave ablation)	NA.	Local progression:RFA vs microwave ablation: statistically similar.	NA.	Major complications:RFA vs microwave ablation: statistically similar.	RFA seems to be similar to microwave ablation. (Only one trial was identified.)
**RFA vs any other therapeutic methods**							
Huang	Zhonghua Nei Ke Za Zhi (2008)	RFA vs other therapeutic methods	OS:3-year: favor RFA.	NA.	Local recurrence:favor RFA.Intrahepatic metastasis:statistically similar.Extrahepatic metastasis:statistically similar.	Severe adverse events:statistically similar.	RFA is superior to other treatment methods with respect to local recurrence and 3-year overall survival in early HCC and is the preferred therapeutic methods for small HCC.
**PEI versus PAI**							
Germani	J Hepatol (2010)	RFA, PEI, PAI (including PAI vs PEI)	OS:PAI vs PEI: statistically similar.	NA.	Local recurrence:PAI vs PEI: statistically similar.de novo tumours:PAI vs PEI: statistically similar.	Complete necrosis:PAI vs PEI: statistically similar.Adverse events:PAI vs PEI: statistically similar.	PAI did not differ significantly from PEI for all the outcomes evaluated.
Schoppmeyer	Cochrane Database Syst Rev (2009)	PEI vs PAI or surgery (including PEI vs PAI)	OS:PEI vs PAI: statistically similar.	RFS:PEI vs PAI: statistically similar.	NA.	Hospital stay:PEI vs PAI: no firm conclusions.	PEI and PAI do not differ significantly regarding benefits and harms in patients with early HCC.
**RFA + TACE versus RFA or TACE alone**							
Dong	World J Gastroenterol (2014)	Surgical resection and non-surgical-resection ablation therapies (including RFA vs RFA+TACE)	OS:RFA vs RFA+TACE:1-, 3-year: statistically similar.5-year: favor RFA+TACE.	NA.	NA.	NA.	RFA in combination with TACE can improve the 5-year OS.
Han	J Intervent Radiol (China) (2013)	RFA + TACE vs TACE	OS:1-, 2-, 3-year: favor RFA+TACE.	NA.	NA.	NA.	RFA plus TACE can significantly improve the long-term survival rate.
Jiang	Tumour Biol (2014)	RFA + TACE vs RFA alone	OS:1-, 3-year: favor RFA+TACE.	NA.	NA.	NA.	RFA plus TACE improve the survival rates compared with RFA alone for patients with HCC.
Kong	Tumour Biol (2014)	RFA + TACE vs RFA alone	OS:1-, 3-, 5-year: favor RFA+TACE.	NA.	NA.	NA.	The combination of RFA with TACE has advantages in improving OS.
Liao	PLoS One (2013)	TACE + 3D-CRT, HIFU, PEI, RFA, or RT vs TACE alone (including TACE+RFA vs TACE)	OS:RCT:1-year: TACE+RFA vs TACE: statistically similar.	NA.	NA.	Adverse events:NA. (systematic reviews, but not meta-analyses).	TACE combined with RFA could not improve the OS status, as compared with TACE alone.
Liu	Tumour Biol (2014)	RFA + TACE vs RFA alone	OS:1-, 3-year: favor RFA+TACE.	RFS: 1-, 3-year: favor RFA+TACE.	NA.	Major complications: statistically similar.	The combination of RFA with TACE can improve the OS and RFS rates for patients with HCC.
Lu	Eur J Gastroenterol Hepatol (2013)	RFA + TACE vs RFA alone	OS:1-, 3-year: favor RFA+TACE.5-year: statistically similar. > 3 cm: 1-, 3-, 5-year: favor RFA+TACE. < 3 cm: statistically similar.	NA.	NA.	Major complications: statistically similar.	RFA plus TACE improve the survival rates compared with RFA alone for patients with HCC > 3 cm.
Ni	World J Gastroenterol (2013)	RFA + TACE vs RFA alone	OS:1-, 2-, 3-year: favor RFA+TACE.5-year: statistically similar. < 3 cm: 1-, 3-year: statistically similar.3–5 cm: 1-, 3-, 5-year: favor RFA+TACE. > 5 cm: 1-, 3-year: favor RFA+TACE.	RFS: 3-, 5-year: favor RFA+TACE.1-year: statistically similar.Progression rate: less in RFA + TACE.	NA.	Major complications: statistically similar.	The combination of RFA with TACE has advantages in improving OS, and provides better prognosis for patients with intermediate- and large-size HCC.
Ni	J Cancer Res Clin Oncol (2013)	RFA + TACE vs RFA or TACE alone	OS:1-, 3-year: favor RFA+TACE.	RFS: 1-year: statistically similar.3-year: favor RFA+TACE.	NA.	NA.	The combination of TACE and RFA has better effectiveness than that of TACE and RFA monotherapy in the treatment for patients with HCC.
Yan	Dig Dis Sci (2012); Dig Dis Sci (2013) duplicates	RFA + TACE vs RFA alone	OS:1-, 3-, 5-year: favor RFA+TACE.	NA.	NA.	NA.	The combination of TACE with RFA can improve the OS and provides better prognosis for patients with HCC.
Zhao	J Intervent Radiol (China) (2013)	RFA + TACE vs RFA or TACE alone	OS:1-, 2-, 3-year: favor RFA+TACE.	NA.	Local recurrence:favor RFA+TACE.	NA.	RFA plus TACE is superior to TACE or RFA monotherapy.
**PEI + TACE versus monotherapy**							
Liao	PLoS One (2013)	TACE + 3D-CRT, HIFU, PEI, RFA, or RT vs TACE alone (TACE + PEI vs TACE)	OS:RCT:1-year:TACE+PEI vs TACE: statistically similar.3-year:TACE+PEI vs TACE: favor TACE+PEI.Observational studies:1-year:TACE+PEI vs TACE: favor TACE+PEI.3-year:TACE+PEI vs TACE: statistically similar.	NA.	NA.	Adverse events:NA. (systematic reviews, but not meta-analyses).	TACE combined with PEI could improve the OS status than performing TACE alone.
Wang	Med Oncol (2011)	TACE + PEI vs TACE alone	OS:0.5-, 1-, 2-, 3-year: favor TACE + PEI.	NA.	NA.	OS:Decline rates of the AFP level: favor TACE + PEI.Reduction rates of tumor size: favor TACE + PEI.	The efficacy of TACE combined with PEI is significantly better than that of TACE alone.
Wang	Liver Int (2010)	TACE + percutaneous ablation therapy (RFA or PEI) vs TACE or percutaneous ablation therapy alone (TACE + PEI vs PEI)	TACE+PEI vs TACE: 1-, 2-, 3-year: favor TACE+PEI.TACE+PEI vs PEI: 1-, 2-year: favor TACE+PEI.3-year: statistically similar.	NA.	NA.	NA.	TACE combined with PEI improved the OS status for large HCCs.
**Any local ablative therapy + TACE versus monotherapy**							
Gu	J Cancer Res Clin Oncol (2014)	TACE + local ablative therapy vs monotherapy	OS:1-, 2-, 3-, 5-year: favor TACE + local ablative therapy.OS: favor TACE + local ablative therapy.	NA.	NA.	Tumor response: favor TACE + local ablative therapy.	The combination of TACE with local ablative therapy was superior to monotherapy in the treatment for patients with HCC.
Wang	Liver Int (2010)	TACE + percutaneous ablation therapy (RFA or PEI) vs TACE or percutaneous ablation therapy alone	Combination therapy vs monotherapy:1-, 2-, 3-year: favor TACE + percutaneous ablation therapy.TACE+percutaneous ablation vs TACE: 1-year: favor TACE + percutaneous ablation.	NA.	Recurrence: Combination therapy vs monotherapy: favor TACE + percutaneous ablation therapy.	NA.	TACE combined with percutaneous ablation therapy improved the OS status for large HCCs.

**Table 5:** Findings of meta-analyses: An overview of included studies regarding TACE

First author	Journal (Year)	Comparisons	OS	DFS, RFS, TTP, PFS	Recurrence, time to recurrence	Other endpoints	Major comments
**TACE/TAE versus no treatment**							
Camma	Radiology (2002)	TACE or TAE vs non-active treatment; different transarterial modalities of therapy	TACE or TAE vs non-active treatment: 2-year mortality: lower in TACE or TAE.TAE vs transarterial chemotherapy: overall mortality: lower in TAE.	NA.	NA.	NA.	In patients with unresectable HCC, chemoembolization significantly improved the overall 2-year OS compared with nonactive treatment, but the magnitude of the benefits was relatively small.
Geschwind	Am J Clin Oncol (2003)	Therapeutic embolization vs supportive care alone	OS:3-, 6-month: statistically similar.	NA.	NA.	NA.	The data fail to show a survival advantage associated with therapeutic embolization versus supportive care alone in patients with unresectable HCC.
Leng	ANZ J Surg (2014)	TACE vs control treatment	OS:1-year: better in TACE.	NA.	NA.	NA.	TACE improves the 1-year survival of patients with HCC and PVTT.
Llovet	Hepatology (2003)	TACE or tamoxifen vs control treatment (including TACE versus no treatment)	OS:TACE vs control treatment:2-year: favor TACE.	NA.	NA.	NA.	Chemoembolization improves survival of patients with unresectable HCC and may become the standard treatment.
Marelli	Cardiovasc Intervent Radiol (2007)	Transarterial therapy vs conservative management	TACE, TAE, or transarterial oily chemoembolization versus conservative treatment: mortality: lower in TACE, TAE, or transarterial oily chemoembolization.	NA.	NA.	NA.	TACE improves survival.
Oliveri	Cochrane Database Syst Rev (2011)	TACE or TAE vs placebo, sham, or no intervention	OS:statistically similar.	NA.	NA.	NA.	No firm evidence to support or refute TACE or TAE for patients with unresectable HCC.
Xue	BMC Gastroenterol (2013)	TACE vs conservative treatment	OS:6-month, 1-year: favor TACE.	NA.	NA.	NA.	TACE, as a safe treatment, has potential for incurring a survival benefit for advanced HCC with PVTT, even with MPV obstruction.
**TACE versus TAE**							
Camma	Radiology (2002)	TACE or TAE vs non-active treatment; different transarterial modalities of therapy (including TAE vs TACE)	TAE vs TACE: overall mortality: statistically similar.	NA.	NA.	NA.	The addition of an anticancer drug did not improve the therapeutic benefit of TAE.
Marelli	Cardiovasc Intervent Radiol (2007)	Transarterial therapy vs conservative management;different transarterial modalities of therapy (including TACE vs TAE)	TACE vs TAE: statistically similar.	NA.	NA.	NA.	TAE appears as effective as TACE.
Xie	Tumour Biol (2014)	TACE vs TAE	OS:statistically similar.	NA.	NA.	Adverse events: higher in TACE (no statistical comparison).	The efficacy of TACE is not superior to TAE in advanced HCC patients. Moreover, TACE was associated with an increased rate of adverse events than TAE.
**Doxorubicin-eluting bead (DEB)-TACE versus conventional TACE (cTACE)**							
Gao	Hepato-gastroenterology (2013)	DEB-TACE vs cTACE	NA.	NA.	NA.	Complete or partial response, stable or progressive disease, disease control: statistically similar.	DEB-TACE is able to accomplish the same tumor response as conventional TACE.
Han	PLoS One (2014)	DEB-TACE vs cTACE	NA.	NA.	NA.	Disease control: statistically similar.Complications: statistically similar.Severe complications: statistically similar.	DEB-TACE has the same disease control rate without an increase in complications and severe complications.
Huang	J Gastroenterol Hepatol (2014)	DEB-TACE vs cTACE	OS:1-, 2-year: better in DEB-TACE.6-month, 3-year: statistically similar.	NA.	NA.	Objective tumor response: better in DEB-TACE.Adverse side effect: similar (no statistical comparison).	DEB-TACE provides significantly better tumor response compared with cTACE; DEB-TACE is as safe as cTACE
**TACE versus microsphere embolization**							
Xie	J Cancer Res Clin Oncol (2012)	TACE vs microsphere embolization	OS: favor microsphere embolization.1-year: Total analysis: favor microsphere embolization.Yttrium 90 (^90^Y) microspheres: statistically similar.^32^P glass microspheres: favor ^32^P glass microspheres.	TTP: longer in microsphere embolization.	NA.	Tumor response: Total analysis: better in microsphere embolization.Yttrium 90 (90Y) microspheres: statistically similar.^32^P glass microspheres: favor ^32^P glass microspheres.	Microsphere embolization treatment of patients with surgically unresectable HCC provided much better survival and treatment response than that of TACE.
**TACE + sorafenib versus TACE alone**							
Fu	J Cancer Res Clin Oncol (2014)	TACE + sorafenib vs TACE alone	OS:6-month, 1-year: favor TACE+sorafenib.2-year: statistically similar.	3-, 6-month progression free rate:Lower in TACE+sorafenib.	NA.	Objective response ratio & clinical benefit ratio:better in TACE+sorfafenib.Complications:higher in TACE+sorfafenib.	Combination of sorafenib and TACE showed survival and clinical benefits in patients with HCC, though enhanced morbidity.
Liu	PLoS One (2014)	TACE + sorafenib vs monotherapy	OS:statistically similar.	TTP:Longer in TACE+sorafenib.	NA.	NA.	Combination therapy may bring benefits for unresectable HCC patients in terms of TTP but not OS.
Yang	Mol Biol Rep (2014)	TACE + sorafenib vs TACE alone	All studies:favor TACE+sorafenib.RCTs: statistically similar.Retrospective studies:favor TACE+sorafenib.	TTP: All studies: favor TACE+sorafenib.RCTs: favor TACE+sorafenib.Retrospective studies: favor TACE+sorafenib.	NA.	Response to treatment: All studies: favor TACE+sorafenib.RCTs: favor TACE+sorafenib.Retrospective studies: favor TACE+sorafenib.	When compared with TACE monotherapy, the combination of TACE and sorafenib is likely to improve OS, TTP and response to treatment, but with more sorafenib-related adverse events.
Zhang	PLoS One (2014)	TACE + sorafenib vs TACE alone	OS:favor TACE+sorafenib.	TTP: longer in TACE+sorafenib.PFS: statistically similar.	NA.	Objective response ratio: better in TACE+sorafenib.Complications: higher in TACE+sorfafenib.	The combination therapy of TACE plus sorafenib in patients with intermediate or advanced stage of HCC, can improve the OS, TTP, and objective tumor response, but with a significantly increased risk of adverse reactions.
**TACE + HIFU versus TACE**							
Cao	Ultrasound Med Biol (2011)	TACE + HIFU vs TACE alone	OS:favor TACE + HIFU.	NA.	NA.	Tumor response: better in TACE + HIFU.	Combined therapy was more therapeutically beneficial.
Liao	PLoS One (2013)	TACE + 3D-CRT, HIFU, PEI, RFA, or RT vs TACE alone (including TACE+HIFU vs TACE)	OS:RCT:1-year:TACE+HIFU vs TACE: favor TACE+HIFU.3-year:TACE+HIFU vs TACE: statistically similar.Observational studies:1-year:TACE+HIFU vs TACE: favor TACE+HIFU.3-year:TACE+HIFU vs TACE: favor TACE+HIFU.	NA.	NA.	Adverse events:NA. (systematic reviews, but not meta-analyses).	TACE combined with HIFU could improve the OS status than TACE alone.
**TACE + thermotherapy versus TACE**							
Li	Chinese Journal of Evidence-Based Medicine (2012)	TACE + thermotherapy vs TACE alone	OS:1-, 2-year:favor TACE + thermotherapy.0.5-, 1.5-, 3-year:statistically similar.	NA.	NA.	Overall effective rate: better in TACE + HIFU.Quality of life: better in TACE + HIFU.	Combination therapy can improve long-term survival rate, effective rate, and quality of life.
**TACE + argon-helium cryotherapy system (AHCS) versus TACE**							
Li	Chinese Journal of Evidence-Based Medicine (2013)	TACE + AHCS vs TACE alone; TACE + AHCS vs AHCS	OS:TACE + AHCS vs TACE alone:0.5-, 1-, 1.5-, 2-, 2.5-year: favor TACE + AHCS.3-year: statistically similar.TACE + AHCS vs AHCS alone:0.5-, 1.5-, 2-, 2.5-year: favor TACE + AHCS.1-, 3-year: statistically similar.	NA.	NA.	TACE + AHCS vs TACE alone:Total effective rate, complete necrosis rate, recurrence: favor TACE + AHCS.AFP reduction and CD4 improvement: Favor TACE + AHCS.Adverse events: statistically similar.TACE + AHCS vs AHCS alone:AFP reduction and CD4 improvement: favor TACE + AHCS.	Compared with the TACE or AHCS alone, TACE combined with AHCS can improve long-term survival rate and short-term curative effect, and improve the patients' immunity.
**TACE + radiotherapy versus TACE alone**							
Liao	PLoS One (2013)	TACE + 3D-CRT, HIFU, PEI, RFA, or radiotherapy vs TACE alone (including TACE+radiotherapy vs TACE)	OS:RCT:1-year:TACE+radiotherapy vs TACE: favor TACE+radiotherapy.3-year:TACE+radiotherapy vs TACE: favor TACE+radiotherapy.Observational studies:1-year:TACE+radiotherapy vs TACE: favor TACE+radiotherapy.3-year:TACE+radiotherapy vs TACE: favor TACE+radiotherapy.	NA.	NA.	Adverse events:NA. (systematic reviews, but not meta-analyses).	TACE combined with radiotherapy could improve the OS status than TACE alone.
Meng	Radiother Oncol (2009)	TACE + radiotherapy vs TACE alone	OS:1-, 2-, 3-, 5-year: favor TACE + radiotherapy.	NA.	NA.	Tumor response: favor TACE + radiotherapy.Nausea/vomit: statistically similar.Leukocyte count declined: statistically similar.Alanine aminotransferase level increased: statistically similar.Total bilirubin level increased: higher in TACE + radiotherapy.	TACE + radiotherapy was more therapeutically beneficial.
**TACE + three-dimensional conformal radiation therapy (3D-CRT) versus TACE alone**							
Liao	PLoS One (2013)	TACE + 3D-CRT, HIFU, PEI, RFA, or RT vs TACE alone (including TACE+3D-CRT vs TACE)	OS:RCT:1-year:TACE+3D-CRT vs TACE: favor TACE+3D-CRT.3-year:TACE+3D-CRT vs TACE: favor TACE+3D-CRT.Observational studies:1-year:TACE+3D-CRT vs TACE: favor TACE+3D-CRT.3-year:TACE+3D-CRT vs TACE: favor TACE+3D-CRT.	NA.	NA.	Adverse events:NA. (systematic reviews, but not meta-analyses).	TACE combined with 3D-CRT could improve the OS status than TACE alone.
**TACE + traditional Chinese medicine (TCM) versus TACE alone**							
Cheung	Evid Based Complement Alternat Med (2013)	TACE + TCM vs TACE alone	OS:6-, 12-, 18-, 24-, 36-month: favor TCM.	NA.	NA.	Tumor response: favor TCM.Quality of life using KPS: favor TCM.TACE toxicity: favor TCM.	The use of TCM may increase the efficacy and reduce the toxicity of TACE in treating patients with unresectable HCC. TCM could be considered as an adjuvant therapy for unresectable HCC patients during TACE.
Cho	Expert Opin Investig Drugs (2009)	TACE + Chinese herbal therapy vs TACE alone	OS:1-, 2-, 3-year: favor TCM.6-month: statistically similar.	NA.	NA.	Tumor response: favor TCM.Quality of life: favor TCM.Immunolisation: favor TCM.Recovery of liver function: favor TCM.AFP concentration: lower in TCM.Reduction in chemotherapy toxicities: favor TCM.	The use of TCM to enhance the efficacy of TACE in HCC patients.
Meng	J Altern Complement Med (2008)	TACE + TCM vs TACE alone	OS:6-, 12-, 24-, 36-month: favor TACE+TCM	NA.	NA.	Tumor response: favor TACE+TCM.Symptom alleviation: favor TACE+TCM.Quality of life by Karnofsky performance score: favor TACE+TCM.Bone toxicity rate: favor TCM.	TCM plus TACE, compared with TACE alone, was more therapeutically beneficial.
Meng	Explore (NY) (2011)	TACE + TCM vs TACE alone	NA.	NA.	NA.	Proportion of CD3+ T cells: favor TACE + TCM.Proportion of CD4+ T cells: favor TACE + TCM.Proportion of CD8+ T cells: statistically similar.Ratio of CD4 / CD8: favor TACE + TCM.Proportion of NK cells: favor TACE + TCM.Adverse events: decreased in TACE + TCM.	TCM in combination with TACE improves the immune response of patients with unresectable HCC.
Sun	Afr J Tradit Complement Altern Med (2012)	TACE + Compound Kushen Injection vs TACE alone	1-year OS:favor TACE + Compound Kushen Injection.	NA.	NA.	Quality of life with KPS improvement:favor TACE + Compound Kushen Injection.Tumor response:favor TACE + Compound Kushen Injection.	Compound Kushen Injection plus TACE is superior to TACE alone for unresectable HCC.
Wu	J Cancer Res Ther (2014)	TACE + Cinobufacini vs TACE only	OS:1-year: statistically similar.2-year: favor TACE + Cinobufacini.	NA.	NA.	Objective response rate: favor TACE + Cinobufacini.	Cinobufacini combined with TACE can significantly increase the objective response rate and 2-year survival rate compared with TACE only in patients with advanced HCC.
**TACE + cytokine-induced killer (CIK) cell therapy vs TACE alone**							
Chen	Chinese-German J Clin Oncol (2013)	TACE + CIK cell therapy vs TACE alone	OS:favor CIK cell + TACE.0.5-, 1-, 2-year: favor CIK cell + TACE.	TTP:favor CIK cell + TACE.	NA.	Quality of life with KPS improvement:favor CIK cell + TACE.Liver function improvement:favor CIK cell + TACE.Immune function improvement:favor CIK cell + TACE.	CIK cells combined with TACE therapy demonstrated a significant superiority in improving recent and forward curative effects, immunity function, quality of life and liver function of HCC patients.
Li	Clin Res Hepatol Gastroenterol (2014)	CIK cell therapy+TACE+RFA vs TACE+RFA; CIK cell therapy+TACE vs TACE	OS:CIK+TACE+RFA vs TACE+RFA:1-, 2-, 3-year: favor CIK+TACE+RFA.CIK+TACE vs TACE:0.5-, 1-, 2-year: favor CIK+TACE.	RFS:CIK+TACE+RFA vs TACE+RFA:1-year: favor CIK+TACE+RFA.	NA.	NA.	CIK cells transfusion therapy truly showed a synergistic effect for HCC patients after minimally invasive treatment especially for a long-term survival.

### LT

#### Living donor LT (LDLT) versus deceased donor LT (DDLT)

Three meta-analyses compared the outcomes of LDLT versus DDLT [8, 41, 67]. All of them demonstrated that the OS was statistically similar between the two groups [8, 41, 67]. Two of them showed that the 1-, 3-, and 5-year DFS were statistically similar between the two groups [8, 67], but another one favored DDLT in term of DFS [41]. One of them found that the recurrence was statistically similar between the two groups [67]; by comparison, another one favored LDLT in term of 5-year recurrence, but not 1- or 3-year recurrence [8].

Only non-RCT studies, rather than RCTs, were included in the three meta-analyses.

The meta-analysis by Grant had a larger number of included studies than those by Al Hasan and Liang (16 versus 7 and 7) (Supplementary Table S9). Notably, there was an overlap of included studies between the two meta-analyses by Liang and Grant. All studies which were included in the meta-analysis by Liang were also covered by the meta-analysis by Grant. The meta-analysis by Al Hasan did not show the included studies.

Given its superiority in the quantity of non-RCT studies, the results of the meta-analysis by Grant might be more reliable. In details, LDLT has lower DFS than DDLT.

#### Primary versus salvage LT

Two meta-analyses compared the outcomes of primary versus salvage LT [59, 157]. Both of them demonstrated that the OS and 1- and 3-year DFS were statistically similar between the two groups [59, 157]. One of them favored primary LT in term of 5-year DFS [157]; by comparison, another one showed that the 5-year DFS was statistically similar between the two groups [59]. In addition, salvage LT had significantly longer operative time, increased intra-operative blood loss, and larger number of transfused units of packed red blood cells than primary LT [157]. But the length of hospital and ICU stay was statistically similar between the two groups [157].

Only non-RCT studies, rather than RCTs, were included in the two meta-analyses.

The meta-analysis by Zhu had a larger number of included studies than that by Li (14 versus 11) (Supplementary Table S10). Notably, there was an overlap of included studies between them. All studies which were included in the meta-analysis by Li were also included in the meta-analysis by Zhu.

Given its superiority in the quantity of non-RCT studies, the results of the meta-analysis by Zhu might be more reliable. In details, salvage LT achieves the same short- and long-term survival as primary LT. However, primary LT was significantly superior to salvage LT in terms of operative time, blood loss, and blood transfusion.

#### Sirolimus-based immunosuppression after LT

Two meta-analyses compared the outcomes of sirolimus-based immunosuppression versus no sirolimus after LT [66, 83]. Both of them favored the use of sirolimus after LT in terms of OS, DFS/RFS, and recurrence [66, 83].

Only non-RCT studies, rather than RCTs, were included in the two meta-analyses.

Both of them had a similar number of included studies (5 versus 5) (Supplementary Table S11). But not all included studies were the same between them.

The results were completely consistent between the two meta-analyses. In details, the use of sirolimus after LT should be favored.

#### LT versus surgical resection

Seven meta-analyses compared the outcomes of LT versus surgical resection [25, 46, 96, 98, 129, 131, 146]. There were 4, 4, 6, and 1 meta-analyses to compare the 1-, 3-, 5-, and 10-year survival, respectively. As for the 1-year survival, three of them demonstrated that the survival was statistically similar between the two groups [129, 131, 146], but another one favored surgical resection [98]. As for the 3-year survival, two of them found that the survival was statistically similar between the two groups [46, 131], but another two favored LT [129, 146]. As for the 5-year survival, two of them showed that the survival was statistically similar between the two groups [96, 98], but another four favored LT [25, 129, 131, 146]. As for the 10-year survival, the only one meta-analysis favored LT [98]. There were 3, 3, 4, and 1 meta-analyses to compare the 1-, 3-, 5-, and 10-year DFS, respectively. As for the 1-year DFS, two of them favored LT [131, 146], but another one found that the 1-year DFS was statistically similar between the two groups [98]. As for the 3-year DFS, all of them favored LT [46, 131, 146]. As for the 5-year DFS, all of them favored LT [98, 129, 131, 146]. As for the 10-year DFS, the only one meta-analysis favored LT [98]. Two meta-analyses compared the recurrence. Both of them favored LT in term of recurrence [129, 146].

Only non-RCT studies, rather than RCTs, were included in these meta-analyses.

The meta-analysis by Zhang had the largest number of included studies (*n* = 62) (Supplementary Table S12). By comparison, the number of included studies was less than 20 in 6 other meta-analyses.

Given its superiority in the quantity of non-RCT studies, the results of the meta-analysis by Zhang might be more reliable. In details, LT provides a significantly better survival and a lower recurrence.

## Surgical resection

### Surgical resection margin 1 cm versus 2 cm

Only one meta-analysis compared the outcomes of hepatectomy with a margin aiming at 2 cm versus those with a margin aiming at 1 cm [109]. Regardless of study design, the 1-year survival was statistically similar between the two groups [109]. In the subgroup analysis of randomized studies, the 3- and 5-year survival and DFS were better in patients undergoing hepatectomy with a margin aiming at 2 cm than in those with a margin aiming at 1 cm [109]. Contrarily, in the subgroup analysis of non-randomized studies, the 3- and 5-year survival and DFS were statistically similar between the two groups [109].

One RCT and 4 non-RCT studies were included in this meta-analysis.

### Laparoscopic versus open resection

Nine meta-analyses compared the outcomes of laparoscopic versus open resection [30, 62, 94–95, 111, 126, 135, 137, 156]. All of them demonstrated that the OS and DFS/RFS at any time points were statistically similar between the two groups [30, 62, 94–95, 111, 126, 135, 137, 156]. Two of them also found that the recurrence was statistically similar between the two groups [62, 126]. Eight of them demonstrated statistically similar operative time between the two groups [30, 62, 95, 111, 126, 135, 137, 156], but one demonstrated significantly longer operative time in laparoscopic resection group [94]. All of them demonstrated that blood loss or intraoperative bleeding was significantly less in laparoscopic resection group [30, 62, 94–95, 111, 126, 135, 137, 156]. Among the 7 meta-analyses evaluating the blood transfusion, 6 demonstrated significantly less blood transfusion in laparoscopic resection group [30, 62, 111, 126, 137, 156], and one demonstrated statistically similar blood transfusion between the two groups [94]. Among the 6 meta-analyses evaluating the overall complications, 5 demonstrated significantly less complications in laparoscopic resection group [30, 62, 94, 111, 135], and one demonstrated statistically similar complications between the two groups [126]. Among the 8 meta-analyses evaluating the hospital length, all demonstrated significantly shorter hospital study in laparoscopic resection group [30, 62, 94, 111, 126, 135, 137, 156].

Only non-RCT studies, rather than RCTs, were included in these meta-analyses.

The meta-analyses by Park, Xiong, and Yin had the largest number of included studies (*n* = 15) followed by the meta-analyses by Yao (*n* = 13), Zhou (*n* = 10), Li (*n* = 10), Fancellu (*n* = 9), Pang (*n* = 7), and Twaij (*n* = 4) (Supplementary Table S13). The included studies were completely same between the two meta-analyses by Xiong and Yin. However, the studies included in the meta-analysis by Park were different from those included in the meta-analyses by Xiong and Yin.

Given its superiority in the quantity of non-RCT studies, the results of the meta-analyses by Park, Xiong, and Yin might be more reliable. In details, they suggested that the operative time was statistically similar between the two groups and that laparoscopic resection was superior to open resection in terms of blood loss, blood transfusion, complications, and hospital stay.

### Anatomic resection versus non-anatomic resection

Six meta-analyses compared the outcomes of anatomic versus non-anatomic resection [15, 23, 60, 110, 136, 152]. Four of them demonstrated that the OS was statistically similar between the two groups [15, 60, 110, 136], but another two favored anatomic resection in term of 5-year survival [23, 152]. One of them found that the DFS was statistically similar between the two groups [60], but another four favored anatomic resection in term of DFS [15, 23, 136, 152]. Two of them showed that the recurrence was statistically similar between the two groups [15, 110], but another two favored anatomic resection in term of local intrahepatic recurrence [136, 152]. Post-operative complications were statistically similar between the two groups [23, 110, 136, 152].

Only non-RCT studies, rather than RCTs, were included in these meta-analyses.

The meta-analysis by Cucchetti had the largest number of included studies (*n* = 18) followed by the meta-analyses by Zhou (*n* = 16), Tang (*n* = 12), Ye (*n* = 11), Chen (*n* = 9), and Li (*n* = 4) (Supplementary Table S14).

Given its superiority in the quantity of non-RCT studies, the results of the meta-analysis by Zhang might be more reliable. In details, anatomic resection was superior to non-anatomic resection in terms of OS and DFS.

#### Surgical resection + I^131^ lipiodol versus surgical resection alone

Two meta-analyses compared the outcomes of surgical resection in combination with I^131^ lipiodol versus surgical resection alone [36, 40]. Both of them favored the combination therapy in terms of OS, DFS, and recurrence [36, 40].

Only non-RCT studies, rather than RCTs, were included in these meta-analyses.

The meta-analysis by Gong had a larger number of included studies than that by Furtado (10 versus 5) (Supplementary Table S15). Notably, there was an overlap of included studies between them. All studies which were included in the meta-analysis by Furtado were also included in the meta-analysis by Gong.

The results were completely consistent between the two meta-analyses. In details, surgical resection in combination with I^131^ lipiodol should be favored.

## Surgical resection + TACE versus surgical resection alone

### Pre-operative TACE

Four meta-analyses compared the outcomes of surgical resection in combination with pre-operative TACE versus surgical resection alone [17, 116, 138, 153]. All of them found that the OS, DFS, and recurrence were statistically similar between the two groups [17, 116, 138, 153].

RCT studies were included in the meta-analyses by Cheng (*n* = 4), Wang (*n* = 3), and Zhou (*n* = 4), but not in the meta-analysis by Yu (*n* = 0).

The meta-analysis by Zhou had the largest number of included studies (*n* = 21) followed by the meta-analyses by Yu (*n* = 7), Cheng (*n* = 4), and Wang (*n* = 3) (Supplementary Table S16). All studies which were included in the two meta-analyses by Cheng and Wang were also included in the meta-analysis by Zhou.

The results were completely consistent between the two meta-analyses. In details, pre-operative TACE did not improve the OS or DFS.

### Post-operative TACE

Two meta-analyses compared the outcomes of surgical resection in combination with post-operative TACE versus surgical resection alone [17, 149]. Both of them favored post-operative TACE in terms of OS, DFS, and recurrence [17, 149].

Only RCT studies were included in the two meta-analyses.

Although the number of included studies was the same between the two meta-analysis by Cheng and Zhong (*n* = 6) (Supplementary Table S17), not all included studies were the same between them.

The results were completely consistent between the two meta-analyses. In details, post-operative TACE should be favored.

### Surgical resection + adjuvant chemotherapy versus surgical resection alone

Five meta-analyses compared the outcomes of surgical resection in combination with adjuvant chemotherapy versus surgical resection alone [78, 92, 112, 147–148].

### Oral systemic chemotherapy

Oral systemic chemotherapy was evaluated in two meta-analyses [78, 148]. The OS, RFS, and recurrence were statistically similar between patients with and without chemotherapy [78, 148].

RCT studies were included in the meta-analyses by Zhong (*n* = 3) and Mathurin (*n* = 1).

The meta-analysis by Zhong had a larger number of included studies than that by Mathurin (3 versus 2) (Supplementary Table S18). Not all included studies were the same between them.

The results were completely consistent between the two meta-analyses. In details, the adjunctive use of oral systemic chemotherapy should not be favored in patients undergoing surgical resection.

### Transarterial chemotherapy

Transarterial chemotherapy was evaluated in one meta-analysis [78]. As for the pre-operative transarterial chemotherapy, the overall analysis of both RCTs and non-RCTs demonstrated that chemotherapy improved the 2-year survival, but not the 1- or 3-year survival. The subgroup analysis of RCTs showed that the 1-, 2-, and 3-year recurrence were statistically similar between the two groups. As for the post-operative transarterial chemotherapy, the overall analysis of both RCTs and non-RCTs demonstrated that chemotherapy improved the 1-, 2-, and 3-year survival. The subgroup analysis of RCTs showed that chemotherapy improved the 2- and 3-year survival, but not the 1-year survival.

Approaches of chemotherapy were mixed in three meta-analyses [92, 112, 147]. The statistical results were largely inconsistent among them. One of them favored the chemotherapy in term of OS [147]; one showed that the OS was statistically similar between the two groups [112]; one demonstrated that the OS was decreased by chemotherapy [92].

RCT studies were included in the meta-analyses by Zheng (*n* = 13), Wang (*n* = 8), and Ono (*n* = 3).

The meta-analysis by Zheng had a larger number of included studies than those by Wang and Ono (48 versus 8 and 3) (Supplementary Table S19).

Given its superiority in the quantity of RCT studies, the results of the meta-analysis by Zheng might be more reliable.

### Surgical resection + immunotherapy versus surgical resection alone

Four meta-analyses compared the outcomes of surgical resection in combination with immunotherapy versus surgical resection alone [32, 75, 112, 124]. All of them demonstrated that the OS was statistically similar between the two groups [32, 75, 112, 124]. One of them favored the combination therapy in term of RFS. One of them favored the combination therapy in terms of 1- and 3-year recurrence [124]; one favored the combination therapy in term of 1-year recurrence, but not 3-year recurrence [75]; one showed that the recurrence was statistically similar between the two groups [32].

Only RCT studies were included in the meta-analyses by Xie (*n* = 6), Ma (*n* = 4), Wang (*n* = 3), and Flores (*n* = 2).

The meta-analysis by Xie had the largest number of included studies followed by those by Ma, Wang, and Flores (6 versus 4, 3, and 2) (Supplementary Table S20). Notably, there was an overlap of included studies among them.

Given its superiority in the quantity of RCT studies, the results of the meta-analysis by Xie might be more reliable. In details, the adjunctive use of immunotherapy might not be favored in patients undergoing surgical resection.

### Surgical resection + PVE versus surgical resection alone

One meta-analysis compared the outcomes of surgical resection in combination with PVE versus surgical resection alone [145]. The 1-, 3-, and 5-year survival and intrahepatic and distant recurrence were statistically similar between the two groups [145].

Only non-RCT studies were included in the meta-analysis by Zhao.

## Ablation therapy

### RFA versus surgical resection

Eighteen meta-analyses compared the outcomes of RFA versus surgical resection [11, 16, 24, 27, 31, 33, 47, 61, 69, 72, 89, 97, 106, 117, 119, 127, 151, 153]. As for the OS, seven of them favored surgical resection [27, 31, 47, 61, 97, 106, 127]; four demonstrated that the OS was statistically similar between the two groups [11, 16, 119, 151]; four showed that the 1-year survival was statistically similar between the two groups, but the 5-year survival was better in surgical resection group [33, 72, 89, 117]; one found that the 1- and 5-year survival were statistically similar between the two groups, but the 3-year survival was better in surgical resection group [155]; one reported that surgical resection had better OS than RFA in the subgroup analyses of a single nodule 3–5 cm and ≤ 3 cm, but the OS was statistically similar between the two groups in the subgroup analyses of a single nodule < 2 cm and 2–3 nodules < 3 cm [24].

As for the DFS, nine of them favored surgical resection in terms of DFS/RFS at any time points [11, 27, 31, 61, 72, 89, 97, 106, 155]; three showed that the 1-year DFS was statistically similar between the two groups, but the 3- and/or 5-year DFS were better in surgical resection group than in RFA group [16, 33, 151]; one reported that surgical resection had better DFS than RFA in the subgroup analyses of a single nodule 3–5 cm and ≤ 3 cm, but the DFS was statistically similar between the two groups in the subgroup analyses of a single nodule < 2 cm and 2–3 nodules < 3 cm [24].

As for the recurrence, three of them favored surgical resection [11, 47, 61]; two favored RFA [127, 151]; one found that the recurrence was statistically similar between the two groups [24]; three showed that the 1-year recurrence was statistically similar between the two groups, but the 3-year recurrence was less in surgical resection group than in RFA group [31, 33, 117]; one reported that the 1- and 3-year recurrence were statistically similar between the two groups, but the recurrence at the end of follow-up was less in RFA group than in surgical resection group [69]; one demonstrated that the distant intrahepatic recurrence was statistically similar between the two groups, but the local intrahepatic recurrence was less in surgical resection group than in RFA group [153]; one favored surgical resection in term of recurrence at previous sites, but favored RFA in term of recurrence at new sites [72].

According to the description of each meta-analysis, RCT studies were included the meta-analyses by Liu (Surg Laparosc Endosc Percutan Tech, 2010) (*n* = 8), Ni (*n* = 6), Fu (*n* = 5), Zhou (Zhonghua Wai Ke Za Zhi, 2011) (*n* = 4), Hu (*n* = 4), Feng (*n* = 3), Qi (*n* = 3), Wang (*n* = 3), Weis (*n* = 3), Duan (*n* = 2), Li (*n* = 2), Sun (*n* = 2), Xu (*n* = 2), Chen (*n* = 1), and Zhou (BMC Gastroenterol, 2010) (*n* = 1). The information regarding the inclusion of RCT studies was not available in the meta-analyses by Cai and Cucchetti. After checking the relevant information, we confirmed the following: 1) in the meta-analysis by Liu (Surg Laparosc Endosc Percutan Tech, 2010), all included studies were non-RCTs; 2) in the meta-analysis by Ni, there were one RCT regarding resection v.s. PEI, one RCT regarding resection v.s. PEI or microwave ablation, and two RCTs with overlapped data; 3) in the meta-analysis by Fu, there were one RCT regarding comparison of resection v.s. PEI or microwave ablation and two RCTs with overlapped data; 4) in the meta-analysis by Zhou (Zhonghua Wai Ke Za Zhi, 2011), one included study was non-RCT; and 5) because no included studies were listed in the meta-analysis by Hu, we could not check the accuracy. Thus, the largest number of RCT studies included in the meta-analyses should be 3.

The meta-analysis by Wang had the largest number of included studies (*n* = 28) followed by the meta-analyses by Feng (*n* = 23), Hu (*n* = 18), Cucchetti (*n* = 17), Xu (*n* = 13), Duan (*n* = 12), Sun (*n* = 11), Zhou (BMC Gastroenterol, 2010) (*n* = 10), Ni (*n* = 10), Liu (World J Gastroenterol, 2010) (*n* = 10), Liu (Surg Laparosc Endosc Percutan Tech, 2010) (*n* = 8), Li (*n* = 6), Chen (*n* = 6), Fu (*n* = 5), Cai (*n* = 5), Zhou (Zhonghua Wai Ke Za Zhi, 2011) (*n* = 4), Weis (*n* = 3), and Qi (*n* = 3) (Supplementary Table S21).

Given the superiority in the quantity of RCT studies, the meta-analyses by Feng, Qi, Wang, and Weis might be more reliable. In details, surgical resection should be superior to RFA for the improvement of OS.

### PEI versus surgical resection

Two meta-analyses compared the outcomes of PEI versus surgical resection [46, 100]. Both of them demonstrated that OS and RFS were statistically similar between the two groups [46, 100].

Only one RCT study was included in the meta-analysis by Schoppmeyer.

The meta-analysis by Hoshida had a larger number of included studies than that by Schoppmeyer (5 versus 1) (Supplementary Table S22). However, no included studies were overlapped between them.

The results were completely consistent between the two meta-analyses. In details, PEI was similar to surgical resection in terms of OS and RFS.

### Non-surgical-resection ablation versus surgical resection

One meta-analysis compared the outcomes of non-surgical-resection ablation versus surgical resection [26]. The 1- and 3-year survival and DFS were statistically similar between the two groups [26].

### RFA versus PEI or PAI

Eight meta-analyses compared the outcomes of RFA versus PEI or PAI [9, 20, 26, 38, 93, 102, 119, 130]. All of them favored RFA over PEI in terms of OS, DFS, and/or recurrence [9, 20, 26, 38, 93, 102, 119, 130]. Additionally, one of them found that the OS, local recurrence, de novo tumor, and adverse event were statistically similar between RFA and PAI groups [38].

RCT studies were included in the meta-analyses by Bouza (*n* = 6), Weis (*n* = 6), Xu (*n* = 6), Dong (*n* = 5), Germani (*n* = 5), Orlando (*n* = 5), Cho (*n* = 4), and Shen (*n* = 4).

The meta-analyses by Bouza, Weis, and Xu had the largest number of included studies (*n* = 6) followed by the meta-analyses by Dong (*n* = 5), Germani (*n* = 5), Orlando (*n* = 5), Shen (*n* = 5), and Cho (*n* = 4) (Supplementary Table S23).

The results regarding the comparison between RFA v.s. PEI were completely consistent among meta-analyses. In details, RFA should be superior to PEI for the improvement of OS and DFS.

### RFA versus cryosurgery ablation

One meta-analysis compared the outcomes of RFA versus cryosurgery ablation [51]. Although the OS was statistically similar between the two groups, RFA had less recurrence and complications than cryosurgery ablation [51].

### RFA versus other therapeutic methods

One meta-analysis compared the outcomes of RFA versus any other therapeutic methods [50]. RFA was superior to other treatment methods for early HCC in terms of local recurrence and 3-year survival [50]. However, no subgroup analysis was performed according to the different treatment modalities.

Additionally, one meta-analysis compared the outcomes of RFA versus microwave or laser ablation [119]. However, only one trial was identified for each comparison.

### PEI versus PAI

Two meta-analyses compared the outcomes of PEI versus PAI [38, 100]. Both of them showed that the OS, RFS, and recurrence were statistically similar between the two groups [38, 100].

RCT studies were included in the meta-analyses by Germani (*n* = 2) and Schoppmeyer (*n* = 2).

Both of them had a similar number of included studies (*n* = 2) (Supplementary Table S24). However, not all of the included studies were identical.

The results were completely consistent between the two meta-analyses. In details, PEI was similar to PAI in terms of OS and RFS.

### RFA + TACE versus mono-therapy

Eleven meta-analyses compared the outcomes of RFA in combination with TACE versus RFA or TACE alone [26, 45, 53, 55, 68, 71, 74, 87–88, 133, 144]. Seven of them favored the combination therapy in term of OS [45, 53, 55, 71, 88, 133, 144]; two favored the combination therapy in terms of 1- and 3-year survival, but not 5-year survival [74, 87]; one favored the combination therapy in term of 5-year survival, but not 1- or 3-year survival [26]; one found that the 1-year survival was statistically similar between the two groups [68].

Three meta-analyses compared the RFS of RFA in combination with TACE versus RFA or TACE alone [71, 87–88]. As for the 1-year RFS, one meta-analysis favored the combination therapy [71], but another two showed that the 1-year RFS was statistically similar between the two groups [87–88]. By comparison, all of them favored the combination therapy in term of 3-year RFS [71, 87–88].

RCT studies were included in the meta-analyses by Zhao (*n* = 21), Jiang (*n* = 8), Kong (*n* = 8), Ni (World J Gastroenterol, 2013) (*n* = 8), Yan (*n* = 8), Liu (*n* = 7), Lu (*n* = 7), Ni (J Cancer Res Clin Oncol, 2013) (*n* = 6), and Liao (*n* = 1), but not in the meta-analyses by Dong and Han.

The meta-analysis by Zhao had the largest number of included studies (*n* = 21), followed by those by Jiang (*n* = 19), Kong (*n* = 19), Yan (*n* = 19), Yan (*n* = 18), Han (*n* = 8), Ni (World J Gastroenterol, 2013) (*n* = 8), Liu (*n* = 7), Lu (*n* = 7), Ni (J Cancer Res Clin Oncol, 2013) (*n* = 6), Dong (*n* = 5), and Liao (*n* = 1) (Supplementary Table S25). Notably, all of the 19 included studies were completely identical among the three meta-analyses by Jiang, Kong, and Yan.

Given the superiority in the number of RCTs, the meta-analyses by Zhao, Jiang, Kong, Ni, and Yan should be more reliable. In details, RFA in combination with TACE should be favored in term of OS.

### PEI + TACE versus mono-therapy

Three meta-analyses compared the outcomes of PEI in combination with TACE versus PEI or TACE alone [68, 114–115]. Two of them favored the combination therapy in term of OS [114–115]. Another one meta-analysis was performed according to the study design. In the subgroup analysis of RCTs, the combination therapy significantly improved the 3-year survival, rather than 1-year survival. By contrast, in the subgroup analysis of observational studies, the combination therapy significantly improved the 1-year survival, rather than 3-year survival [68].

RCT studies were included in all of the 3 meta-analyses by Wang N (Med Oncol, 2011) (*n* = 7), Wang W (Liver Int, 2010) (*n* = 6), and Liao (PLoS One, 2013) (*n* = 4).

The meta-analysis by Wang N (Med Oncol, 2011) had a larger number of included studies than those by Wang W (Liver Int, 2010) and Liao (7 versus 6 and 4) (Supplementary Table S26). However, not all studies included by Wang W and Liao were included by Wang N.

Given the superiority in the number of RCTs, the meta-analysis by Wang N (Med Oncol, 2011) might be more reliable. In details, PEI in combination with TACE should be favored in term of OS.

### Any ablation therapy + TACE versus mono-therapy

Two meta-analyses compared the outcomes of unclassified ablation therapies in combination with TACE versus mono-therapy [42, 115]. Both of them favored the combination therapy in terms of OS, recurrence, and tumor response [42, 115].

RCT studies were included in the meta-analyses by Wang (*n* = 10) and Gu (*n* = 7).

The meta-analysis by Gu had a larger number of included studies than that by Wang (18 versus 10) (Supplementary Table S27). However, not all studies included by Wang were included by Gu.

The results were completely consistent between the two meta-analyses. In details, TACE in combination with ablation therapy was favored.

## TACE

### TACE/TAE versus no active treatment

Seven meta-analyses compared the outcomes of TACE/TAE versus no active treatment or supportive care [12, 39, 57, 73, 77, 91, 132]. Two of them showed that the OS was statistically similar between the two groups [39, 91]; another five favored TACE/TAE in term of OS [12, 57, 73, 77, 132].

RCT studies were included in the meta-analyses by Marelli (*n* = 9), Oliveri (*n* = 8), Llovet (*n* = 7), Camma (*n* = 5), Geschwind (*n* = 4), and Leng (*n* = 2), but not in the meta-analysis by Xue.

The meta-analysis by Marelli had the largest number of included studies (*n* = 9) followed by those by Oliveri (*n* = 8), Xue (*n* = 8), Llovet (*n* = 7), Camma (*n* = 5), Geschwind (*n* = 4), and Leng (*n* = 3) (Supplementary Table S28). However, not all included studies were completely overlapped among them.

Given the superiority in the number of RCTs, the meta-analysis by Marelli might be more reliable. In details, TACE/TAE should be favored.

### TACE versus TAE

Three meta-analyses compared the outcomes of TACE versus TAE [12, 77, 125]. All of them showed that the OS was statistically similar between the two groups [12, 77, 125].

Only RCT studies were included in the meta-analyses by Xie (*n* = 5), Marelli (*n* = 3), and Camma (*n* = 2).

The meta-analysis by Xie had a larger number of included studies than those by Marelli and Camma (5 versus 3 and 2) (Supplementary Table S29). However, not all included studies were completely overlapped among them.

The results were completely consistent among them. In details, TACE was similar to TAE in term of OS.

### Drug-eluting bead (DEB)-TACE versus conventional TACE (cTACE)

Three meta-analyses compared the outcomes of DEB-TACE versus cTACE [37, 44, 48]. One of them evaluated the OS [48]. DEB-TACE was significantly better than cTACE in terms of 1- and 2-year survival. But the 6-month and 3-year survival were statistically similar between the two groups.

Two of them demonstrated that tumor response or disease control rate was statistically similar between them. Another one meta-analysis demonstrated that tumor response rate was significantly higher in DEB-TACE group than in cTACE group.

Two of them evaluated the complications [37, 44]. The incidence of complications was statistically similar between the two groups.

RCT studies were included in the meta-analyses by Han (*n* = 3) and Huang (*n* = 2), but not in the meta-analysis by Gao.

The meta-analysis by Huang had a larger number of included studies than those by Han and Gao (7 versus 5 and 2) (Supplementary Table S30). However, not all included studies were completely overlapped among them.

Given the superiority in the number of RCTs, the meta-analysis by Han might be more reliable. In details, DEB-TACE was similar to cTACE in the term of tumor response.

### TACE versus microsphere embolization

One meta-analysis compared the outcomes of TACE versus microsphere embolization [123]. Microsphere embolization was superior to TACE in terms of OS, TTP, and tumor response [123]. In the subgroup analyses, the benefit was statistically significant in patients undergoing ^32^P glass microspheres, but not in those undergoing ^90^Y microspheres.

RCT studies were included in the meta-analysis by Xie (*n* = 7).

### TACE + sorafenib versus TACE

Four meta-analyses compared the outcomes of TACE in combination with sorafenib versus TACE alone [35, 70, 134, 140]. Three of them favored the combination therapy in term of OS [35, 134, 140], but another one found that the OS was statistically similar between the two groups [70]. The survival benefit of the combination therapy was statistically significant in the subgroup analysis of retrospective studies, but not in that of RCTs [134].

RCT studies were included in the meta-analyses by Liu (*n* = 3), Yang (*n* = 3), and Zhang (*n* = 2), but not in the meta-analysis by Fu.

The meta-analysis by Fu had the largest number of included studies (*n* = 9), followed by those by Liu (*n* = 7), Yang (*n* = 6), and Zhang (*n* = 6) (Supplementary Table S31). However, not all included studies were completely overlapped among the 4 meta-analyses.

Given the superiority in the number of RCTs, the meta-analysis by Liu and Yang might be more reliable. In details, TACE plus sorafenib was not favored in term of OS.

### TACE + high-intensity focused ultrasound (HIFU) versus TACE

Two meta-analyses compared the outcomes of TACE in combination with HIFU versus TACE alone [13, 68]. One of them demonstrated that both OS and tumor response were improved by the combination therapy [13]. Another one meta-analysis was performed according to the study design [68]. In the subgroup analysis of observational studies, both 1- and 3-year survival were significantly improved by the combination therapy [68]. By comparison, in the subgroup analysis of RCTs, only 1-year survival, rather than 3-year survival, was significantly improved by the combination therapy [68].

RCT study was included in the meta-analysis by Liao (*n* = 1), but not in the meta-analysis by Cao.

The meta-analysis by Cao had a larger number of included studies than that by Liao (9 versus 5) (Supplementary Table S32). All studies which were included in the meta-analysis by Liao were also included in the meta-analysis by Cao.

Given the superiority in the number of RCTs, the meta-analysis by Liao might be more reliable. In details, TACE plus HIFU should be favored in term of 1-year survival, but not 3-year survival.

### TACE + thermotherapy versus TACE

Only one meta-analysis compared the outcomes of TACE in combination with thermotherapy versus TACE alone [64]. Both 1- and 2-year survival were significantly improved by the combination therapy, but the 0.5-, 1.5-, and 3-year survival were statistically similar between the two groups [64]. Additionally, the overall effective rate and quality of life were improved by the combination therapy [64].

### TACE + AHCS versus TACE or AHCS

Only one meta-analysis compared the outcomes of TACE in combination with AHCS versus TACE or AHCS alone [65]. Compared with TACE alone, the combination therapy had significantly better 0.5-, 1-, 1.5-, 2-, and 2.5-year survival, but the 3-year survival was statistically similar between the two groups [65]. Compared with AHCS alone, the combination therapy had significantly better 0.5-, 1.5-, 2-, and 2.5-year survival, but similar 1- and 3-year survival [65]. Additionally, the combination therapy was superior to the mono-therapy in terms of total effective rate, complete necrosis rate, recurrence, AFP reduction, and CD4 improvement.

### TACE + radiotherapy versus TACE alone

Two meta-analyses compared the outcomes of TACE in combination with radiotherapy versus TACE alone [68, 80]. Both of them demonstrated that the combination therapy had significantly better 1-, 2-, 3-, and 5-year survival than TACE alone [68, 80]. Additionally, one of them showed that the combination therapy significantly increased the tumor response, but did not influence the development of adverse events, such as nausea/vomit, leukocyte count declined, alanine aminotransferase level increased, and total bilirubin level increased [80].

RCT studies were included in the meta-analyses by Meng (*n* = 5) and Liao (*n* = 3).

The meta-analysis by Meng had a larger number of included studies than that by Liao (17 versus 7) (Supplementary Table S33). All studies which were included in the meta-analysis by Liao were also included in the meta-analysis by Meng.

The results regarding the OS were completely consistent among them. In details, TACE plus radiotherapy should be favored in term of OS.

### TACE + 3D-CRT versus TACE alone

Only one meta-analysis compared the outcomes of TACE in combination with 3D-CRT versus TACE alone [68]. Regardless of study design, the combination therapy was superior to TACE alone in terms of 1- and 3-year survival [68].

Only one RCT study was included in the meta-analysis by Liao.

### TACE + TCMs versus TACE alone

Six meta-analyses compared the outcomes of TACE in combination with TCMs versus TACE alone [18–19, 79, 81, 108, 122]. Three of them favored the combination therapy in term of OS [18, 79, 108]; one favored the combination therapy in terms of 1-, 2-, and 3-year survival, but not 6-month survival [19]; one favored the combination therapy in term of 2-year survival, but not 1-year survival [122]; one did not report the survival data [81].

Five of them favored the combination therapy in term of tumor response [18–19, 79, 81, 122]. Another one did not report the relevant data [108].

Four of them favored the combination therapy in term of quality of life [18–19, 79, 108]. Another two did not report the relevant data [81, 122].

RCT studies were included in the meta-analyses by Cheung (*n* = 67), Cho (*n* = 30), and Meng (Explore (NY), 2011) (*n* = 11), but not in the meta-analyses by Sun and Wu. The information regarding the inclusion of RCTs was not reported in the meta-analysis by Meng (J Altern Complement Med, 2008).

The meta-analysis by Cheung had the largest number of included studies (*n* = 67), followed by those by Meng (*n* = 37), Cho (*n* = 30), Meng (*n* = 12), Sun (*n* = 10), and Wu (*n* = 9) (Supplementary Table S34). However, not all included studies were completely overlapped among the 6 meta-analyses.

Given the superiority in the number of RCTs, the meta-analysis by Cheung might be more reliable. In details, TACE plus TCMs should be favored in terms of OS, tumor response, and quality of life.

### TACE + CIK cell therapy versus TACE alone

Two meta-analyses compared the outcomes of TACE in combination with CIK cell therapy versus TACE alone [14, 63]. The combination therapy was beneficial in terms of OS, RFS, TTP, quality of life, and liver and immune function [14, 63]. Additionally, one of them evaluated whether or not adjunctive CIK cell therapy could improve the outcomes of TACE in combination with RFA [63]. Adjunctive CIK cell therapy was beneficial in terms of OS and RFS [63].

RCT studies were included in the meta-analyses by Chen (*n* = 9) and Li (*n* = 6).

The meta-analysis by Li had a larger number of included studies than that by Chen (11 versus 9) (Supplementary Table S35). However, not all included studies were completely overlapped between them.

The results regarding the OS were completely consistent among them. In details, TACE in combination with CIK cell therapy should be favored.

### Sorafenib

Seven meta-analyses compared the outcomes of sorafenib versus placebo (Supplementary Table S1) [22, 28, 101, 118, 141, 143, 160]. The use of sorafenib was beneficial in terms of OS, TTP, and disease control rate [22, 101, 118, 141, 143, 160]. However, the time to symptomatic progression was statistically similar between the two groups [22, 118, 160]. The incidence of adverse events was significantly increased by the use of sorafenib [28, 101, 118, 141, 143, 160].

RCT studies were included in the meta-analyses by Shen (*n* = 5), Duffy (*n* = 4), Wang (*n* = 4), Zhang T (Anticancer Drugs, 2010) (*n* = 3), Zhang X (Hepatobiliary Pancreat Dis Int, 2012) (*n* = 3), Cinco (*n* = 2), and Zou (*n* = 2).

The meta-analysis by Shen had a larger number of included studies than those by Wang, Duffy, Zhang T (Anticancer Drugs, 2010), Zhang X (Hepatobiliary Pancreat Dis Int, 2012), Zou, and Cinco (5 versus 4, 4, 3, 3, 2, and 2) (Supplementary Table S36). All studies which were included in the meta-analysis by Wang, Duffy, Zhang T (Anticancer Drugs, 2010), Zhang X (Hepatobiliary Pancreat Dis Int, 2012), and Zou were also included by Shen. In the meta-analysis by Cinco, the included studies were not reported.

The results were completely consistent among them. In details, sorafenib should be favored.

### Antiviral therapy

Nineteen meta-analyses compared the outcomes of antiviral therapy versus no antiviral therapy (Supplementary Table S2) [10, 49, 54, 56, 58, 84–86, 103, 105, 107, 112, 120, 128, 139, 142, 154, 158–159]. Thirteen of them favored the use of antiviral therapy in term of OS [10, 49, 54, 84, 86, 105, 107, 112, 120, 128, 139, 142, 154]; one found that the use of antiviral therapy significantly improved the 5-year survival in HCV patients, but not HBV patients [58]; one demonstrated that the 1-year survival was statistically similar between the two groups [56]; one showed that 1-, 2-, 3-, 4-, and 5-year survival were statistically similar between the two groups [159]; another three did not report the survival data [85, 103, 158].

Five meta-analyses evaluated the DFS/RFS [49, 56, 103, 112, 154]. Four of them favored the use of antiviral therapy in term of DFS/RFS [56, 103, 112, 154]. Another one meta-analysis was performed according to the study design and type of viral hepatitis. In the subgroup analysis of RCTs, the DFS/RFS was statistically similar between the two groups regardless of HCV or HBV [49]. In the subgroup analysis of non-RCTs, antiviral therapy improved the DFS/RFS by in HCV patients, but not HBV patients [49].

Fifteen meta-analyses evaluated the recurrence [10, 54, 56, 58, 84–85, 105, 107, 120, 128, 139, 142, 154, 158–159]. Ten of them favored the use of antiviral therapy in term of recurrence [10, 56, 84–85, 105, 107, 120, 139, 154, 158]; one favored the use of antiviral therapy after TACE, but not surgical resection [54]; two favored the use of antiviral therapy in HCV patients, but not HBV patients [58, 128]; one favored the use of antiviral therapy in terms of 1-, 3-, and 4-year recurrence, but not 2- or 5-year recurrence [159]; one favored the use of antiviral therapy in terms of 1- and 2-year recurrence, but not late recurrence (> 2 year) [142].

RCT studies were included in the meta-analyses by Zhuang (PLoS One, 2013) (*n* = 13), Huang (*n* = 10), Shen (*n* = 9), Wang (*n* = 9), Zhang (Mol Clin Oncol, 2014) (*n* = 9), Jiang (*n* = 8), Li (*n* = 8), Zhuang (Zhonghua Gan Zang Bing Za Zhi, 2012) (*n* = 8), Breitenstein (*n* = 7), Zhang (Int J Cancer, 2009) (*n* = 6), Miao (*n* = 5), Singal (*n* = 5), Xu (*n* = 5), Moriguchi (*n* = 4), Miyake (*n* = 3), Sun (*n* = 1), and Zhou (*n* = 1), rather than those by Lan and Wong.

The meta-analysis by Huang had a larger number of included studies than those by Zhang, Zhou, Miao, Shen, Sun, Zhuang (PLoS One, 2013), Jiang, Lan, Miyake, Singal, Wang, Wong, Xu, Li, Zhang, Zhuang (Zhonghua Gan Zang Bing Za Zhi, 2012), Breitenstein, and Moriguchi (23 versus 19, 14, 13, 13, 13, 13, 10, 10, 10, 10, 9, 9, 9, 8, 8, 8, 7, and 4) (Supplementary Table S37). In the meta-analysis by Moriguchi, the included studies were not reported. However, not all included studies were completely overlapped between them.

Given the superiority in the number of RCTs, the meta-analysis by Zhuang (PLoS One, 2013) and Huang might be more reliable. In details, interferon therapy after curative treatment should be favored.

### Vitamin

Five meta-analyses compared the outcomes of vitamin versus placebo (Supplementary Table S3) [21, 82, 99, 112, 150]. Two of them favored the use of vitamin in term of OS [112, 150]; two favored the use of vitamin in term of 2-year survival, but not 3-year survival [21, 82]; one showed that the 1- and 2-year survival were statistically similar between the two groups [99].

One meta-analysis favored the use of vitamin in term of RFS [112].

Two meta-analyses favored the use of vitamin in term of 1-year recurrence, but another two did not [21, 82]. Four meta-analyses favored the use of vitamin in terms of 2- and 3-year recurrence [21, 82].

RCT studies were included in the meta-analyses by Wang (*n* = 6), Zhong (*n* = 6), Chu (*n* = 5), Riaz (*n* = 5), and Meng (*n* = 4).

The meta-analysis by Zhong had a larger number of included studies than those by Chu, Wang, Riaz, and Meng (7 versus 6, 6, 5, and 4) (Supplementary Table S38). In the meta-analysis by Meng, the included studies were not reported. However, not all included studies were completely overlapped between them.

Given the superiority in the number of RCTs, the meta-analyses by Wang and Zhong should be more reliable. In details, the use of vitamin should be favored in term of OS. However, its benefit was weak.

### Octreotide

Three meta-analyses compared the outcomes of octreotide versus placebo (Supplementary Table S4) [29, 43, 52]. As for the 6- and 12-month survival, one of them favored the use of octreotide [52], but another two did not show any significant difference between the two groups [29, 43]. As for the 24-month survival, two of them showed that the survival was statistically similar between the two groups [29, 43], but another one did not report the relevant data [52].

RCT studies were included in the meta-analyses by Ji (*n* = 9) and Guo (*n* = 6).

The meta-analysis by Ji had a larger number of included studies than those by Guo and Estanislao (11 versus 6 and 3) (Supplementary Table S39). In the meta-analysis by Estanislao, the included studies were not reported. All studies which were included in the meta-analysis by Guo were also included by Ji.

Given the superiority in the number of RCTs, the meta-analyses by Ji and Guo might be more reliable. In details, the benefit of octreotide remains uncertain.

### TCM

One meta-analysis compared the outcomes of kanglaite injection plus hepatic arterial intervention versus hepatic arterial intervention alone (Supplementary Table S5) [34]. The combination therapy was beneficial in terms of tumor response, Karnofsky score improvement, and pain relief [34]. But neither OS nor DFS/RFS was evaluated [34].

One meta-analysis compared the outcomes of Chinese herbal medicine plus chemotherapy versus chemotherapy alone [104]. The combination therapy was beneficial in terms of OS and tumor response [104].

One meta-analysis compared the outcomes of TCM versus other treatment [121]. TCM was superior to other treatments in terms of OS and tumor response [121].

### CIK cell therapy

One meta-analysis compared the outcomes of CIK cell therapy versus other treatment (Supplementary Table S6) [76]. CIK cell therapy was superior to other treatments in terms of OS, PFS, disease control rate, tumor response, and quality of life [76].

### Tamoxifen

Two meta-analyses compared the outcomes of tamoxifen versus placebo or no treatment (Supplementary Table S7) [73, 90]. Both of them demonstrated that the OS was statistically similar between the two groups [73, 90].

RCT studies were included in the meta-analyses by Nowak (*n* = 10) and Llovet (*n* = 7).

Although the meta-analysis by Nowak had a larger number of included studies than that by Llovet (10 versus 7) (Supplementary Table S40), the included studies were not similar between them.

The results were completely consistent among them. In details, tamoxifen should not be favored.

### Antibiotics

One meta-analysis compared the outcomes of antibiotics versus no antibiotics after hepatic transarterial therapy [113]. The incidence of fever, bacteremia, septicema, and sepsis were not significantly improved by antibiotics [113].

## DISCUSSION

AASLD and EASL guidelines recommend BCLC staging algorithm for the management of HCC. Only 5 treatment modalities have been considered in the current guidelines. In details, the therapeutic modalities of HCC include the LT, surgical resection, and RFA for HCC in the stage 0 and A, TACE for HCC in the stage B, sorafenib for HCC in the stage C, and supportive treatment for HCC in the stage D. However, the BCLC staging algorithm is not flawless and needs to be persistently updated. Nowadays, more and more novel treatment modalities have been widely produced and adopted. Their efficacy and safety have been gradually established. In this circumstance, our study was worthwhile, because it attempted to collect the relevant evidence as many as possible and to provide an overview of outcomes of novel and well-established treatment modalities for HCC based on the results of meta-analyses. More notably, we found that lots of combination therapy might be more effective and safe. For example, the meta-analyses of RCTs demonstrated that RFA plus TACE was superior to mono-therapy, and that surgical resection plus post-operative TACE was superior to surgical resection alone. Given the quality of such meta-analyses, the guidelines should be updated regarding the use of combination therapy.

### Limitations

This was a time-consuming work, because a large number of relevant meta-analyses were included. Several limitations should be acknowledged. First, we must clarify that only the results of meta-analyses, but not the accuracy of meta-analyses, were systematically reviewed. Because we cannot repeat every meta-analysis, we cannot guarantee that their findings were accurate. Second, we did not consider the heterogeneity among included studies in every meta-analysis. A significant heterogeneity could affect the stability of a meta-analysis. Third, we arbitrarily evaluated the reliability of meta-analyses according to the number of RCTs and non-RCTs.

### Recommendations

#### LT

1. LDLT has lower DFS than DDLT (*grade of recommendation: low*).
2. Short- and long-term outcomes may be comparable between primary and salvage LT (*grade of recommendation: low*).
3. Sirolimus-based immunosuppression should be recommended after LT (*grade of recommendation: low*).

#### Surgical resection

1. Surgical resection margin aiming at 2 cm may be superior to 1 cm for the improvement of long-term outcomes (*grade of recommendation: moderate*).
2. Survival benefit may be comparable between laparoscopic and open resection. Additionally, laparoscopic resection had less blood loss, blood transfusion, and complications and shorter hospital stay (*grade of recommendation: low*).
3. Anatomic resection, but not non-anatomic resection, should be recommended (*grade of recommendation: low*).
4. Adjunctive I^131^ lipiodol therapy may be considered in patients undergoing surgical resection (*grade of recommendation: low*).
5. Post-operative TACE, but not pre-operative TACE, may be considered in patients undergoing surgical resection (*grade of recommendation: high*).
6. Immunotherapy may not be considered in patients undergoing surgical resection (*grade of recommendation: high*).
7. PVE may not be considered in patients undergoing surgical resection (*grade of recommendation: low*).

#### Ablation

1. Surgical resection should be superior to RFA in term of OS (*grade of recommendation: high*).
2. RFA, but not PEI or cryosurgery ablation, should be recommended (*grade of recommendation: high*).
3. RFA in combination with TACE may be superior to TACE or RFA mono-therapy (*grade of recommendation: high*).
4. PEI in combination with TACE may be superior to TACE or PEI mono-therapy (*grade of recommendation: high*).

#### TAE/TACE

1. TACE/TAE should be superior to placebo (*grade of recommendation: high*).
2. Survival benefit may be comparable between TACE and TAE (*grade of recommendation: high*).
3. DEB-TACE was comparable to conventional TACE (*grade of recommendation: high*).
4. ^32^P glass microspheres embolization may be superior to TACE for the improvement of OS (*grade of recommendation: high*).
5. Adjunctive HIFU therapy may further improve the outcomes of TACE (*grade of recommendation: moderate*).
6. Adjunctive radiotherapy therapy may further improve the outcomes of TACE (*grade of recommendation: high*).
7. Adjunctive 3D-CRT therapy may further improve the outcomes of TACE (*grade of recommendation: moderate*).
8. Adjunctive TCMs therapy may further improve the outcomes of TACE (*grade of recommendation: high*).
9. Adjunctive CIK cell therapy may further improve the outcomes of TACE (*grade of recommendation: high*).

#### Sorafenib

1. Sorafenib is superior to placebo for the improvement of OS (*grade of recommendation: high*).

## Other treatments

1. Antiviral therapy should be recommended for the improvement of recurrence (*grade of recommendation: high*).
2. Vitamin should be recommended for the improvement of OS (*grade of recommendation: high*).
3. Tamoxifen should not be recommended (*grade of recommendation: high*).

### Uncertainties

1. The superiority of LT to surgical resection for the improvement of OS remains inconclusive. The accurate candidates for LT and surgical resection need to be clearly established.
2. The superiority of surgical resection to RFA for the improvement of OS remains under debate. The indications of RFA should be refined.
3. Although transarterial radioembolization appears to be more advantageous than TACE, their cost-effectiveness should be further explored.
4. Although TACE appears to be more effective than no treatment, the survival benefit of TAE/TACE versus other active treatments should be confirmed.
5. The benefits of combination therapy may be confirmed in the future guidelines.

## MATERIALS AND METHODS

### Search strategy and study selection

We searched all meta-analysis papers regarding the treatment of HCC via the PubMed, EMBASE, and Cochrane library databases. Search items were as follows: (hepatocellular carcinoma) AND (meta-analysis). The last search was performed on October 1, 2014.

Eligibility criteria were as follows. 1) All meta-analyses regarding the treatment of HCC were included. 2) Duplicate publications were excluded. 3) Narrative reviews were excluded. 4) Only systematic reviews without meta-analyses were excluded. 5) Only systematic review protocols were excluded. 6) Patients without HCC were excluded. 7) Other topics, but not treatment modalities, were excluded.

Primary outcomes were overall survival (OS), diseases-free survival (DFS) or recurrence-free survival (RFS), progression or time-to-progression (TTP), progression-free survival (PFS), recurrence or time-to-recurrence, safety, and other endpoints.

### Reliability of meta-analyses

As the results were different among the meta-analyses, the reliability was evaluated according to the quality and quantity of original studies included in every meta-analysis. First, we evaluated the quality of original studies. If a larger number of randomized controlled trials (RCTs) were included, the results of a meta-analysis would be more reliable. Second, if the number of randomized controlled trials was similar, we further evaluated the number of non-RCT studies. If a larger number of non-RCT studies were included, the results of a meta-analysis would be more reliable. Third, if the number of RCT and non-RCT studies included was similar but the results were different among meta-analyses, we further evaluated the statistical methods. Hazard ratio could reflect a general effect over time; by comparison, odds ratio or risk ratio reflected an individual effect at a fixed time point. Thus, if the hazard ratio was calculated, the results of a meta-analysis would be more reliable.

### Grade of recommendations

High grade recommendation was considered, if the results of meta-analyses were based on more than 3 single-center RCTs or 1 multi-center RCT. Low grade recommendation was considered, if the results of meta-analyses were based on the non-RCT studies alone. As for something in between, moderate grade recommendation was considered.

## SUPPLEMENTARY MATERIAL FIGURES AND TABLES



























## Abbreviations

3D-CRT: three-dimensional conformal radiation therapy
AHCS: argon-helium cryotherapy system
CIK: cytokine-induced killer
DDLT: deceased donor liver transplantation
DEB: drug-eluting bead
DFS: diseases-free survival
HCC: hepatocellular carcinoma
HIFU: high-intensity focused ultrasound
LDLT: living donor liver transplantation
LT: liver transplantation
OS: overall survival
PEI: percutaneous ethanol injection
PAI: percutaneous acetic acid injection
PFS: progression-free survival
PVE: portal vein embolization
RCT: randomized controlled trials
RFA: radiofrequency ablation
RFS: recurrence-free survival
TACE: transarterial chemoembolization
TAE: transarterial embolization
TCMs: traditional Chinese medicine
TTP: time-to-progression.

## References

[1] Forner A, Llovet JM, Bruix J (2012). Hepatocellular carcinoma. Lancet.

[2] El-Serag HB (2011). Hepatocellular carcinoma. N Engl J Med.

[3] Wallace MC, Preen D, Jeffrey GP, Adams LA (2015). The evolving epidemiology of hepatocellular carcinoma: a global perspective. Expert Rev Gastroenterol Hepatol.

[4] Bruix J, Sherman M (2011). Management of hepatocellular carcinoma: an update. Hepatology.

[5] (2012). EASL-EORTC clinical practice guidelines: management of hepatocellular carcinoma. J Hepatol.

[6] Cook DJ, Mulrow CD, Haynes RB (1997). Systematic reviews: synthesis of best evidence for clinical decisions. Ann Intern Med.

[7] (2009). Oxford Centre for Evidence-based Medicine – Levels of Evidence.

[8] Al Hasan I, Emond JC (2014). Liver trasplantation for HCC, does the type of donor graft really matter? Survival and recurrence meta-anlysis. Liver Transplantation.

[9] Bouza C, Lopez-Cuadrado T, Alcazar R, Saz-Parkinson Z, Amate JM (2009). Meta-analysis of percutaneous radiofrequency ablation versus ethanol injection in hepatocellular carcinoma. BMC Gastroenterol.

[10] Breitenstein S, Dimitroulis D, Petrowsky H, Puhan MA, Mullhaupt B, Clavien PA (2009). Systematic review and meta-analysis of interferon after curative treatment of hepatocellular carcinoma in patients with viral hepatitis. Br J Surg.

[11] Cai H, Zhou T, Qiu YD (2013). Comparison of radiofrequency ablation and surgical resection in patients with solitary hepatocellular carcinoma within 5 cm: A me. HPB.

[12] Camma C, Schepis F, Orlando A, Albanese M, Shahied L, Trevisani F, Andreone P, Craxi A, Cottone M (2002). Transarterial chemoembolization for unresectable hepatocellular carcinoma: meta-analysis of randomized controlled trials. Radiology.

[13] Cao H, Xu Z, Long H, Zhang LL, Zhang J, Peng ZP, Li SL (2011). Transcatheter arterial chemoembolization in combination with high-intensity focused ultrasound for unresectable hepatocellular carcinoma: a systematic review and meta-analysis of the chinese literature. Ultrasound Med Biol.

[14] Chen B, Xu X, Xiang M, Yang J, Yu T, Hu Y (2013). Cytokine-induced killer cell combination with TACE in the treatment of hepatocellular cancers: A meta-analysis. Chinese-German Journal of Clinical Oncology.

[15] Chen J, Huang K, Wu J, Zhu H, Shi Y, Wang Y, Zhao G (2011). Survival after anatomic resection versus nonanatomic resection for hepatocellular carcinoma: a meta-analysis. Dig Dis Sci.

[16] Chen RF, Xiao TH, Zhou QB (2008). Therapeutic clinical effect of radiofrequency ablation for small hepatocellular carcinoma in cirrhotic patients: a meta-analysis. [Article in Chinese] Zhonghua Wai Ke Za Zhi.

[17] Cheng X, Sun P, Hu QG, Song ZF, Xiong J, Zheng QC (2014). Transarterial (chemo)embolization for curative resection of hepatocellular carcinoma: a systematic review and meta-analyses. J Cancer Res Clin Oncol.

[18] Cheung F, Wang X, Wang N, Yuen MF, Ziea TC, Tong Y, Wong VT, Feng Y (2013). Chinese Medicines as an Adjuvant Therapy for Unresectable Hepatocellular Carcinoma during Transarterial Chemoembolization: A Meta-Analysis of Randomized Controlled Trials. Evid Based Complement Alternat Med.

[19] Cho WC, Chen HY (2009). Transcatheter arterial chemoembolization combined with or without Chinese herbal therapy for hepatocellular carcinoma: meta-analysis. Expert Opin Investig Drugs.

[20] Cho YK, Kim JK, Kim MY, Rhim H, Han JK (2009). Systematic review of randomized trials for hepatocellular carcinoma treated with percutaneous ablation therapies. Hepatology.

[21] Chu KJ, Lai EC, Yao XP, Zhang HW, Lau WY, Fu XH, Lu CD, Shi J, Cheng SQ (2010). Vitamin analogues in chemoprevention of hepatocellular carcinoma after resection or ablation—a systematic review and meta-analysis. Asian J Surg.

[22] Cinco RUC, Ignacio JG, Domingo F (2011). Sorafenib in the treatment of advanced hepatocellular carcinoma: A meta-analysis of randomized trials. Hepatology International.

[23] Cucchetti A, Cescon M, Ercolani G, Bigonzi E, Torzilli G, Pinna AD (2012). A comprehensive meta-regression analysis on outcome of anatomic resection versus nonanatomic resection for hepatocellular carcinoma. Ann Surg Oncol.

[24] Cucchetti A, Piscaglia F, Cescon M, Colecchia A, Ercolani G, Bolondi L, Pinna AD (2013). Cost-effectiveness of hepatic resection versus percutaneous radiofrequency ablation for early hepatocellular carcinoma. J Hepatol.

[25] Dhir M, Lyden ER, Smith LM, Are C (2012). Comparison of outcomes of transplantation and resection in patients with early hepatocellular carcinoma: a meta-analysis. HPB (Oxford).

[26] Dong W, Zhang T, Wang ZG, Liu H (2014). Clinical outcome of small hepatocellular carcinoma after different treatments: a meta-analysis. World J Gastroenterol.

[27] Duan C, Liu M, Zhang Z, Ma K, Bie P (2013). Radiofrequency ablation versus hepatic resection for the treatment of early-stage hepatocellular carcinoma meeting Milan criteria: a systematic review and meta-analysis. World J Surg Oncol.

[28] Duffy A, Wilkerson J, Greten TF (2013). Hemorrhagic events in hepatocellular carcinoma patients treated with antiangiogenic therapies. Hepatology.

[29] Estanislao NI, Ang E, Flores H, Salvana A, Tripon E, Velasquez ME, Ong JP (2009). Octreotide for improving survival in patients with advanced hepatocellular carcinoma: A meta-analysis. Journal of Gastroenterology and Hepatology.

[30] Fancellu A, Rosman AS, Sanna V, Nigri GR, Zorcolo L, Pisano M, Melis M (2011). Meta-analysis of trials comparing minimally-invasive and open liver resections for hepatocellular carcinoma. J Surg Res.

[31] Feng Q, Chi Y, Liu Y, Zhang L, Liu Q (2014). Efficacy and safety of percutaneous radiofrequency ablation versus surgical resection for small hepatocellular carcinoma: a meta-analysis of 23 studies. J Cancer Res Clin Oncol.

[32] Flores HF, Ang ER, Estanislao INI (2009). Adjuvant immunotherapy in patients with hepatocellular carcinoma who underwent curative resection: A meta-analysis. Journal of Gastroenterology and Hepatology.

[33] Fu C, Liu N, Deng Q, Li X, Ma K, Bie P (2014). Radiofrequency ablation vs. surgical resection on the treatment of patients with small hepatocellular carcinoma: A system review and meta-analysis of five randomized controlled trials. Hepato-Gastroenterology.

[34] Fu F, Wan Y, Wu T (2014). Kanglaite injection combined with hepatic arterial intervention for unresectable hepatocellular carcinoma: A meta-analysis. J Cancer Res Ther.

[35] Fu QH, Zhang Q, Bai XL, Hu QD, Su W, Chen YW, Su RG, Liang TB (2014). Sorafenib enhances effects of transarterial chemoembolization for hepatocellular carcinoma: a systematic review and meta-analysis. J Cancer Res Clin Oncol.

[36] Furtado R, Crawford M, Sandroussi C (2014). Systematic review and meta-analysis of adjuvant i lipiodol after excision of hepatocellular carcinoma. Ann Surg Oncol.

[37] Gao S, Yang Z, Zheng Z, Yao J, Deng M, Xie H, Zheng S, Zhou L (2013). Doxorubicin-eluting bead versus conventional TACE for unresectable hepatocellular carcinoma: a meta-analysis. Hepatogastroenterology.

[38] Germani G, Pleguezuelo M, Gurusamy K, Meyer T, Isgro G, Burroughs AK (2010). Clinical outcomes of radiofrequency ablation, percutaneous alcohol and acetic acid injection for hepatocelullar carcinoma: a meta-analysis. J Hepatol.

[39] Geschwind JF, Ramsey DE, Choti MA, Thuluvath PJ, Huncharek MS (2003). Chemoembolization of hepatocellular carcinoma: results of a metaanalysis. Am J Clin Oncol.

[40] Gong L, Shi L, Sun J, Yuan WS, Chen JF, Liu P, Gong F, Dong JH (2014). Comparative survival analysis of adjuvant therapy with iodine-131-labeled lipiodol to hepatic resection of primary hepatocellular carcinoma: a meta-analysis. Nucl Med Commun.

[41] Grant RC, Sandhu L, Dixon PR, Greig PD, Grant DR, McGilvray ID (2013). Living vs. deceased donor liver transplantation for hepatocellular carcinoma: a systematic review and meta-analysis. Clin Transplant.

[42] Gu L, Liu H, Fan L, Lv Y, Cui Z, Luo Y, Liu Y, Li G, Li C, Ma J (2014). Treatment outcomes of transcatheter arterial chemoembolization combined with local ablative therapy versus monotherapy in hepatocellular carcinoma: a meta-analysis. J Cancer Res Clin Oncol.

[43] Guo TK, Hao XY, Ma B, Yang KH, Li YP, Li HL, Gu YH, Cai H, Liu YL, Li Y, Zhan WP (2009). Octreotide for advanced hepatocellular carcinoma: a meta-analysis of randomized controlled trials. J Cancer Res Clin Oncol.

[44] Han S, Zhang X, Zou L, Lu C, Zhang J, Li J, Li M (2014). Does drug-eluting bead transcatheter arterial chemoembolization improve the management of patients with hepatocellular carcinoma? A meta-analysis. PLoS One.

[45] Han X, Lv WF (2013). Transcatheter arterial chemoembolization combined with radiofrequency ablation for the treatment of hepatocellular carcinoma: A meta-analysis of long-term efficacy. Journal of Interventional Radiology (China).

[46] Hoshida Y, Shiratori Y, Omata M (2000). Difficulties in conducting controlled trials in radical therapies for nonadvanced hepatocellular carcinoma. Hepatology.

[47] Hu P, Zhang SJ, Sun AX, Qian GJ (2013). Meta-analysis of survival and disease recurrence for small hepatocellular carcinoma after radiofrequency ablation and surgical resection. HPB.

[48] Huang K, Zhou Q, Wang R, Cheng D, Ma Y (2014). Doxorubicin-eluting beads versus conventional transarterial chemoembolization for the treatment of hepatocellular carcinoma. J Gastroenterol Hepatol.

[49] Huang TS, Shyu YC, Chen HY, Yuan SS, Shih JN, Chen PJ (2013). A systematic review and meta-analysis of adjuvant interferon therapy after curative treatment for patients with viral hepatitis-related hepatocellular carcinoma. J Viral Hepat.

[50] Huang X, Lu B, Meng LN (2008). A meta-analysis of radiofrequency ablation for early hepatocellular carcinoma. [Article in Chinese] Zhonghua Nei Ke Za Zhi.

[51] Huang YZ, Zhou SC, Zhou H, Tong M (2013). Radiofrequency ablation versus cryosurgery ablation for hepatocellular carcinoma: a meta-analysis. Hepatogastroenterology.

[52] Ji XQ, Ruan XJ, Chen H, Chen G, Li SY, Yu B (2011). Somatostatin analogues in advanced hepatocellular carcinoma: an updated systematic review and meta-analysis of randomized controlled trials. Med Sci Monit.

[53] Jiang G, Xu X, Ren S, Wang L (2014). Combining transarterial chemoembolization with radiofrequency ablation for hepatocellular carcinoma. Tumour Biol.

[54] Jiang S, Liu Y, Wang L, Duan C, Liu M (2013). A meta-analysis and systematic review: adjuvant interferon therapy for patients with viral hepatitis-related hepatocellular carcinoma. World J Surg Oncol.

[55] Kong QF, Jiao JB, Chen QQ, Li L, Wang DG, Lv B (2014). Comparative effectiveness of radiofrequency ablation with or without transarterial chemoembolization for hepatocellular carcinoma. Tumour Biol.

[56] Lan X, Liu HM, Zeng W, Gu HG, Yang JT, Xie B, Tang C, Chen P (2013). Effects of lamivudine on outcome after initial treatment for hepatocellular carcinoma: A meta-analysis. Journal of Gastroenterology and Hepatology (Hong Kong).

[57] Leng JJ, Xu YZ, Dong JH (2014). Efficacy of transarterial chemoembolization for hepatocellular carcinoma with portal vein thrombosis: a meta-analysis. ANZ J Surg.

[58] Li CZ, Liu JY, Zhang ZM (2013). Adjuvant interferon therapy for hepatocellular carcinoma after curative treatment: A meta-analysis of randomized controlled trials. Chinese Journal of Cancer Prevention and Treatment.

[59] Li HY, Wei YG, Yan LN, Li B (2012). Salvage liver transplantation in the treatment of hepatocellular carcinoma: a meta-analysis. World J Gastroenterol.

[60] Li JD, Tian YH, Kanduri HK, Li QL, Li DX, Li Q (2011). Prognosis in patients with small hepatocellular carcinoma: A meta-analysis. Hepato-Gastroenterology.

[61] Li L, Zhang J, Liu X, Li X, Jiao B, Kang T (2012). Clinical outcomes of radiofrequency ablation and surgical resection for small hepatocellular carcinoma: a meta-analysis. J Gastroenterol Hepatol.

[62] Li N, Wu YR, Wu B, Lu MQ (2012). Surgical and oncologic outcomes following laparoscopic versus open liver resection for hepatocellular carcinoma: A meta-analysis. Hepatol Res.

[63] Li X, Dai D, Song X, Liu J, Zhu L, Xu W (2014). A meta-analysis of cytokine-induced killer cells therapy in combination with minimally invasive treatment for hepatocellular carcinoma. Clin Res Hepatol Gastroenterol.

[64] Li Z, Mi D, Yang K, Cao N, Tian J, Ma B (2012). TACE combined with thermotherapy for primary hepatic carcinoma: A meta-analysis. Chinese Journal of Evidence-Based Medicine.

[65] Li Z, Mi DH, Yang KH, Cao N, Tian JH, Ma B, Liu YL (2013). Effectiveness and safety of TACE combined with AHCS for primary hepatic carcinomas: A systematic review. Chinese Journal of Evidence-Based Medicine.

[66] Liang W, Wang D, Ling X, Kao AA, Kong Y, Shang Y, Guo Z, He X (2012). Sirolimus-based immunosuppression in liver transplantation for hepatocellular carcinoma: a meta-analysis. Liver Transpl.

[67] Liang W, Wu L, Ling X, Schroder PM, Ju W, Wang D, Shang Y, Kong Y, Guo Z, He X (2012). Living donor liver transplantation versus deceased donor liver transplantation for hepatocellular carcinoma: a meta-analysis. Liver Transpl.

[68] Liao M, Huang J, Zhang T, Wu H (2013). Transarterial chemoembolization in combination with local therapies for hepatocellular carcinoma: a meta-analysis. PLoS One.

[69] Liu JG, Wang YJ, Du Z (2010). Radiofrequency ablation in the treatment of small hepatocellular carcinoma: a meta analysis. World J Gastroenterol.

[70] Liu L, Chen H, Wang M, Zhao Y, Cai G, Qi X, Han G (2014). Combination therapy of sorafenib and TACE for unresectable HCC: a systematic review and meta-analysis. PLoS One.

[71] Liu Z, Gao F, Yang G, Singh S, Lu M, Zhang T, Zhong Z, Zhang F, Tang R (2014). Combination of radiofrequency ablation with transarterial chemoembolization for hepatocellular carcinoma: an up-to-date meta-analysis. Tumour Biol.

[72] Liu Z, Zhou Y, Zhang P, Qin H (2010). Meta-analysis of the therapeutic effect of hepatectomy versus radiofrequency ablation for the treatment of hepatocellular carcinoma. Surg Laparosc Endosc Percutan Tech.

[73] Llovet JM, Bruix J (2003). Systematic review of randomized trials for unresectable hepatocellular carcinoma: Chemoembolization improves survival. Hepatology.

[74] Lu Z, Wen F, Guo Q, Liang H, Mao X, Sun H (2013). Radiofrequency ablation plus chemoembolization versus radiofrequency ablation alone for hepatocellular carcinoma: a meta-analysis of randomized-controlled trials. Eur J Gastroenterol Hepatol.

[75] Ma L, Zhong JH, Li LQ (2011). Postoperative adjuvant adoptive immunotherapy for hepatocellular carcinoma: A Meta-analysis. Chinese Journal of Cancer Prevention and Treatment.

[76] Ma Y, Xu YC, Tang L, Zhang Z, Wang J, Wang HX (2012). Cytokine-induced killer (CIK) cell therapy for patients with hepatocellular carcinoma: efficacy and safety. Experimental Hematology and Oncology.

[77] Marelli L, Stigliano R, Triantos C, Senzolo M, Cholongitas E, Davies N, Tibballs J, Meyer T, Patch DW, Burroughs AK (2007). Transarterial therapy for hepatocellular carcinoma: which technique is more effective? A systematic review of cohort and randomized studies. Cardiovasc Intervent Radiol.

[78] Mathurin P, Raynard B, Dharancy S, Kirzin S, Fallik D, Pruvot FR, Roumilhac D, Canva V, Paris JC, Chaput JC, Naveau S (2003). Meta-analysis: evaluation of adjuvant therapy after curative liver resection for hepatocellular carcinoma. Aliment Pharmacol Ther.

[79] Meng MB, Cui YL, Guan YS, Ying Z, Zheng MH, Yuan CK, Zhang RM (2008). Traditional Chinese medicine plus transcatheter arterial chemoembolization for unresectable hepatocellular carcinoma. J Altern Complement Med.

[80] Meng MB, Cui YL, Lu Y, She B, Chen Y, Guan YS, Zhang RM (2009). Transcatheter arterial chemoembolization in combination with radiotherapy for unresectable hepatocellular carcinoma: a systematic review and meta-analysis. Radiother Oncol.

[81] Meng MB, Wen QL, Cui YL, She B, Zhang RM (2011). Meta-analysis: traditional Chinese medicine for improving immune response in patients with unresectable hepatocellular carcinoma after transcatheter arterial chemoembolization. Explore (NY).

[82] Meng W, Li Y, Li X, Zhou W, Zhu X, He W, Liu Y (2011). Can vitamin K2 be applied to prevent the recurrence of hepatocellular carcinoma solely?. Hepatology International.

[83] Menon KV, Hakeem AR, Heaton ND (2013). Meta-analysis: recurrence and survival following the use of sirolimus in liver transplantation for hepatocellular carcinoma. Aliment Pharmacol Ther.

[84] Miao RY, Zhao HT, Yang HY, Mao YL, Lu X, Zhao Y, Liu CN, Zhong SX, Sang XT, Huang JF (2010). Postoperative adjuvant antiviral therapy for hepatitis B/C virus-related hepatocellular carcinoma: a meta-analysis. World J Gastroenterol.

[85] Miyake Y, Takaki A, Iwasaki Y, Yamamoto K (2010). Meta-analysis: interferon-alpha prevents the recurrence after curative treatment of hepatitis C virus-related hepatocellular carcinoma. J Viral Hepat.

[86] Moriguchi H, Uemura T, Sato C, Raymond C (2006). Tumor ablation plus interferon therapy can reduce the risk of death for patients with hepatitis B virus or hepatitis C virus-related resectable hepatocellular carcinoma - a meta-analysis of randomized controlled trials. Hepatology.

[87] Ni JY, Liu SS, Xu LF, Sun HL, Chen YT (2013). Meta-analysis of radiofrequency ablation in combination with transarterial chemoembolization for hepatocellular carcinoma. World J Gastroenterol.

[88] Ni JY, Liu SS, Xu LF, Sun HL, Chen YT (2013). Transarterial chemoembolization combined with percutaneous radiofrequency ablation versus TACE and PRFA monotherapy in the treatment for hepatocellular carcinoma: a meta-analysis. J Cancer Res Clin Oncol.

[89] Ni JY, Xu LF, Sun HL, Zhou JX, Chen YT, Luo JH (2013). Percutaneous ablation therapy versus surgical resection in the treatment for early-stage hepatocellular carcinoma: a meta-analysis of 21,494 patients. J Cancer Res Clin Oncol.

[90] Nowak A, Findlay M, Culjak G, Stockler M (2004). Tamoxifen for hepatocellular carcinoma. Cochrane Database Syst Rev.

[91] Oliveri RS, Wetterslev J, Gluud C (2011). Transarterial (chemo)embolisation for unresectable hepatocellular carcinoma. Cochrane Database Syst Rev.

[92] Ono T, Yamanoi A, Nazmy El Assal O, Kohno H, Nagasue N (2001). Adjuvant chemotherapy after resection of hepatocellular carcinoma causes deterioration of long-term prognosis in cirrhotic patients: metaanalysis of three randomized controlled trials. Cancer.

[93] Orlando A, Leandro G, Olivo M, Andriulli A, Cottone M (2009). Radiofrequency thermal ablation vs. percutaneous ethanol injection for small hepatocellular carcinoma in cirrhosis: meta-analysis of randomized controlled trials. Am J Gastroenterol.

[94] Pang SJ, Xu J, Dong ZY (2010). Laparoscopic hepatectomy compared with conventional open hepatectomy for hepatocellular carcinoma: A systematic review. Chinese Journal of Evidence-Based Medicine.

[95] Parks KR, Kuo YH, Davis JM, O'Brien B, Hagopian EJ (2014). Laparoscopic versus open liver resection: a meta-analysis of long-term outcome. HPB (Oxford).

[96] Proneth A, Zeman F, Schlitt HJ, Schnitzbauer AA (2014). Is resection or transplantation the ideal treatment in patients with hepatocellular carcinoma in cirrhosis if both are possible? A systematic review and metaanalysis. Annals of Surgical Oncology.

[97] Qi X, Tang Y, An D, Bai M, Shi X, Wang J, Han G, Fan D (2014). Radiofrequency ablation versus hepatic resection for small hepatocellular carcinoma: a meta-analysis of randomized controlled trials. J Clin Gastroenterol.

[98] Rahman A, Assifi MM, Pedroso FE, Maley WR, Sola JE, Lavu H, Winter JM, Yeo CJ, Koniaris LG (2012). Is resection equivalent to transplantation for early cirrhotic patients with hepatocellular carcinoma? A meta-analysis. J Gastrointest Surg.

[99] Riaz IB, Riaz H, Riaz T, Rahman S, Amir M, Badshah MB, Kazi AN (2012). Role of vitamin K2 in preventing the recurrence of hepatocellular carcinoma after curative treatment: a meta-analysis of randomized controlled trials. BMC Gastroenterol.

[100] Schoppmeyer K, Weis S, Mossner J, Fleig WE (2009). Percutaneous ethanol injection or percutaneous acetic acid injection for early hepatocellular carcinoma. Cochrane Database Syst Rev.

[101] Shen A, Tang C, Wang Y, Chen Y, Yan X, Zhang C, Liu R, Wei X, Zhu Y, Zhang H, Wu Z (2013). A systematic review of sorafenib in Child-Pugh A patients with unresectable hepatocellular carcinoma. J Clin Gastroenterol.

[102] Shen A, Zhang H, Tang C, Chen Y, Wang Y, Zhang C, Wu Z (2013). Systematic review of radiofrequency ablation versus percutaneous ethanol injection for small hepatocellular carcinoma up to 3 cm. J Gastroenterol Hepatol.

[103] Shen YC, Hsu C, Chen LT, Cheng CC, Hu FC, Cheng AL (2010). Adjuvant interferon therapy after curative therapy for hepatocellular carcinoma (HCC): a meta-regression approach. J Hepatol.

[104] Shu X, McCulloch M, Xiao H, Broffman M, Gao J (2005). Chinese herbal medicine and chemotherapy in the treatment of hepatocellular carcinoma: a meta-analysis of randomized controlled trials. Integr Cancer Ther.

[105] Singal AK, Freeman DH, Anand BS (2010). Meta-analysis: interferon improves outcomes following ablation or resection of hepatocellular carcinoma. Aliment Pharmacol Ther.

[106] Sun B, Zheng CS, Feng GS, Wang Y, Xia XW, Kan XF (2011). Radiofrequency ablation versus surgical resection for small hepatocellular carcinoma: A meta-analysis. World Chinese Journal of Digestology.

[107] Sun P, Dong X, Cheng X, Hu Q, Zheng Q (2014). Nucleot(s)ide analogues for hepatitis B virus-related hepatocellular carcinoma after curative treatment: a systematic review and meta-analysis. PLoS One.

[108] Sun Q, Ma W, Gao Y, Zheng W, Zhang B, Peng Y (2012). Meta-analysis: therapeutic effect of transcatheter arterial chemoembolization combined with compound kushen injection in hepatocellular carcinoma. Afr J Tradit Complement Altern Med.

[109] Tang YH, Wen TF, Chen X (2012). Resection margin in hepatectomy for hepatocellular carcinoma: a systematic review. Hepatogastroenterology.

[110] Tang YH, Wen TF, Chen X (2013). Anatomic versus non-anatomic liver resection for hepatocellular carcinoma: a systematic review. Hepatogastroenterology.

[111] Twaij A, Pucher PH, Sodergren MH, Gall T, Darzi A, Jiao LR (2014). Laparoscopic vs open approach to resection of hepatocellular carcinoma in patients with known cirrhosis: systematic review and meta-analysis. World J Gastroenterol.

[112] Wang J, He XD, Yao N, Liang WJ, Zhang YC (2013). A meta-analysis of adjuvant therapy after potentially curative treatment for hepatocellular carcinoma. Can J Gastroenterol.

[113] Wang J, He XD, Zhang YC (2012). Antibiotic prophylaxis in transarterial therapy of hepatocellular carcinoma: a meta-analysis. Can J Gastroenterol.

[114] Wang N, Guan Q, Wang K, Zhu B, Yuan W, Zhao P, Wang X, Zhao Y (2011). TACE combined with PEI versus TACE alone in the treatment of HCC: a meta-analysis. Med Oncol.

[115] Wang W, Shi J, Xie WF (2010). Transarterial chemoembolization in combination with percutaneous ablation therapy in unresectable hepatocellular carcinoma: a meta-analysis. Liver Int.

[116] Wang X, Li J, Peng Y, Dai Y, Xu W (2011). Influence of preoperative transarterial chemoembolization on the prognosis for patients with resectable hepatocellular carcinoma: a meta-analysis of randomized trials. Hepatogastroenterology.

[117] Wang Y, Luo Q, Li Y, Deng S, Wei S, Li X (2014). Radiofrequency ablation versus hepatic resection for small hepatocellular carcinomas: a meta-analysis of randomized and nonrandomized controlled trials. PLoS One.

[118] Wang Z, Wu XL, Zeng WZ, Xu GS, Xu H, Weng M, Hou JN, Jiang MD (2013). Meta-analysis of the efficacy of sorafenib for hepatocellular carcinoma. Asian Pac J Cancer Prev.

[119] Weis S, Franke A, Mossner J, Jakobsen JC, Schoppmeyer K (2013). Radiofrequency (thermal) ablation versus no intervention or other interventions for hepatocellular carcinoma. Cochrane Database Syst Rev.

[120] Wong JS, Wong GL, Tsoi KK, Wong VW, Cheung SY, Chong CN, Wong J, Lee KF, Lai PB, Chan HL (2011). Meta-analysis: the efficacy of anti-viral therapy in prevention of recurrence after curative treatment of chronic hepatitis B-related hepatocellular carcinoma. Aliment Pharmacol Ther.

[121] Wu P, Dugoua JJ, Eyawo O, Mills EJ (2009). Traditional Chinese Medicines in the treatment of hepatocellular cancers: a systematic review and meta-analysis. J Exp Clin Cancer Res.

[122] Wu T, Sun R, Wang Z, Yang W, Shen S, Zhao Z (2014). A meta-analysis of Cinobufacini combined with transcatheterarterial chemoembolization in the treatment of advanced hepatocellular carcinoma. J Cancer Res Ther.

[123] Xie F, Zang J, Guo X, Xu F, Shen R, Yan L, Yang J, He J (2012). Comparison of transcatheter arterial chemoembolization and microsphere embolization for treatment of unresectable hepatocellular carcinoma: a meta-analysis. J Cancer Res Clin Oncol.

[124] Xie F, Zhang X, Li H, Zheng T, Xu F, Shen R, Yan L, Yang J, He J (2012). Adoptive immunotherapy in postoperative hepatocellular carcinoma: a systemic review. PLoS One.

[125] Xie ZB, Ma L, Wang XB, Bai T, Ye JZ, Zhong JH, Li LQ (2014). Transarterial embolization with or without chemotherapy for advanced hepatocellular carcinoma: a systematic review. Tumour Biol.

[126] Xiong JJ, Altaf K, Javed MA, Huang W, Mukherjee R, Mai G, Sutton R, Liu XB, Hu WM (2012). Meta-analysis of laparoscopic vs open liver resection for hepatocellular carcinoma. World J Gastroenterol.

[127] Xu G, Qi FZ, Zhang JH, Cheng GF, Cai Y, Miao Y (2012). Meta-analysis of surgical resection and radiofrequency ablation for early hepatocellular carcinoma. World J Surg Oncol.

[128] Xu JB, Qi FZ, Xu G, Chen GF, Huang MD, Zhang JH (2014). Adjuvant interferon therapy after surgical treatment for hepatitis B/C virus-related hepatocellular carcinoma: A meta-analysis. Hepatol Res.

[129] Xu QR, Zheng X, Yao YM, Yang W, Liu QG (2012). Liver transplantation versus resection for treatment of hepatocellular carcinoma: A meta-analysis. Journal of Xi'an Jiaotong University (Medical Sciences).

[130] Xu RH, Gao W, Wang C, Guo DK, Tang L, Zhang H, Wang CJ (2014). Systematic evaluation of percutaneous radiofrequency ablation versus percutaneous ethanol injection for the treatment of small hepatocellular carcinoma: a meta-analysis. Eur J Med Res.

[131] Xu XS, Liu C, Qu K, Song YZ, Zhang P, Zhang YL (2014). Liver transplantation versus liver resection for hepatocellular carcinoma: a meta-analysis. Hepatobiliary Pancreat Dis Int.

[132] Xue TC, Xie XY, Zhang L, Yin X, Zhang BH, Ren ZG (2013). Transarterial chemoembolization for hepatocellular carcinoma with portal vein tumor thrombus: a meta-analysis. BMC Gastroenterol.

[133] Yan S, Xu D, Sun B (2012). Combination of radiofrequency ablation with transarterial chemoembolization for hepatocellular carcinoma: a meta-analysis. Dig Dis Sci.

[134] Yang M, Yuan JQ, Bai M, Han GH (2014). Transarterial chemoembolization combined with sorafenib for unresectable hepatocellular carcinoma: a systematic review and meta-analysis. Mol Biol Rep.

[135] Yao G, Cao J, Gu H, Zhang Jh, Han AYFHA, Cao XL, Wen H (2013). Laparoscopic hepatectomy versus open hepatectomy for hepatocellular carcinoma: A meta-analysis. Chinese Journal of Evidence-Based Medicine.

[136] Ye JZ, Miao ZG, Wu FX, Zhao YN, Ye HH, Li LQ (2012). Recurrence after anatomic resection versus nonanatomic resection for hepatocellular carcinoma: a meta-analysis. Asian Pac J Cancer Prev.

[137] Yin Z, Fan X, Ye H, Yin D, Wang J (2013). Short- and long-term outcomes after laparoscopic and open hepatectomy for hepatocellular carcinoma: a global systematic review and meta-analysis. Ann Surg Oncol.

[138] Yu T, Xu X, Chen B (2013). TACE combined with liver resection versus liver resection alone in the treatment of resectable HCC: A meta-analysis. Chinese-German Journal of Clinical Oncology.

[139] Zhang CH, Xu GL, Jia WD, Ge YS (2009). Effects of interferon alpha treatment on recurrence and survival after complete resection or ablation of hepatocellular carcinoma: a meta-analysis of randomized controlled trials. Int J Cancer.

[140] Zhang L, Hu P, Chen X, Bie P (2014). Transarterial chemoembolization (TACE) plus sorafenib versus TACE for intermediate or advanced stage hepatocellular carcinoma: a meta-analysis. PLoS One.

[141] Zhang T, Ding X, Wei D, Cheng P, Su X, Liu H, Wang D, Gao H (2010). Sorafenib improves the survival of patients with advanced hepatocellular carcinoma: a meta-analysis of randomized trials. Anticancer Drugs.

[142] Zhang W, Song TQ, Zhang T, Wu Q, Kong DL, Li Q, Sun HC (2014). Adjuvant interferon for early or late recurrence of hepatocellular carcinoma and mortality from hepatocellular carcinoma following curative treatment: A meta-analysis with comparison of different types of hepatitis. Molecular and Clinical Oncology.

[143] Zhang X, Yang XR, Huang XW, Wang WM, Shi RY, Xu Y, Wang Z, Qiu SJ, Fan J, Zhou J (2012). Sorafenib in treatment of patients with advanced hepatocellular carcinoma: a systematic review. Hepatobiliary Pancreat Dis Int.

[144] Zhao S, Chen XC, Long QY, Zhang XL (2013). Transcatheter arterial chemoembolization combined with radiofrequency ablation for the treatment of hepatocellular carcinoma: A systematic review and meta analysis. Journal of Interventional Radiology (China).

[145] Zhao WY, Luo M, Sun YW, Xu Q, Chen W, Zhao G, Wu ZY (2008). The efficacy of preoperative portal vein embolization for extended hepatectomy: a meta-analysis. [Article in Chinese] Zhonghua Wai Ke Za Zhi.

[146] Zheng Z, Liang W, Milgrom DP, Schroder PM, Kong NS, Yang C, Guo Z, He X (2014). Liver transplantation versus liver resection in the treatment of hepatocellular carcinoma: a meta-analysis of observational studies. Transplantation.

[147] Zheng Z, Liang W, Wang D, Schroder PM, Ju W, Wu L, Shang Y, Guo Z, He X (2014). Adjuvant chemotherapy for patients with primary hepatocellular carcinoma: A meta-analysis. Int J Cancer.

[148] Zhong J, Xiang B, Ma L, Li L (2014). Conventional oral systemic chemotherapy for postoperative hepatocellular carcinoma: A systematic review. Molecular and Clinical Oncology.

[149] Zhong JH, Li LQ (2010). Postoperative adjuvant transarterial chemoembolization for participants with hepatocellular carcinoma: A meta-analysis. Hepatol Res.

[150] Zhong JH, Li LQ, You XM, Xiang BD, Ma L, Gong WF (2012). Oral vitamin K2 analogs for hepatocellular carcinoma after surgery: A meta-analysis. World Chinese Journal of Digestology.

[151] Zhou DC, Geng XP, Zhu LX, Zhao HC, Liu FB, Zhao YJ (2011). Percutaneous radiofrequency ablation versus hepatic resection for small hepatocellular carcinoma: a meta analysis. [Article in Chinese] Zhonghua Wai Ke Za Zhi.

[152] Zhou Y, Xu D, Wu L, Li B (2011). Meta-analysis of anatomic resection versus nonanatomic resection for hepatocellular carcinoma. Langenbecks Arch Surg.

[153] Zhou Y, Zhang X, Wu L, Ye F, Su X, Shi L, Li B (2013). Meta-analysis: preoperative transcatheter arterial chemoembolization does not improve prognosis of patients with resectable hepatocellular carcinoma. BMC Gastroenterol.

[154] Zhou Y, Zhang Z, Zhao Y, Wu L, Li B (2014). Antiviral therapy decreases recurrence of hepatitis B virus-related hepatocellular carcinoma after curative resection: a meta-analysis. World J Surg.

[155] Zhou Y, Zhang X, Wu L, Ye F, Su X, Shi L, Li B (2010). Meta-analysis of radiofrequency ablation versus hepatic resection for small hepatocellular carcinoma. BMC Gastroenterol.

[156] Zhou YM, Shao WY, Zhao YF, Xu DH, Li B (2011). Meta-analysis of laparoscopic versus open resection for hepatocellular carcinoma. Dig Dis Sci.

[157] Zhu Y, Dong J, Wang WL, Li MX, Lu Y (2013). Short- and long-term outcomes after salvage liver transplantation versus primary liver transplantation for hepatocellular carcinoma: a meta-analysis. Transplant Proc.

[158] Zhuang L, Zeng X, Yang Z, Meng Z (2013). Effect and safety of interferon for hepatocellular carcinoma: a systematic review and meta-analysis. PLoS One.

[159] Zhuang LP, Zeng XT, Meng ZQ (2012). A systematic review and meta-analysis of randomized controlled trails. [Article in Chinese] Zhonghua Gan Zang Bing Za Zhi.

[160] Zou LL, Ma L, Tian JH, Yang KH (2011). Sorafenib for advanced hepatocellular carcinoma: A systematic review. Acta Academiae Medicinae Sinicae.

