# Supplementary material for: Management of hepatocellular carcinoma: an overview of major findings from meta-analyses
Source: Oncotarget. 2016 May 4;7(23):34703–51. doi: 10.18632/oncotarget.9157 (PMC5085185; doi:10.18632/oncotarget.9157)
Supplement: Supplementary file 1 [file oncotarget-07-34703-s001.pdf]

Management of hepatocellular carcinoma: an overview of major findings from meta-analyses

Supplementary Materials

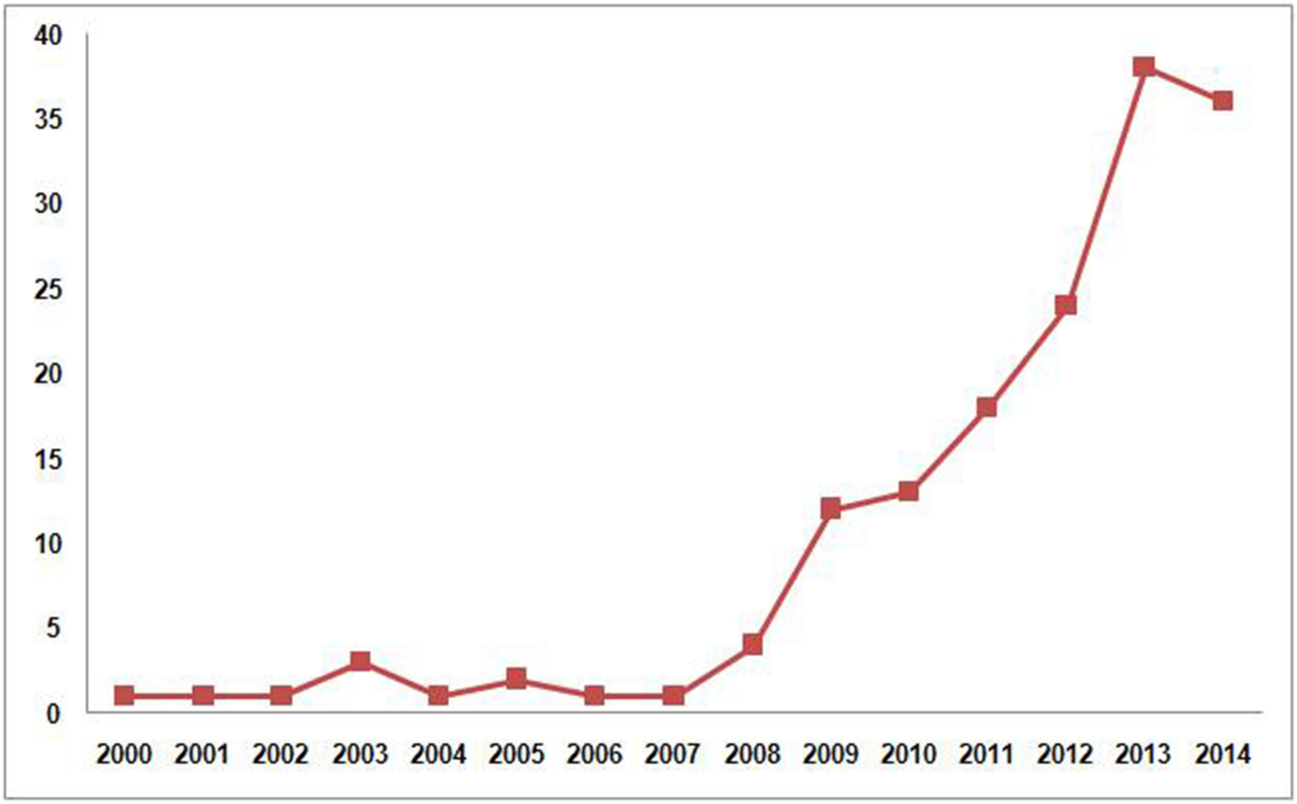

Supplementary Figure S1: Number of papers over years.

Supplementary Table S1: Findings of meta-analyses: An overview of included studies regarding sorafenib

First author	Journal (Year)	Comparisons	OS	DFS, RFS, TTP, PFS	Recurrence, time to recurrence	Other endpoints	Major comments
**Sorafenib**							
Cinco	Hepatol Int (2011)	Sorafenib vs placebo	Overall mortality: reduced in sorafenib.	Time to symptomatic progression: statistically similar.	NA.	NA.	Sorafenib showed improvement in overall mortality and also in extending survival time.
Duffy	Hepatology (2013)	Antiangiogenic agents vs placebo	NA.	NA.	NA.	Bleeding events: increased in sorafenib.	Sorafenib was associated with an increased risk of bleeding.
Shen	J Clin Gastroenterol (2013)	Sorafenib vs placebo	OS: favor sorafenib.	TTP: favor sorafenib.	NA.	Disease control rate: favor sorafenib. Overall adverse events: increased in sorafenib.	Sorafenib was a moderately effective and safe oral drug for use in Child-Pugh A patients with unresectable HCC. Sorafenib monotherapy is not recommended for treating intermediate-stage HCC.
Wang	Asian Pac J Cancer Prev (2013)	Sorafenib with or without chemotherapy vs placebo with or without chemotherapy	OS: favor sorafenib (systematic review but not meta-anlaysis).	TTP: favor sorafenib (systematic review but not meta-analysis). Time to symptomatic progression: statistically similar (systematic review but not meta-analysis).	NA.	Disease control rate: favor sorafenib. Hand-foot-skin reactions: higher in sorafenib. Hypodynamia: statistically similar. Diarrhea: higher in sorafenib. Hypertension: statistically similar. Skin rash or desquamation: higher in sorafenib.	Sorafenib exerts significant curative effects in HCC.
Zhang	Anticancer Drugs (2010)	Sorafenib-based therapy with other agent-based therapy	OS: favor sorafenib.	TTP: favor sorafenib.	NA.	Objective response rate: statistically similar. Hand-foot syndrome and diarrhea: higher in sorafenib. Other toxic events: statistically similar.	Sorafenib-based chemotherapy is superior to placebo-based chemotherapy in terms of TPP and OS without increase in severe toxic effects.
Zhang	Hepatobiliary Pancreat Dis Int (2012)	Sorafenib vs placebo	OS: favor sorafenib.	TTP: favor sorafenib.	NA.	Partial response rate: favor sorafenib. Most adverse events: higher in sorafenib.	Sorafenib-based therapy benefits advanced HCC patients.
Zou	Acta Academiae Medicinae Sinicae (2011)	Sorafenib vs placebo	OS: favor sorafenib (systematic review but not meta-anlaysis).	TTP: favor sorafenib (systematic review but not meta-analysis). Time to symptomatic progression: similar (systematic review but not meta-analysis).	NA.	Disease control rate: favor sorafenib. Fatigue: statistically similar. Alopecia: higher in sorafenib. Diarrhea: higher in sorafenib. Rash: statistically similar. Hand-foot-skin reactions: higher in sorafenib. Hypertension: statistically similar.	Sorafenib is effective and safe for the treatment of advanced HCC.

Supplementary Table S2: Findings of meta-analyses: An overview of included studies regarding antiviral therapy. See Supplementary_Table_S2

Supplementary Table S3: Findings of meta-analyses: An overview of included studies regarding vitamin

First author	Journal (Year)	Comparisons	OS	DFS, RFS, TTP, PFS	Recurrence, time to recurrence	Other endpoints	Major comments
**Vitamin**							
Chu	Asian J Surg (2010)	Vitamin analogues (vitamin A and K_2) after hepatic resection or local ablative therapy	OS:<br>2-year: favor vitamin K_2.<br>3-year: statistically similar.	NA.	Recurrence:<br>1-, 2-, 3-year: lower in vitamin K_2.	NA.	Chemopreventive therapy after partial hepatectomy or local ablative therapy is beneficial in prolonging DFS, but the evidence is less for an effect on the OS.
Meng	Hepatol Int (2011)	Vitamin K_2 vs placebo after curative treatment	OS:<br>2-year: favor vitamin K_2.<br>1-, 3-year: statistically similar.	NA.	Recurrence:<br>1-, 2-, 3-year: lower in vitamin K_2.	NA.	Administration of vitamin K_2 either reduces recurrence in 12, 24 and 36 months, or enhances survival in 24 months compared with the placebo after curative treatment of HCC.
Riaz	BMC Gastroenterol (2012)	Vitamin K_2 or its analogues vs placebo or no vitamin K	OS:<br>1-, 2-year: statistically similar.	NA.	Recurrence:<br>1-year: statistically similar.<br>2-, 3-year: lower in vitamin K_2.	NA.	It failed to confirm significantly better tumor recurrence-free survival at 1 year. Improved tumor recurrence at 2nd and 3rd year may be just due to insufficient data.
Wang	Can J Gastroenterol (2013)	Different adjuvant therapy after potentially curative treatment (including vitamin therapy)	OS:<br>Vitamin analogue therapy: favor vitamin analogue therapy.	RFS:<br>Vitamin analogue therapy: favor vitamin analogue therapy.	NA.	NA.	Vitamin analogue therapy produce limited success for survival.
Zhong	World Chinese J of Digestology (2012); PLoS One (2013)	Curative treatments alone vs curative treatments + oral vitamin K_2 analogs	OS:<br>1-, 2-, 3-year: favor vitamin K_2.	NA.	Recurrence:<br>1-year: statistically similar.<br>2-, 3-year: lower in vitamin K_2.	NA.	VK_2 analog therapy shows some benefit in reducing the recurrence rate and increasing OS in patients with HCC after hepatic resection or local ablation.

Supplementary Table S4: Findings of meta-analyses: An overview of included studies regarding octreotide

First author	Journal (Year)	Comparisons	OS	DFS, RFS, TTP, PFS	Recurrence, time to recurrence	Other endpoints	Major comments
**Octreotide**							
Estanislao	J Gastroenterol Hepatol (2009)	Octreotide vs no octreotide	OS: 6-, 12-, 24-month: statistically similar.	NA.	NA.	NA.	There is no significant difference with regards to OS among patients treated with octreotide and those who did not.
Guo	J Cancer Res Clin Oncol (2009)	Octreotide vs placebo or best supportive care	OS: 6-, 12-month: statistically similar (meta-analysis using random-effects model).	NA.	NA.	NA.	The result may not demonstrate a significant superiority of octreotide administration in participants with advanced HCC from the available evidence.
Ji	Med Sci Monit (2011)	Octreotide vs placebo or no treatment	OS: 6-, 12-month: favor octreotide. 24-month: statistically similar.	NA.	NA.	NA.	Octreotide could improve the survival of patients with advanced HCC, but possibly not in western countries.

Supplementary Table S5: Findings of meta-analyses: An overview of included studies regarding traditional Chinese medicine

First author	Journal (Year)	Comparisons	OS	DFS, RFS, TTP, PFS	Recurrence, time to recurrence	Other endpoints	Major comments
**Traditional Chinese medicine (TCM)**							
Fu	J Cancer Res Ther (2014)	Kanglaite injection plus hepatic arterial intervention vs hepatic arterial intervention alone	NA.	NA.	NA.	Objective response: favor Kanglaite injection. Karnofsky score improvement: favor Kanglaite injection. Pain relief: favor Kanglaite injection.	Kanglaite injection combined with hepatic arterial intervention can improve the short-term clinical efficacy, quality of life, and decrease the pain of patients with unresectable HCC.
Shu	Integr Cancer Ther (2005)	Chinese herbal medicine + chemotherapy vs chemotherapy alone	Survival: 12-, 24-, 36-month: favor TCMs.	NA.	NA.	Tumor response: favor TCMs.	Combining TCMs with chemotherapy may benefit patients with HCC.
Wu	J Exp Clin Cancer Res (2009)	TCMs vs other treatment	OS: 6-, 12-, 18-, 24-, 36-month: favor TCMs.	NA.	NA.	Complete response: favor TCMs. Partial response: favor TCMs. Stable disease: statistically similar. Abdominal pain and distension improvement: statistically similar. Fatigue and appetite improvement: favor TCMs.	Our meta-analysis displays compelling evidence of effectiveness of TCMs for HCC.

Supplementary Table S6: Findings of meta-analyses: An overview of included studies regarding cytokine-induced killer cell therapy

First author	Journal (Year)	Comparisons	OS	DFS, RFS, TTP, PFS	Recurrence, time to recurrence	Other endpoints	Major comments
**Cytokine-induced killer (CIK) cell therapy**							
Ma	Experimental Hematology and Oncology (2012)	CIK cell therapy vs non-CIK therapy	OS:<br>1-, 2-year: favor CIK cell therapy.	PFS:<br>0,5-, 1-year: favor CIK cell therapy.	NA.	Disease control rate: favor CIK cell therapy.<br>Overall response rate: favor CIK cell therapy.<br>Fever: higher in CIK cell therapy.<br>Quality of life with KPS improvement: favor CIK cell therapy.<br>HBV-DNA content: lower in CIK cell therapy.<br>Plasma AFP content: lower in CIK cell therapy.<br>Ratio of CD3+, CD4+, CD4+CD8+ and CD3+CD4+ T cells: increased in CIK cell therapy.<br>Ratio of CD8+ and CD3+CD8+ T cells: decreased in CIK cell therapy.	CIK cell therapy demonstrated a significant superiority in prolonging the median OS, PFS, DCR, ORR and quality of life of HCC patients.

Supplementary Table S7: Findings of meta-analyses: An overview of included studies regarding tamoxifen

First author	Journal (Year)	Comparisons	OS	DFS, RFS, TTP, PFS	Recurrence, time to recurrence	Other endpoints	Major comments
**Tamoxifen versus placebo**							
Nowak	Cochrane Database Syst Rev (2004); Cancer (2005)	Tamoxifen vs placebo/no intervention	OS:<br>statistically similar.	NA.	NA.	Quality of life, tumor response, toxicity:<br>Only systematic reviews, but not meta-analyses.	There was no support for the therapeutic use of tamoxifen in advanced HCC.
Llovet	Hepatology (2003)	TACE or tamoxifen vs control treatment (including tamoxifen vs control treatment)	OS:<br>Tamoxifen vs control treatment:<br>1-year: statistically similar.	NA.	NA.	NA.	Chemoembolization improves survival of patients with unresectable HCC and may become the standard treatment. Treatment with tamoxifen does not modify the survival of patients with advanced disease.

Supplementary Table S8: Findings of meta-analyses: An overview of included studies regarding antibiotics

First author	Journal (Year)	Comparisons	OS	DFS, RFS, TTP, PFS	Recurrence, time to recurrence	Other endpoints	Major comments
**Antibiotics after transarterial therapy**							
Wang	Can J Gastroenterol (2012)	Prophylactic antibiotic treatment vs no prophylactic antibiotic treatment after transarterial therapy	NA.	NA.	NA.	Fever: statistically similar. Duration of hospital stay: statistically similar. Bacteremia, septicema, sepsis: systematic review, but not meta-analysis.	Antibiotic prophylaxis in patients undergoing transarterial therapy for HCC may not be routinely necessary.

Supplementary Table S9: Overlap of included studies among meta-analyses regarding LDLT versus DDLT

First author	Al Hasan	Grant	Liang
Journal (Year)	Liver Transpl (2014)	Clin Transplant (2013)	Liver Transpl (2012)
Publication type	Abstract	Full text	Full text
No. Included studies	7	16	7
No. Included RCTs	0	0	0
Included studies	NA	Allam N, et al. Exp Clin Transplant 2008;6:14.	Bhangui P, et al. Hepatology 2011;53:1570.
		Azzam AZ, et al. Exp Clin Transplant 2011;9:323.	Di Sandro S, et al. Transplant Proc 2009;41:1283.
		Berg C, et al. Hepatology 2011;54:1313.	Fisher RA, et al. Am J Transplant 2007;7:1601.
		Bhangui P, et al. Hepatology 2011;53:1570.	Hwang S, et al. Liver Transpl 2005;11:1265.
		Chan SC, et al. Ann Surg 2008;248:411.	Li C, et al. Hepatobiliary Pancreat Dis Int 2010;9:366.
		Di Sandro S, et al. Transplant Proc 2009;41:1283.	Lo CM, et al. Br J Surg 2007;94:78.
		Fisher RA, et al. Am J Transplant 2007;7:1601.	Sandhu L, et al. Liver Transpl 2012;18:315.
		Gondolesi GE, et al. Ann Surg 2004;239:142.	
		Hwang S, et al. Liver Transpl 2005;11:1265.	
		Karakayali H, et al. Transplant Proc 2006;38:575.	
		Li C, et al. Hepatobiliary Pancreat Dis Int 2010;9:366.	
		Lo CM, et al. Br J Surg 2007;94:78.	
		Roayaie S, et al. Liver Transpl 2004;10:534.	
		Sandhu L, et al. Liver Transpl 2012;18:315.	
		Sotiropoulos GC, et al. J Am Coll Surg 2007;205:661.	
		Vakili K, et al. Liver Transpl 2009;15:1861.	

Supplementary Table S10: Overlap of included studies among meta-analyses regarding primary versus salvage LT

First author	Li	Zhu
Journal (Year)	World J Gastroenterol (2012)	Transplant Proc (2013)
Publication type	Full text	Full text
No. Included studies	11	14
No. Included RCTs	0	0
Included studies	Adam R, et al. Ann Surg 2003;238:508–518.	Adam R, et al. Ann Surg 2003;238:508–518.
	Belghiti J, et al. Ann Surg 2003; 238:885–892.	Belghiti J, et al. Ann Surg 2003; 238:885–892.
	Concejero A, et al. Transplantation 2008;85:398–406.	Concejero A, et al. Transplantation 2008;85:398–406.
	Del Gaudio M, et al. Am J Transplant 2008;8:1177–1185.	Del Gaudio M, et al. Am J Transplant 2008;8:1177–1185.
	Facciuto ME, et al. Ann Surg Oncol 2008;15:1383–1391.	Facciuto ME, et al. Ann Surg Oncol 2008;15:1383–1391.
	Hwang S, et al. Liver Transpl 2007;13:741–746	Hwang S, et al. Liver Transpl 2007;13:741–746
	Kim BW, et al. Transplant Proc 2008; 40:3558–3561.	Kim BW, et al. Transplant Proc 2008; 40:3558–3561.
	Margarit C, et al. Liver Transpl 2005; 11:1242–1251.	Liu F, et al. PLoS One. 2012;7:e48932.
	Sapisochin G, et al. World J Surg 2010;34:2146–2154.	Margarit C, et al. Liver Transpl 2005; 11:1242–1251.
	Shao Z, et al. World J Gastroenterol 2008;14:4370–4376.	Sapisochin G, et al. World J Surg 2010;34:2146–2154.
	Vennarecci G, et al. Transplant Proc 2007;39:1857–1860.	Scatton O, et al. Liver Transpl 2008; 14:779–788.
		Shao Z, et al. World J Gastroenterol 2008;14:4370–4376.
		Vennarecci G, et al. Transplant Proc 2007;39:1857–1860.
		Wu L, et al. PLoS One. 2012;7:e41820.

Supplementary Table S11: Overlap of included studies among meta-analyses regarding sirolimus-based immunosuppression after LT

First author	Liang	Menon
Journal (Year)	Liver Transpl (2012)	Aliment Pharmacol Ther (2013)
Publication type	Full text	Full text
No. Included studies	5	5
No. Included RCTs	0	0
Included studies	Chinnakotla S, et al. Liver Transpl 2009; 15:1834–1842.	Chinnakotla S, et al. Liver Transpl 2009;15:1834–1842.
	Toso C, et al. Hepatology 2010;51:1237–1243.	Nocera A, et al. Transplant Proc 2008; 40:1950–1952.
	Vivarelli M, et al. Transplantation 2010; 89:227–231.	Toso C, et al. Hepatology 2010;51:1237–1243.
	Zhou J, et al. Transplant Proc 2008;40:3548–3553.	Vivarelli M, et al. Transplantation 2010; 89:227–231.
	Zimmerman MA, et al. Liver Transpl 2008; 14:633–638.	Zimmerman MA, et al. Liver Transpl 2008;14:633–638.

Supplementary Table S12: Overlap of included studies among meta-analyses regarding LT versus surgical resection. See Supplementary Table S12

Supplementary Table S13: Overlap of included studies among meta-analyses regarding laparoscopic versus open resection. See Supplementary_Table_S13

Supplementary Table S14: Overlap of included studies among meta-analyses regarding anatomic versus nonanatomic resection. See Supplementary_Table_S14

Supplementary Table S15: Overlap of included studies among meta-analyses regarding surgical resection + I^{131} lipiodol versus surgical resection alone

First author	Furtado	Gong
Journal (Year)	Ann Surg Oncol (2014)	Nucl Med Commun (2014)
Publication type	Full text	Full text
No. Included studies	5	10
No. Included RCTs	2	2
Included studies	Boucher E, et al. J Nucl Med 2008;49(3):362–6; Hepatology 2003;38:1237–41.	Boucher E, et al. J Nucl Med 2008;49(3):362–6; Hepatology 2003;38:1237–41.
	Chua TC, et al. Cancer 2010;116(17):4069–77.	Chua TC, et al. Int J Clin Oncol 2011;16:125–132.
	Chung AY, et al. World J Surg 2013;37(6): 1356–61.	Chung AY, et al. World J Surg 2013;37(6):1356–61.
	Lau WY, et al. Lancet 1999;353(9155):797–801; Ann Surg 2008;247(1):43–8.	Dupont-Bierre E, et al. J Am Coll Surg2005;201:663–670.
	Tabone M, et al. Eur J Surg Oncol 2007;33(1):61–6.	Keng GH, et al. Ann Acad Med Singapore 2002;31:382–386.
		Lau WY, et al. Lancet 1999;353(9155):797–801; Ann Surg 2008;247(1):43–8.
		Ng KM, et al. HPB (Oxford)2008;10:388–395.
		Partensky C, et al. Arch Surg2000;135:1298–1300.
		Raoul JL, et al. Br J Surg2003;90:1379–1383.
		Tabone M, et al. Eur J Surg Oncol 2007;33(1):61–6.

Supplementary Table S16: Overlap of included studies among meta-analyses regarding surgical resection plus pre-operative TACE

First author	Cheng	Wang	Yu	Zhou
Journal (Year)	J Cancer Res Clin Oncol (2014)	Hepato-gastroenterology (2011)	Chinese-German J Clinical Oncology (2013)	BMC Gastroenterol (2013)
Publication type	Full text	Full text	Full text	Full text
No. Included studies	4	3	7	21
No. Included RCTs	4	3	0	4
Included studies	Kaibori M, et al. Dig Dis Sci 2012;57:1404–1412	Wu CC, et al. Br J Surg 1995;82:122–126.	Choi GH, et al. World J Surg 2007;31:2370–2377.	Adachi E, et al. Cancer 1993;72:3593–3598.
	Wu CC, et al. Br J Surg 1995;82:122–126.	Yamasaki S, et al. Jpn J Cancer Res 1996;2: 206–211.	Gerunda GE, et al. Liver Transpl 2000;6:619–626.	Chen XP, et al. Dig Surg 2007;24:208–213.
	Yamasaki S, et al. Jpn J Cancer Res 1996;2:206–211.	Zhou WP, et al. Ann Surg 2009;249:195–202.	Harada T, et al. Ann Surg 1996;224:4–9.	Choi GH, et al. World J Surg 2007;31:2370–2377.
	Zhou WP, et al. Ann Surg 2009;249:195–202.		Kim IS, et al. Aliment Pharmacol Ther 2008;27: 338–345.	Di Carlo V, et al. Hepatogastroenterology 1998;45:1950–1954.
			Lee KT, et al. J Surg Oncol 2009;99:343–350.	Harada T, et al. Ann Surg 1996;224:4–9.
			Sasaki A, et al. Eur J Surg Oncol 2006;32:773–779.	Kaibori M, et al. Dig Dis Sci 2012;57:1404–1412
			Sugo H, et al. World J Surg 2003;27:1295–1299.	Kang JY, et al. Korean J Hepatol 2010;16:383–388.
				Kim IS, et al. Aliment Pharmacol Ther 2008;27: 338–345.
				Lee KT, et al. J Surg Oncol 2009;99:343–350.
				Lu CD, et al. World J Surg 1999;23:293–300.
				Majno PE, et al. Ann Surg 1997;226:688–701.
				Nagasue N, et al. Surgery 1989;106:81–86.
				Ochiai T, et al. Hepatogastroenterology 2003;50:750–755.
				Paye F, et al. Arch Surg 1998;133:767–772.
				Sasaki A, et al. Eur J Surg Oncol 2006;32:773–779.
				Sugo H, et al. World J Surg 2003;27:1295–1299.
				Uchida M, et al. World J Surg 1996;20:326–331.
				Wu CC, et al. Br J Surg 1995;82:122–126.
				Yamasaki S, et al. Jpn J Cancer Res 1996;2:206–211.
				Yamashita YI, et al. J Surg Oncol 2012;106:498–503.
				Zhou WP, et al. Ann Surg 2009;249:195–202.

Supplementary Table S17: Overlap of included studies among meta-analyses regarding surgical resection plus post-operative TACE

First author	Cheng	Zhong
Journal (Year)	J Cancer Res Clin Oncol (2014)	Hepatol Res (2010)
Publication type	Full text	Full text
No. Included studies	6	6
No. Included RCTs	6	6
Included studies	Izumi R, et al. Hepatology 1994;20:295–301.	Izumi R, et al. Hepatology 1994;20:295–301.
	Li Q, et al. Dig Surg 2006;4:235–240.	Li JQ, et al. J Cancer Res Clin Oncol 1995;121: 364–6.
	Peng BG, et al. Am J Surg 2009;198:313–318.	Li Q, et al. Dig Surg 2006;4:235–240.
	Xu F, et al. Acad J Second Mil Med Univ 2012;33: 274–279.	Peng BG, et al. Am J Surg 2009;198:313–318.
	Yu XZ, et al. J Pract Med 2009;25:1819–1821.	Yu ZP, et al. J Pract Med 2009;25:1819–1821.
	Zhong C, et al. J Cancer Res Clin 2009;10:1437–1445.	Zhong C, et al. J Cancer Res Clin 2009;10: 1437–1445.

Supplementary Table S18: Overlap of included studies among meta-analyses regarding surgical resection plus oral systemic chemotherapy

First author	Mathurin	Zhong
Journal (Year)	Aliment Pharmacol Ther (2003)	Mol Clin Oncol (2014)
Publication type	Full text	Full text
No. Included studies	2	3
No. Included RCTs	1	3
Included studies	Takenaka K, et al. Am J Surg 1995;169:400–405.	Hasegawa K, et al. Hepatology 2006;44:891895.
	Yamamoto M, et al. Br J Surg 1996;83:336–340.	Xia Y, et al. Ann Surg Oncol 2010;17:31373144.
		Yamamoto M, et al. Br J Surg 1996;83:336–340.

Supplementary Table S19: Overlap of included studies among meta-analyses regarding surgical resection plus adjuvant chemotherapy. See Supplementary_Table_S19

Supplementary Table S20: Overlap of included studies among meta-analyses regarding surgical resection plus immunotherapy

First author	Flores	Ma	Xie	Wang
Journal (Year)	J Gastroenterol Hepatol (2009)	Chinese J Cancer Prevention and Treatment (2011)	PLoS One (2012)	Can J Gastroenterol (2013)
Publication type	Full text	Full text	Full text	Full text
No. Included studies	2	4	6	3
No. Included RCTs	2	4	6	3
Included studies	Not reported	Dong H, et al. Dig Liver Dis 2009;41(1):36–41.	Hui D, et al. Dig Liver Dis 2009;41:36–41.	Hui D, et al. Dig Liver Dis 2009;41:36–41.
		Takayama T, et al. Lancet 2000;356(9232):802–807.	Kawata A, et al. Am J Clin Oncol 1995;18:257–262.	Takayama T, et al. Lancet 2000;356(9232):802–807.
		Weng DS, et al. J Immunother 2008;31(1):63–71.	Lu B, et al. Journal of Medical Forum 2008;29:69–70.	Weng DS, et al. J Immunother 2008;31(1):63–71.
		Zhou WP, et al. Zhonghua Wai Ke Za Zhi 1995;33(1):35–37.	Takayama T, et al. Lancet 2000;356(9232):802–807.	
			Xie L, et al. Zhonghua Gan Zang Bing Za Zhi 2000;8:142–143.	
			Zhou WP, et al. Chinese-German JournaI of Clinical Oncology 2000;1:163–165.	

Supplementary Table S21: Overlap of included studies among meta-analyses regarding RFA versus surgical resection. See Supplementary_Table_S21

Supplementary Table S22: Overlap of included studies among meta-analyses regarding PEI versus surgical resection

First author	Hoshida	Schoppmeyer
Journal (Year)	Hepatology (2000)	Cochrane Database Syst Rev (2009)
Publication type	Full text	Full text
No. Included studies	5	1
No. Included RCTs	0	1
Included studies	Livraghi T, et al. J Hepatol 1995;22:522–526.	Huang GT, et al. Ann Surg 2005;242(1):36–42.
	Castells A, et al. Hepatology 1993;18: 1121–1126.	
	Kotoh K, et al. Am J Gastroenterol 1994;89: 194–198.	
	Ryu M, et al. Jpn J Clin Oncol 1997;27:251–257.	
	Sakamoto M, et al. Jpn J Clin Oncol 1998;28: 604–608.	

Supplementary Table S23: Overlap of included studies among meta-analyses regarding RFA versus PEI or PAI. See Supplementary_Table_S23

Supplementary Table S24: Overlap of included studies among meta-analyses regarding PEI versus PAI

First author	Germani	Schoppmeyer
Journal (Year)	J Hepatol (2010)	Cochrane Database Syst Rev (2009)
Publication type	Full text	Full text
No. Included studies	2	2
No. Included RCTs	2	2
Included studies	Ohnishi K, et al. Hepatology 1998;27:67–72.	Ohnishi K, et al. Hepatology 1998;27:67–72.
	Huo TI, et al. Scand J Gastroenterol 2003;38:770–778.	Lin SM, et al. Gut 2005;54:1151–1156.

Supplementary Table S25: Overlap of included studies among meta-analyses regarding RFA plus TACE. See Supplementary_Table_S25

Supplementary Table S26: Overlap of included studies among meta-analyses regarding PEI plus TACE

First author	Liao	Wang N	Wang W
Journal (Year)	PLoS One (2013)	Med Oncol (2011)	Liver Int (2010)
Publication type	Full text	Full text	Full text
No. Included studies	4	7	6
No. Included RCTs	4	7	6
Included studies	Bartolozzi C, et al. Radiology 1995;197:812–818.	Bartolozzi C, et al. Radiology 1995;197:812–818.	Bartolozzi C, et al. Radiology 1995;197:812–818.
	Becker G, et al. World J Gastroenterol 2005;11:6104–6109.	Becker G, et al. World J Gastroenterol 2005;11:6104–6109.	Becker G, et al. World J Gastroenterol 2005;11: 6104–6109.
	Xu GH, et al. Clin Radiol 2002;21:66–68.	Kato T, et al. Cancer Chemother Pharmacol 1994;33:115–118.	Francesco S, et al. Radiol Med 2004;108:356–371.
	Yamamoto K, et al. Semin Oncol 1997;24:S6–50-S6–55.	Li HB, et al. Med Sci Edi 2008;27:368–370.	Koda M, et al. Cancer 2001;92:1516–1524.
		Li XY, et al. Chin J Interv Imaging Ther 2007;4:269–272.	Wu PH, et al. Chin J Oncol 1998;5:391–393.
		Qu ZQ, et al. Chinese–German J Clin Oncol 2002;1:28–9.	Xu GH, et al. Clin Radiol 2002;21:66–68.
		Zhao XL, et al. J Fourth Mil Med Univ 2004;25:1382.	

Supplementary Table S27: Overlap of included studies among meta-analyses regarding any ablation therapy plus TACE

First author	Gu	Wang
Journal (Year)	J Cancer Res Clin Oncol (2014)	Liver Int (2010)
Publication type	Full text	Full text
No. Included studies	18	10
No. Included RCTs	7	10
Included studies	Allgaier HP, et al. Int J Cancer 1998;79:601–605.	Aikata H, et al. Hepatology 2006;4:A487.
	Bartolozzi C, et al. Radiology 1995;197:812–818.	Bartolozzi C, et al. Radiology 1995;197: 812–818.
	Becker G, et al. World J Gastroenterol 2005;11: 6104–6109.	Becker G, et al. World J Gastroenterol 2005;11: 6104–6109.
	Huo TI, et al. Ann Oncol 2003;14(11):1648–1653.	Francesco S, et al. Radiol Med 2004;108: 356–371.
	Kamada K, et al. Am J Surg 2002;184:284–290.	Koda M, et al. Cancer 2001;92:1516–1524.
	Kato T, et al. Cancer Chemother Pharmacol 1994;33:S115–S118.	Shibata T, et al. Radiology 2009;252:905–913.
	Kim JW, et al. Eur J Radiol 2012;81(3):e189–e193.	Wang YB, et al. Qual Life Res 2007;16: 389–397.
	Koda M, et al. Cancer 2001;92:1516–1524.	Wu PH, et al. Chin J Oncol 1998;5:391–393.
	Li C, et al. Eur J Cancer 2010;46:2513–2521.	Xu GH, et al. Clin Radiol 2002;21:66–68.
	Morimoto M, et al. Cancer 2010;116:5452–5460.	Yang P, et al. Adv Ther 2008;25:787–794.
	Peng ZW, et al. Eur J Surg Oncol 2009;36(3): 257–263.	
	Peng ZW, et al. Radiology 2012;262(2):689–700.	
	Peng ZW, et al. J Clin Oncol 2013;31:426–432.	
	Shibata T, et al. Radiology 2009;252:905–913.	
	Wu F, et al. Radiology 2005;235:659–667.	
	Xu KC, et al. World J Gastroenterol 2009;15(29): 3664–3669.	
	Yang P, et al. Adv Ther 2008;25:787–794.	
	Yang W, et al. Hepatol Res 2009;39(3):231–240.	

Supplementary Table S28: Overlap of included studies among meta-analyses regarding TACE or TAE versus no active treatment. See Supplementary_Table_S28

Supplementary Table S29: Overlap of included studies among meta-analyses regarding TACE versus TAE

First author	Camma	Marelli	Xie
Journal (Year)	Radiology (2002)	Cardiovasc Intervent Radiol (2007)	Tumour Biol (2014)
Publication type	Full text	Full text	Full text
No. Included studies	2	3	5
No. Included RCTs	2	3	5
Included studies	Chang JM, et al. Cancer 1994;74:2449–2453.	Chang JM, et al. Cancer 1994;74:2449–2453.	Chang JM, et al. Cancer 1994;74:2449–2453.
	Kawai S, et al. Cancer Chemother Pharmacol 1992;31(suppl 1):S1–S6.	Llovet JM, et al. Lancet 2002;359(9319):1734–1739.	Kawai S, et al. Cancer Chemother Pharmacol 1992;31(suppl 1):S1–S6.
		Lo CM, et al. Hepatology 2002;35:1164–1171.	Llovet JM, et al. Lancet 2002;359(9319):1734–1739.
			Malagari K, et al. Cardiovasc Intervent Radiol 2010;33:541–551.
			Meyer T, et al. Br J Cancer. 2013;108:1252–1259.

Supplementary Table S30: Overlap of included studies among meta-analyses regarding DEB-TACE versus conventional TACE

First author	Gao	Han	Huang
Journal (Year)	Hepatogastroenterology (2013)	PLoS One (2014)	J Gastroenterol Hepatol (2014)
Publication type	Full text	Full text	Full text
No. Included studies	2	5	7
No. Included RCTs	0	3	2
Included studies	Dhanasekaran R, et al. J Surg Oncol 2010;101:476–480.	Lammer J, et al. Cardiovasc Intervent Radiol 2010;33:41–52.	Dhanasekaran R, et al. J Surg Oncol 2010;101:476–480.
	Song MJ, et al. Eur J Gastroenterol Hepatol 2011;23:521–527.	Sacco R, et al. J Vasc Interv Radiol 2011;22:1545–1552.	Ferrer PM, et al. Radiologia 2011;53: 246–253.
		Song MJ,et al. J Hepatol 2012;57:1244–1250.	Malagari K, et al. Cardiovasc Intervent Radiol 2010;33:541–551.
		van Malenstein H, et al. Onkologie 2011;34:368–376.	Meyer T, et al. Br J Cancer. 2013;108: 1252–1259.
		Wiggermann P, et al. Med Sci Monit 2011;17:CR189–195.	Recchia F, et al. Oncol Rep 2012;27: 1377–1383.
			Sacco R, et al. J Vasc Interv Radiol 2011;22:1545–1552.
			Song MJ,et al. J Hepatol 2012;57:1244–1250.
			Vogl TJ, et al. AJR Am J Roentgenol 2011;197:W562–70. Lammer J, et al. Cardiovasc Intervent Radiol 2010;33:41–52. Lencioni R, et al. Hepatology 2009;50:1079A.
			Wiggermann P, et al. Med Sci Monit 2011;17:CR189–195.

Supplementary Table S31: Overlap of included studies among meta-analyses regarding TACE + sorafenib versus TACE alone

First author	Fu	Liu	Yang	Zhang
Journal (Year)	J Cancer Res Clin Oncol (2014)	PLoS One (2014)	Mol Biol Rep (2014)	PLoS One (2014)
Publication type	Full text	Full text	Full text	Full text
No. Included studies	9	7	6	6
No. Included RCTs	0	3	3	2
Included studies	Chen SM, et al. China J Mod Med 2012;22(25):71–73.	Bai W, et al. J Dig Dis 2013;14:181–190.	Bai W, et al. J Dig Dis 2013;14: 181–190.	Bai W, et al. J Dig Dis 2013;14:181–190.
	Jiang HY, et al. Hainan Med J 2010;21(23):6–9.	Huang YH, et al. Chin Med J 2013;126:385–386.	Kudo M, et al. Eur J Cancer 2011;47(14): 2117–2127.	Choi GH, et al. Radiology 2013;269:603–611.
	Kudo M, et al. Eur J Cancer 2011;47(14):2117–2127.	Kudo M, et al. Eur J Cancer 2011;47(14):2117–2127.	Lencioni R, et al. J Clin Oncol supp4: abs LBA154	Kudo M, et al. Eur J Cancer 2011;47(14): 2117–2127.
	Lu L, et al. Chin J Tissue Eng Res 2012;16(31):5706–5710.	Lencioni R, et al. J Clin Oncogene 2012;30.	Qu XD, et al. BMC Cancer 2012;12:263.	Muhammad A, et al. World J Hepatol 2013;5:364–371.
	Qi YY, et al. Mod Oncol 2010;18(11):2188–2190.	Martin RC II, et al. Abstract 216. ASCO Gastrointestinal Cancers Symposium January 22–24. 2010	Sansonno D, et al. Oncologist 2012;17(3): 359–366.	Qu XD, et al. BMC Cancer 2012;12:263.
	Qu XD, et al. BMC Cancer 2012;12:263.	Muhammad A, et al. World J Hepatol 2013;5:364–371.	Tan WF, et al. Acta Pharmacol Sin 2010;31:1643–1648.	Sansonno D, et al. Oncologist 2012;17(3):359–366.
	Sansonno D, et al. Oncologist 2012;17(3):359–366.	Sansonno D, et al. Oncologist 2012;17(3):359–366.		
	Wei XY, et al. Pract Clin Med 2009;10(11):17–19.			
	Wu XB, et al. J Hepatopanereatobiliary Surg 2010;22(6): 466–468.			

Supplementary Table S32: Overlap of included studies among meta-analyses regarding TACE+HIFU versus TACE

First author	Cao	Liao
Journal (Year)	Ultrasound Med Biol (2011)	PLoS One (2013)
Publication type	Full text	Full text
No. Included studies	9	5
No. Included RCTs	0	1
Included studies	Cao W, et al. Modern Oncol (Chinese) 2009;17:1930–1932.	Li CX, et al. Zhonghua Yi Xue Za Zhi (Chinese) 2009;89:754–757.
	Chen WZ, et al. The Fourth National Symposium of interventional treatment of malignant digestive tract disease (Chinese)2005;90–91.	Liu JT. Chin J Misdiagn (Chinese) 2008;8:7333–7334
	Jin CB, et al. Zhonghua Zhong Liu Za Zhi (Chinese) 2003;25:401–403.	Peng DW, et al. China Tropical Med (Chinese) 2009;9:1256–1257.
	Li CX, et al. Zhonghua Yi Xue Za Zhi (Chinese) 2009;89:754–757.	Ye X, et al. Cancer Res Clin (Chinese) 2008;20:268–271.
	Liu JT. Chin J Misdiagn (Chinese) 2008;8: 7333–7334	Zhang GX, et al. Chin J Ultrasound Med (Chinese) 2005;21:71–73.
	Peng DW, et al. China Tropical Med (Chinese) 2009;9:1256–1257.	
	Xu J, et al. Shandong Yiyao (Chinese) 2006;46: 79–80.	
	Ye X, et al. Cancer Res Clin (Chinese) 2008;20:268–271.	
	Zhang GX, et al. Chin J Ultrasound Med (Chinese) 2005;21:71–73.	

Supplementary Table S33: Overlap of included studies among meta-analyses regarding TACE + radiotherapy versus TACE

First author	Meng	Liao
Journal (Year)	Radiother Oncol (2009)	PLoS One (2013)
Publication type	Full text	Full text
No. Included studies	17	7
No. Included RCTs	5	3
Included studies	Chia-Hsien Cheng J, et al. Int J Cancer 2001;96: 243–252.	Chia-Hsien Cheng J, et al. Int J Cancer 2001;96:243–252.
	Guo WJ, et al. World J Gastroenterol 2003;9: 1697–1701.	Guo WJ, et al. World J Gastroenterol 2003;9:1697–1701.
	Lan DQ, et al. Chin J Radiat Oncol 2005;14:152–153.	Leng ZQ, et al. Chin J Radiat Oncol 2000;9:99–101.
	Leng ZQ, et al. Chin J Radiat Oncol 2000;9:99–101.	Shim SJ, et al. Liver Int 2005;25:1189–1196.
	Li Y, et al. Chin J Radiat Oncol 2003;12:30–32.	Song SM, et al. Chin J Clin Oncol 2002;29:141–142.
	Liu MZ, et al. Ai Zheng 2005;24:82–86.	Wang G, et al. Int J Clin Oncol 2000;5: 380–385.
	Peng KG, et al. Chin J Radiat Oncol 2000;9:11–13.	Wang XH, et al. Shaxi Yixue Zazhi 2006;35:461–462.
	Shang Y, et al. Shijie Xiaohua Zazhi 2007;15: 3140–3142.	
	Shim SJ, et al. Liver Int 2005;25:1189–1196.	
	Song SM, et al. Chin J Clin Oncol 2002;29:141–142.	
	Wang G, et al. Int J Clin Oncol 2000;5:380–385.	
	Wang GM, et al. Chin J Radiat Oncol 1992;1:148–50.	
	Wang XH, et al. Shaxi Yixue Zazhi 2006;35:461–462.	
	Wu DH, et al. Chin J Dig 2004;24:353–357.	
	Xue HZ, et al. Chin J Radiat Oncol 1995;4:84–85.	
	Zeng ZC, et al. Cancer 2004;10:307–316.	
	Zhao MH, et al. Chin J Radiat Oncol 2006;15:39–41.	

Supplementary Table S34: Overlap of included studies among meta-analyses regarding TACE + traditional Chinese medicine versus TACE alone. See Supplementary_Table_S34

Supplementary Table S35: Overlap of included studies among meta-analyses regarding TACE + CIK cell therapy versus TACE

First author	Chen	Li
Journal (Year)	Chinese-German J Clin Oncol (2013)	Clin Res Hepatol Gastroenterol (2014)
Publication type	Full text	Full text
No. Included studies	9	11
No. Included RCTs	9	6
Included studies	Guo P, et al. Chin Hosp Pharm J (Chinese) 2007;27:1565–1567.	Deng WJ, et al. Ling Nan Xian Dai Ling Chuang Wai Ke 2013;13:29–31.
	Hao MZ, et al. Chin J Cancer 2010;29:172–177.	Hao MZ, et al. Ai Zheng 2010;29:182–189.
	He XB, et al. Sichuan Med J (Chinese) 2012;33:1696–1698.	Hao MZ, et al. Zhong Guo Zhong Liu Sheng Wu Zhi Liao Za Zhi 2006;13:303–305.
	Huang Z, et al. Yiyao Qianyan (Chinese) 2013;4:140–141.	He XB, et al. Si Chuan Xi Xue 2012;33:1696–1697.
	Shi Y, et al. J Intervent Radiol (Chinese) 2007;16:235–239.	Huang LX, et al. Guang Dong Yi Xue 2007;28:1466–1468.
	Wang FL, et al. Med Philos (Clin Dec Mak Forum Edit) (Chinese) 2011;32:27–28.	Huang ZM, et al. J Immunother 2013;36:287–93.
	Xing H, et al. Inter J Dig Dis (Chinese) 2011;31:121–124.	Tong LQ, et al. Zhong Guo Pu Tong Wai Ke Za Zhi 2013;22:876–879.
	Yu WC, et al. J Mini Inva Med (Chinese) 2009;4:459–461.	Pan CC, et al. Ai Zheng 2010;29:596–602.
	Zhang NZ, et al. J Southeast Chin Nat Defe Med Sci (Chinese) 2006;8:84–87.	Wang JP, et al. Zhonghua Xi Xue Za Zhi 2012;92:3062–3066.
		Zhang NZ, et al. J Southeast Chin Nat Defe Med Sci (Chinese) 2006;8:84–87.
		Zhao M, et al. Zhonghua Xi Xue Za Zhi 2006;86:1823–1828.

Supplementary Table S36: Overlap of included studies among meta-analyses regarding sorafenib. See Supplementary_Table_S36

Supplementary Table S37: Overlap of included studies among meta-analyses regarding antiviral therapy. See Supplementary_Table_S37

Supplementary Table S38: Overlap of included studies among meta-analyses regarding vitamin

First author	Chu	Meng	Riaz	Wang	Zhong
Journal (Year)	Asian J Surg (2010)	Hepatol Int (2011)	BMC Gastroenterol (2012)	Can J Gastroenterol (2013)	World Chinese J of Digestology (2012); PLoS One (2013)
Publication type	Full text	Abstract	Full text	Full text	Full text
No. Included studies	6	4	5	6	7
No. Included RCTs	5	4	5	6	6
Included studies	Muto Y, et al. N Engl J Med 1996;334:1561–1567.	N/A	Mizuta T, et al. Cancer 2006;106:867–872.	Muto Y, et al. N Engl J Med 1996;334:1561–1567.	Mizuta T, et al. Cancer 2006;106:867–872.
	Mizuta T, et al. Cancer 2006;106:867–872.		Kakizaki S, et al. J Gastroenterol Hepatol 2007;22:518–522.	Muto Y, et al. N Engl J Med 1999;340:1046–1047.	Hotta N, et al. Hepatogastroenterology 2007;54:2073–2077.
	Takai K, et al. Intervirology 2005;48:39–45.		Hotta N, et al. Hepatogastroenterology 2007;54:2073–2077.	Mizuta T, et al. Cancer 2006;106:867–872.	Kakizaki S, et al. J Gastroenterol Hepatol 2007;22:518–522.
	Kakizaki S, et al. J Gastroenterol Hepatol 2007;22:518–522.		Yoshiji H, et al. J Hepatol 2009;51: 315–321.	Hotta N, et al. Hepatogastroenterology 2007;54:2073–2077.	Yoshida H, et al. Hepatology 2011;54(2):532–540.
	Hotta N, et al. Hepatogastroenterology 2007;54:2073–2077.		Yoshida H, et al. Hepatology 2011;54(2):532–540.	Kakizaki S, et al. J Gastroenterol Hepatol 2007;22:518–522.	Yoshiji H, et al. J Hepatol 2009;51:315–321.
	Yoshiji H, et al. J Hepatol 2009;51:315–321.			Yoshida H, et al. Hepatology 2011;54(2):532–540.	Hosho K, et al. Yonago Acta medica 2006;51:95–99.
					Kubota K, et al. Eur J Cancer 2011;47 (SUPPL. 1):S470.

Supplementary Table S39: Overlap of included studies among meta-analyses regarding octreotide

First author	Estanislao	Guo	Ji
Journal (Year)	J Gastroenterol Hepatol (2009)	J Cancer Res Clin Oncol (2009)	Med Sci Monit (2011)
Publication type	Abstract	Full text	Full text
No. Included studies	3	6	11
No. Included RCTs	NA	6	9
Included studies	N/A	Becker G, et al. Hepatology 2007;45:9–15.	Barbare JC, et al. Eur J Cancer 2009;45:1788–1797.
		Dimitroulopoulos D, et al. World J Gastroenterol 2007;13:3164–3170.	Becker G, et al. Hepatology 2007;45:9–15.
		Farooqi JI, et al. J Coll Physicians Surg Pak 2000;10:258–260.	Dimitroulopoulos D, et al. World J Gastroenterol 2007;13:3164–3170.
		Kouroumalis E, et al. Gut 1998;42:442–447.	Farooqi JI, et al. J Coll Physicians Surg Pak 2000;10:258–260.
		Yuen MF, et al. Hepatology 2002;36:687–691.	Kouroumalis E, et al. Gut 1998;42:442–447.
		Ou SQ, et al. Hainan Med J 2007;18:19–20.	Ou SQ, et al. Hainan Med J 2007;18:19–20.
			Wu P, et al. Chin J Hepatobiliary Surg 2001;7:766–768.
			Yang MN, et al. J Clin Med in Pract 2003;7:302–304.
			Yuen MF, et al. Hepatology 2002;36:687–691.
			Zhang B, et al. J Basic Clin Oncol 2010;23:52–54.
			Zhang L, et al. Chin Clin Oncol 2004;9:514–517.

Supplementary Table S40: Overlap of included studies among meta-analyses regarding tamoxifen

First author	Nowak	Llovet
Journal (Year)	Cochrane Database Syst Rev (2004); Cancer (2005)	Hepatology (2003)
Publication type	Full text	Full text
No. Included studies	10	7
No. Included RCTs	10	7
Included studies	Barbare J-C, et al. Proc Am Soc Clin Oncol. 2002;21:138A.	Castells A, et al. Gastroenterology 1995;109:917–922.
	Castells A, et al. Gastroenterology 1995;109:917–922.	CLIP Group. Lancet 1998;352:17–20.
	Chow PK, et al. Hepatology 2002;36:1221–1226.	Elba S, et al. Ital J Gastroenterol 1994;26:66–68.
	CLIP Group. Lancet 1998;352:17–20.	Liu CL, et al. Am J Gastroenterol 2000;95:218–222.
	Coll S, et al. Hepatology 1995;4:404A.	Manesis EK, et al. Hepatology 1995;21:1535–1542.
	Elba S, et al. Ital J Gastroenterol 1994;26:66–68.	Martinez-Cerezo FJ, et al. J Hepatol 1994;20:702–706.
	Liu CL, et al. Am J Gastroenterol 2000;95:218–222.	Riestra S, et al. J Clin Gastroenterol 1998;26:200–203.
	Martinez-Cerezo FJ, et al. J Hepatol 1994;20:702–706.	
	Melia WM, et al. Cancer Treat Rep 1987;71: 1213–1216.	
	Riestra S, et al. J Clin Gastroenterol 1998;26:200–203.	
