# Supplementary material for: Management of hepatocellular carcinoma: an overview of major findings from meta-analyses
Source: Oncotarget. 2016 May 4;7(23):34703–51. doi: 10.18632/oncotarget.9157 (PMC5085185; doi:10.18632/oncotarget.9157)
Supplement: Supplementary file 2 [file oncotarget-07-34703-s002.docx]

**Supplementary Table S2: Findings of meta-analyses:** An overview of included studies regarding antiviral therapy

| **First author** | **Journal (Year)** | **Comparisons** | **OS** | **DFS, RFS, TTP, PFS** | **Recurrence, time to recurrence** | **Other endpoints** | **Major comments** |
| --- | --- | --- | --- | --- | --- | --- | --- |
| **Antiviral therapy, including interferon (IFN), nucleot(s)ide analogues (NA)** | | | | | | | |
| Breitenstein | Br J Surg (2009) | IFN after resection or ablation | OS: favor IFN. | NA. | Recurrence:  lower in IFN. | NA. | IFN has a significant beneficial effect after curative treatment of HCC in terms of both survival and tumor recurrence. |
| Huang | J Viral Hepat (2013) | Adjuvant IFN therapy after curative treatment | OS: RCTs: favor IFN therapy. Subgroup - RCTs - HCV: favor IFN therapy. Subgroup - RCTs - HBV: statistically similar. Non-RCTs: favor IFN therapy. Subgroup - Non-RCTs - HCV: favor IFN therapy. Subgroup - Non-RCTs - HBV: favor IFN therapy. | RFS: RCTs: statistically similar. Subgroup - RCTs - HCV: statistically similar. Subgroup - RCTs - HBV: statistically similar. Non-RCTs: favor antiviral therapy. Subgroup - Non-RCTs - HCV: favor antiviral therapy. Subgroup - Non-RCTs - HBV: statistically similar. | NA. | NA. | Adjuvant IFN therapy significantly improves the RFS and OS of patients with HCV-related HCC following curative treatment. |
| Jiang | World J Surg Oncol (2013) | Adjuvant IFN therapy after treatment with surgical resection or TACE | Death: lower in IFN. Subgroup - surgical resection: lower in IFN. Subgroup - TACE: lower in IFN. | NA. | Recurrence: lower in IFN. Subgroup - surgical resection: statistically similar. Subgroup - TACE: lower in IFN. | NA. | Adjuvant IFN therapy may significantly reduce the recurrence rates of patients with viral hepatitis-related HCC and improve the survival of patients after surgical resection or TACE. |
| Lan | J Gastroenterol Hepatol Res (Hong Kong) (2013) | Lamivudine treatment vs no antiviral therapy after liver resection or RFA | OS: 1-year: statistically similar. | Tumor-free survival: 1-year: favor lamivudine. | Recurrence: lower in lamivudine. | NA. | Lamivudine after initial treatment for HCC reduce recurrence rate and promoted tumor free survival rates, but it did not contribute to short-term OS. |
| Li | Chinese J Cancer Prevention and Treatment (2013) | Adjuvant IFN vs without IFN after curative treatment | OS: Subgroup - HCV:  1-year: statistically similar. 3-year: statistically similar. 5-year: favor IFN. Subgroup - HBV: 1-year: statistically similar. 3-year: statistically similar. 5-year: statistically similar. | NA. | Recurrence: Subgroup - HCV:  2-year: favor IFN. Subgroup - HBV: 2-year: statistically similar. | NA. | IFN can reduce the early recurrence and improve the 5 year OS in HCV related HCC. |
| Miao | World J Gastroenterol (2010) | Adjuvant antiviral therapy after curative therapy | OS: 1-, 2-, 3-, 5-, 7-year: favor antiviral therapy. | NA. | Recurrence: 1-, 2-, 3-, 5-year: fewer in antiviral therapy. | NA. | The post-operative antiviral therapy, IFN in particular, may serve as a favorable alternative to reduce recurrence and mortality in patients with HBV/HCV related HCCs. |
| Miyake | J Viral Hepat (2010) | IFN-alpha after curative therapy | NA. | NA. | Recurrence:  lower in IFN. | NA. | IFN-alpha treatment after curative treatment of primary tumor within Milan criteria may be effective for the prevention of HCC recurrence. |
| Moriguchi | Hepatology (2006) | Tumor ablation plus IFN therapy | Death: lower in IFN. | NA. | NA. | NA. | The tumor ablation plus IFN therapy for patients with HBV or HCV-related resectable HCC is useful. |
| Shen | J Hepatol (2010) | Adjuvant IFN therapy after curative therapy | NA. | RFS: 1-, 2-, 3-year: favor IFN. | NA. | NA. | Lower percentage of patients with multiple tumors and use of ablation therapy were independent predictors for better treatment efficacy. |
| Singal | Aliment Pharmacol Ther (2010) | IFN after resection or ablation | OS: favor IFN. | NA. | A new focus of HCC recurrence: favor IFN. | NA. | IFN treatment after curative resection or ablation of HCC in HCV-related cirrhotics prevents HCC recurrence and improves survival. |
| Sun | PLoS One (2014) | NAs vs placebo or no treatment after curative treatment | OS: favor NAs. Subgroup - RCTs: favor NAs. Subgroup - cohort studies: favor NAs. | NA. | RFS: favor NAs. Subgroup - RCTs: favor NAs. Subgroup - cohort studies: favor NAs. | NA. | Our study suggested benefits of adjuvant NAs therapy following curative treatment of HBV-HCC. |
| Wang | Can J Gastroenterol (2013) | Different adjuvant therapy after potentially curative treatment (including IFN therapy) | OS: IFN therapy: favor IFN therapy. | RFS: IFN therapy: favor IFN therapy. | NA. | NA. | Adjuvant IFN therapy can improve both RFS and OS; however, the benefits of using this agent should be weighed against its side effects. |
| Wong | Aliment Pharmacol Ther (2011) | Antiviral treatment vs no anti-viral treatment for HBV-related HCC | Overall mortality: favor antiviral treatment. Mortality related to liver failure:  favor antiviral treatment. HCC-related mortality: statistically similar. | NA. | HCC recurrence: favor antiviral treatment. | NA. | Anti-viral therapy has potential beneficial effects after the curative treatment of HBV-related HCC in terms of tumor recurrence, liver-related mortality and OS. |
| Xu | Hepatol Res (2014) | Adjuvant IFN therapy after surgical treatment | OS: Subgroup - HBV: 1-, 2-, 3-, 5-year: favor IFN. Subgroup - HCV: 1-, 2-, 3-, 5-year: favor IFN. | NA. | Recurrence: Subgroup - HBV: 1-year: favor IFN. 2-, 3-, 5-year: statistically similar. Subgroup - HCV: 1-, 2-, 3-, 5-year: favor IFN. | NA. | IFN therapy shows a significant clinical effect in postoperative patients of HCC, particularly in HCV-related HCC. |
| Zhang | Mol Clin Oncol (2014) | Adjuvant IFN after curative surgery or ablation therapy | Mortality: favor IFN. | NA. | HCC recurrence: 1-, 2-year: lower in IFN. Late recurrence (>2 year): statistically similar. | NA. | The effect of adjuvant IFN on postoperative recurrence differed between HBV-HCC and HCV-HCC cases. |
| Zhang | Int J Cancer (2009) | IFN-alpha with placebo or no treatment after tumor resection or ablation | Overall 1-year survival: favor IFN. 1-year survival: favor IFN. | NA. | Overall early recurrence: lower in IFN. Early recurrence: lower in IFN. | NA. | The use of IFN-a as adjuvant postsurgical or ablative treatment seems promising but requires further study. |
| Zhou | World J Surg (2014) | Antiviral therapy NAs for HBV-related HCC after curative resection | OS: favor NAs. | DFS: favor NAs. | Recurrence: favor NAs. | NA. | NAs therapy improves the prognosis of HBV-related HCC after resection. |
| Zhuang | PLoS One (2013) | IFN after curative therapy | NA. | NA. | Recurrence: 1-, 2-, 3-, 4-, 5-year: favor IFN. | NA. | After curative therapies, adjuvant IFN reduced the recurrence of HCC. |
| Zhuang | Zhonghua Gan Zang Bing Za Zhi (2012) | IFN after curative therapy | OS: 1-, 2-, 3-, 4-, 5-year: statistically similar. | NA. | Recurrence: 1-, 3-, 4-year: favor IFN. 2-, 5-year: statistically similar. | NA. | IFN therapy after the treatment of resection, ablation or TACE can probably reduce HCC recurrence rate and improve survival with acceptable toxicities. |
