# Supplementary material for: Management of hepatocellular carcinoma: an overview of major findings from meta-analyses
Source: Oncotarget. 2016 May 4;7(23):34703–51. doi: 10.18632/oncotarget.9157 (PMC5085185; doi:10.18632/oncotarget.9157)
Supplement: Supplementary file 3 [file oncotarget-07-34703-s003.docx]

**Supplementary Table S12: Overlap of included studies among meta-analyses regarding LT versus surgical resection**

| **First author** | **Dhir** | **Hoshida** | **Proneth** | **Rahman** | **Xu** | **Xu** | **Zheng** |
| --- | --- | --- | --- | --- | --- | --- | --- |
| Journal (Year) | HPB (2012) | Hepatology (2000) | Ann Surg Oncol (2014) | J Gastrointest Surg (2012) | Hepatobiliary Pancreat Dis Int (2014) | Journal of Xi’an Jiaotong University (Medical Sciences) (2012) | Transplantation (2014) |
| Publication type | Full text | Full text | Full text | Full text | Full text | Full text | Full text |
| No. Included studies | 10 | 12 | 7 | 9 | 17 | 9 | 62 (50*) |
| No. Included RCTs | 0 | 0 | 0 | 0 | 0 | 0 | 0 |
| Included studies | Baccarani U, et al. Transpl Int 2008;21: 247–254. | Bismuth H, et al. Ann Surg 1993;218: 145–151. | Bigourdan JM, et al. Liver Transpl 2003;9 :513–520. | Adam R, et al. Ann Surg 2003;238: 508–518. | Adam R,  et al. Ann Surg 2003;238: 508–519. | Baccarani U,  et al. Transpl Int. 2008;21:247–254. | Adam R, et al. Ann Surg 2003;238:508–518. |
|  | Bellavance EC, et al. J Gastrointest Surg 2008;12: 1699–1708. | Bronowicki JP, et al.  J Hepatol 1996; 24:293–300. | Del Gaudio M, et al. Am J Transplant 2008;8: 1177–1185. | De Carlis L,  et al. Chirurgia Italiana 2001;53: 579–586. | Baccarani U,  et al. Transplant Proc 2007;39: 1898–1900. | Bronowicki  JP,  et al.  J Hepatol 1996; 24:293300. | Baccarani U, et al. Transplant Proc 2007;39:1898–1900. |
|  | Bigourdan JM, et al. Liver Transpl 2003;9: 513–520. | Iwatsuki S, et al. Ann Surg 1991;214: 221–229. | Facciuto ME, et al. HPB (Oxford). 2009;11 :398–404. | Del Gaudio M, et al. Am J Transplant 2008;8: 1177–1185. | Bellavance EC, et al. J Gastrointest  Surg 2008; 12:1699–1708. | González HD, et al. Clin Transl Oncol. 2009;11:20–27. | Bellavance EC, et al. J Gastrointest Surg 2008;12:1699–1708. |
|  | Del Gaudio M, et al. Am J Transplant 2008;8: 1177–1185. | Llovet JM, et al. Hepatology 1999;30:1434–1440. | Koniaris LG, et al. Ann Surg. 2011;254 :527–537. | Figueras J,  et al. J Am Coll Surg 2000;190: 580–587. | Bigourdan JM, et al. Liver Transpl 2003;9:513–520. | Gu W. Fudan University thesis 2008 | Bigourdan JM, et al. Liver Transpl 2003;9:513–520. |
|  | Lee KK, et al. J Surg Oncol 2010;101 :47–53. | Mazziotti A, et al. Hepatogastroenterology 1998;45:1281–1287. | Llovet JM, et al. Hepatology 1999;30: 1434–1440. | Koniaris LG, et al. Ann Surg. 2011;254 :527–537. | Bronowicki  JP, et al. J Hepatol 1996;24 :293–300. | Iwatsuki S, et al. Ann Surg 1991; 214:221229. | Borie F, et al. J Surg Oncol 2008;98:505–509. |
|  | Llovet JM,  et al. Hepatology 1999;30: 1434–1440. | Michel J, et al.  J Hepatol 1997; 26:1274–1280. | Poon RT, et al.  Ann Surg 2007; 245:51–58. | Lee KK, et al. J Surg Oncol 2010;101 :47–53. | De Carlis L,  et al. J Am Coll Surg 2003;196 :887–897. | Llovet JM, et al. Hepatology 1999; 30:14341440. | Bronowicki  JP, et al.  J Hepatol 1996; 24:293–300. |
|  | Margarit C, et al. Liver Transpl 2005; 11:1242–1251. | Otto G, et al. Ann Surg 1998;227:424–432. | Shah SA, et al. Ann Surg Oncol 2007; 14:2608–2614. | Margarit C, et al. Liver Transpl 2005;11 :1242–1251. | Figueras J, et al. J Am Coll Surg 2000;190 :580–587. | Michel J,  et al. J Hepatol 1997;26: 1274–1280. | Canter 2011 |
|  | Poon RT,  et al. Ann Surg 2007; 245:51–58. | Sangro B, et al. Surgery 1998;124:575–583. |  | Poon RT,  et al. Ann Surg 2007; 245:51–58. | Iwatsuki S,  et al. Ann Surg 1991;214: 221–229. | Rayya F, et al. Transplant Proc. 2008;40:933–935. | Chan SC, et al. Hepatol Int 2011;6:646–656. |
|  | Shah SA, et al. Ann Surg Oncol 2007;14: 2608–2614. | Schwartz ME,  et al. J Am Coll Surg 1995;180:596–603. |  | Shah SA, et al. Ann Surg Oncol 2007; 14:2608–2614. | Lee KK, et al.  J Surg Oncol 2010;101:47–53. | Weimann A, et al. Transplant Proc 1999;31:500–501. | Cillo U, et al. J Surg Oncol 2007;95:213–220. |
|  | Weimann A,  et al. Transplant Proc 1999; 31:500–501. | Tan KC, et al. Br J  Surg 1995;82:253–256. |  |  | Llovet JM,  et al. Hepatology 1999; 30:1434–1440. |  | Closset J, et al. Hepatogastro­- enterology 1999;46: 2914–2918. |
|  |  | Weimann A, et al. Transplant Proc 1999;31:500–501. |  |  | Margarit C,  et al. Liver Transpl 2005; 11:1242–1251. |  | Colella G, et al. Transpl Int 1998;11 Suppl 1:S193–196. |
|  |  | Yamamoto J, et al. Cancer 1999; 86:1151–1158. |  |  | Poon RT, et al. Ann Surg 2007;245:51–58. |  | De Carlis L, et al. J Am Coll Surg 2003; 196:887–897. |
|  |  |  |  |  | Shabahang M,  et al. Ann Surg Oncol 2002;9:881–886. |  | Del Gaudio M, et al. Am J Transplant 2008; 8:1177–1185. |
|  |  |  |  |  | Shah SA, et al. Ann Surg Oncol 2007;14: 2608–2614. |  | Dima 2009 |
|  |  |  |  |  | Tan KC, et al. Br J Surg 1995;82: 253–256. |  | El-Gazzaz G, et al. Transpl Int 2000;13 Suppl 1:S406–409. |
|  |  |  |  |  | Yamamoto J, et al. Cancer 1999;86: 1151–1158. |  | Facciuto ME, et al. HPB 2009;11:398–404. |
|  |  |  |  |  | Zhou J, et al.  J Cancer Res Clin Oncol 2010;136 :1453–1460. |  | Fan ST, et al. Br J Surg 2011;98:1292–300. |
|  |  |  |  |  |  |  | Farinati F, et al. Eur J Gastroenterol Hepatol 2001;13:1217–1224. |
|  |  |  |  |  |  |  | Figueras J, et al. J Am Coll Surg 2000; 190:580–587. |
|  |  |  |  |  |  |  | Fuks 2011 |
|  |  |  |  |  |  |  | Gugenheim 1997 |
|  |  |  |  |  |  |  | Ho 2011 |
|  |  |  |  |  |  |  | Iwatsuki S, et al. Ann Surg 1991;214:221–229. |
|  |  |  |  |  |  |  | Koniaris LG, et al. Ann Surg 2011;254:527–537. |
|  |  |  |  |  |  |  | Kooby 2008 |
|  |  |  |  |  |  |  | Langer 1994 |
|  |  |  |  |  |  |  | Launois 1996 |
|  |  |  |  |  |  |  | Lee KK, et al. J Surg Oncol 2010;101:47–53. |
|  |  |  |  |  |  |  | Llovet JM, et al. Hepatology 1999;30:1434–1440. |
|  |  |  |  |  |  |  | Malek 2010 |
|  |  |  |  |  |  |  | Margarit C, et al. Liver Transpl 2005; 11:1242–1251. |
|  |  |  |  |  |  |  | Mazziotti A, et al. Hepatogastroenterology 1998;45:1281–1287. |
|  |  |  |  |  |  |  | Michel J, et al. J Hepatol 1997;26:1274–1280. |
|  |  |  |  |  |  |  | Moon 2007 |
|  |  |  |  |  |  |  | Obed 2008 |
|  |  |  |  |  |  |  | Perry 2007 |
|  |  |  |  |  |  |  | Philosophe 1998 |
|  |  |  |  |  |  |  | Pichlmayr 1997 |
|  |  |  |  |  |  |  | Pinna 1997 |
|  |  |  |  |  |  |  | Poon RT, et al. Ann Surg 2007;245:51–58. |
|  |  |  |  |  |  |  | Rayya F, et al. Transplant Proc 2008;40:933–935. |
|  |  |  |  |  |  |  | Ringe 1991 |
|  |  |  |  |  |  |  | Ruzzenete 2009 |
|  |  |  |  |  |  |  | Sangro 1998 |
|  |  |  |  |  |  |  | Scatton 2008 |
|  |  |  |  |  |  |  | Shah SA, et al. Ann Surg Oncol 2007; 14:2608–2614. |
|  |  |  |  |  |  |  | Tan 1995 |
|  |  |  |  |  |  |  | Tiao 2005 |
|  |  |  |  |  |  |  | Weimann A, et al. Transplant Proc 1999;31:500–501. |
|  |  |  |  |  |  |  | Yamamoto J, et al. Cancer 1999; 86:1151–1158. |
| Notes: *, only 50 papers could be identified. | | | | | | | |
