# Supplementary material for: Management of hepatocellular carcinoma: an overview of major findings from meta-analyses
Source: Oncotarget. 2016 May 4;7(23):34703–51. doi: 10.18632/oncotarget.9157 (PMC5085185; doi:10.18632/oncotarget.9157)
Supplement: Supplementary file 4 [file oncotarget-07-34703-s004.docx]

| Supplementary Table S13: Overlap of included studies among meta-analyses regarding laparoscopic versus open resection | | | | | | | | | |
| --- | --- | --- | --- | --- | --- | --- | --- | --- | --- |
| **First author** | **Fancellu** | | | **Li** | **Pang** | | **Parks** | | **Twaij** |
| Journal (Year) | J Surg Res (2011) | | | Hepatol Res (2012) | Chinese Journal of Evidence-Based Medicine (2010) | | HPB (Oxford) (2014) | | World J Gastroenterol (2014) |
| Publication type | Full text | | | Full text | Full text | | Full text | | Full text |
| No. Included studies | 9 | | | 10 | 7 | | 15 | | 4 |
| No. Included RCTs | 0 | | | 0 | 0 | | 0 | | 0 |
| Included studies | Aldrighetti L, et al. J Surg Oncol 2010;102:82–86. | | | Aldrighetti L, et al. J Surg Oncol 2010;102:82–86. | Belli G, et al. Surg Endosc 2007;21: 2004–2011. | | Abu Hilial M, et al. J Gastrointest Surg 2011;15:818–823. | | Belli G, et al. Br J Surg 2009;96: 1041–1048. |
|  | Belli G, et al. Br J Surg 2009;96: 1041–1048. | | | Belli G, et al. Br J Surg 2009;96: 1041–1048. | Kaneko H, et al. Am J Surg 2005;189:190–194. | | Alemi F, et al. Am J Surg 2010;200: 591–595. | | Cheung TT, et al. Ann Surg 2013;257: 506–511. |
|  | Endo Y, et al. Surg Laparosc Endosc Percutan Tech 2009;19:e171. | | | Endo Y, et al. Surg Laparosc Endosc Percutan Tech 2009;19:e171. | Lai YY, et al. Guangdong Yi Xue 2006;27:209–210. | | Belli G, et al. Surg Endosc 2007;21: 2004–2011. | | Kanazawa A, et al. Surg Endosc 2013;27: 2592–2597. |
|  | Kaneko H, et al. Am J Surg 2005;189:190–194. | | | Kaneko H, et al. Am J Surg 2005;189:190–194. | Lee KF, et al. Hong Kong Med 2007;13:442–448. | | Cai X, et al. Surg Endosc 2008;22: 2350–2356. | | Truant S, et al. Surg Endosc 2011;25: 3668–3677. |
|  | Lai EC, et al. Arch Surg 2009;144: 143–147. | | | Lai EC, et al. Arch Surg 2009;144: 143–147. | Lu BY, et al. Zhongguo Wei Chuang Wai Ke Za Zhi 2005;5: 536–538. | | Castaing D, Ann Surg 2009;250:849–855. | |  |
|  | Laurent A, et al. Arch Surg 2003;138:763-769. | | | Laurent A, et al. Arch Surg 2003;138:763-769. | Shimada M, et al. Surg Endosc 2001;15:541–544. | | Hu B, et al. World J Gastroenterol 2011;17:4725–4728. | |  |
|  | Sarpel U, et al. Ann Surg Oncol 2009;16:1572–1577. | | | Nguyen KT, et al. Arch Surg 2011;146:348–356. | Wen MJ, et al.  Zhongshan University Xue Bao 2007; 28(3S):132–133. | | Ito K, et al. J Gastrointest Surg 2009;13:2276–2283. | |  |
|  | ShimadaM, et al.  Surg Endosc 2001;15:541–544. | | | Sarpel U, et al. Ann Surg Oncol 2009;16:1572–1577. |  | | Kaneko H, et al. Am J Surg 2005;189: 190–194. | |  |
|  | Tranchart H, et al. Surg Endosc 2010;24:1170–1176. | | | ShimadaM, et al. Surg Endosc 2001;15:541–544. |  | | Lai EC, et al. Arch Surg 2009;144: 143–147. | |  |
|  |  | | | Tranchart H, et al.  Surg Endosc 2010;24:1170–1176. |  | | Lee K, et al. World J Gastroenterol 2011;35:2268–2274. | |  |
|  |  | | |  |  | | Mala T, et al. Surg Endosc 2002;16: 1059–1063. | |  |
|  |  | | |  |  | | Sarpel U, et al. Ann Surg Oncol 2009;16:1572–1577. | |  |
|  |  | | |  |  | | Shimada M, et al. Surg Endosc 2001;15: 541–544. | |  |
|  |  | | |  |  | | Tranchart H, et al. Surg Endosc 2010;24: 1170–1176. | |  |
|  |  | | |  |  | | Truant S, et al. Surg Endosc 2011;25: 3668–3677. | |  |
| Journal (Year) | | World J Gastroenterol (2012) | Chinese Journal of Evidence-Based Medicine (2013) | | | Ann Surg Oncol (2013) | | Dig Dis Sci (2011) | |
| Publication type | | Full text | Full text | | | Full text | | Full text | |
| No. Included studies | | 15 | 13 | | | 15 | | 10 | |
| No. Included RCTs | | 0 | 0 | | | 0 | | 0 | |
| Included studies | | Aldrighetti L, et al.  J Surg Oncol 2010;102:82–86. | Aldrighetti L, et al.  J Surg Oncol 2010;102:82–86. | | | Aldrighetti L, et al.  J Surg Oncol 2010;102:82–86. | | Aldrighetti L, et al.  J Surg Oncol 2010;102: 82–86. | |
|  | | Belli G, et al. Surg Endosc 2007;21: 2004–2011. | Belli G, et al. Surg Endosc 2007;21: 2004–2011. | | | Belli G, et al. Br J Surg 2009;96: 1041–1048. | | Belli G, et al. Surg Endosc 2007;21: 2004–2011. | |
|  | | Endo Y, et al. Surg Laparosc Endosc Percutan Tech 2009;19:e171. | Endo Y, et al. Surg Laparosc Endosc Percutan Tech 2009;19:e171. | | | Endo Y, et al. Surg Laparosc Endosc Percutan Tech 2009;19:e171. | | Endo Y, et al. Surg Laparosc Endosc Percutan Tech 2009;19:e171. | |
|  | | Hu B, et al. World J Gastroenterol 2011;17: 4725–4728. | Hu B, et al. World J Gastroenterol 2011;17: 4725–4728. | | | Hu B, et al. World J Gastroenterol 2011;17: 4725–4728. | | Kaneko H, et al. Am J Surg 2005;189:190–194. | |
|  | | Kaneko H, et al.  Am J Surg 2005;189:190–194. | Kaneko H, et al.  Am J Surg 2005;189:190–194. | | | Kaneko H, et al.  Am J Surg 2005;189:190–194. | | Lai EC, et al. Arch Surg 2009;144: 143–147. | |
|  | | Ker CG, et al. Int J Hepatol 2011;2011:596792. | Kim HH, et al. J Korean Surg Soc2011;80: 412–419. | | | Ker CG, et al. Int J Hepatol 2011;2011:596792. | | Laurent A, et al.  Arch Surg 2003;138: 763–769. | |
|  | | Kim HH, et al. J Korean Surg Soc2011;80: 412–419. | Lai EC, et al. Arch Surg 2009;144: 143–147. | | | Kim HH, et al. J Korean Surg Soc2011;80: 412–419. | | Nguyen KT, et al.  Arch Surg 2011;146: 348–356. | |
|  | | Lai EC, et al. Arch Surg 2009;144: 143–147. | Laurent A, et al.  Arch Surg 2003;138:763–769. | | | Lai EC, et al. Arch Surg 2009;144: 143–147. | | Shimada M, et al.  Surg Endosc 2001;15: 541–544. | |
|  | | Laurent A, et al.  Arch Surg 2003;138:763–769. | Lee KF, et al. World J Surg 2011;35(10): 2268–2274. | | | Laurent A, et al. Arch Surg 2003;138:763–769. | | Sarpel U, et al. Ann Surg Oncol 2009;16: 1572–1577. | |
|  | | Lee K, et al. World J Gastroenterol 2011;35:2268–2274. | Sarpel U, et al. Ann Surg Oncol 2009;16: 1572–1577. | | | Lee KF, et al. World J Surg 2011;35(10): 2268–2274. | | Tranchart H, et al.  Surg Endosc 2010;24:1170–1176. | |
|  | | Nguyen KT, et al.  Arch Surg 2011;146:348–356. | Shimada M, et al.  Surg Endosc 2001;15:541–544. | | | Nguyen KT, et al.  Arch Surg 2011;146:348–356. | |  | |
|  | | Sarpel U, et al. Ann Surg Oncol 2009;16: 1572–1577. | Tranchart H, et al. Surg Endosc 2010;24: 1170–1176. | | | Sarpel U, et al. Ann Surg Oncol 2009;16: 1572–1577. | |  | |
|  | | Shimada M, et al. Surg Endosc 2001;15: 541–544. | Wu Z, et al. Zhonghua Qiang Jing Wai Ke Za Zhi 2010;3(6):507–512. | | | Shimada M, et al. Surg Endosc 2001;15: 541–544. | |  | |
|  | | Tranchart H, et al.  Surg Endosc 2010;24:1170–1176. |  | | | Tranchart H, et al.  Surg Endosc 2010;24:1170–1176. | |  | |
|  | | Truant S, et al. Surg Endosc 2011;25: 3668–3677. |  | | | Truant S, et al. Surg Endosc 2011;25: 3668–3677. | |  | |

| Overlap of included studies among meta-analyses regarding laparoscopic versus open resection (continued) | | | | |
| --- | --- | --- | --- | --- |
| **First author** | **Xiong** | **Yao** | **Yin** | **Zhou** |
| Journal (Year) | World J Gastroenterol (2012) | Chinese Journal of Evidence-Based Medicine (2013) | Ann Surg Oncol (2013) | Dig Dis Sci (2011) |
| Publication type | Full text | Full text | Full text | Full text |
| No. Included studies | 15 | 13 | 15 | 10 |
| No. Included RCTs | 0 | 0 | 0 | 0 |
| Included studies | Aldrighetti L, et al.  J Surg Oncol 2010;102:82–86. | Aldrighetti L, et al.  J Surg Oncol 2010;102:82–86. | Aldrighetti L, et al.  J Surg Oncol 2010;102:82–86. | Aldrighetti L, et al.  J Surg Oncol 2010;102: 82–86. |
|  | Belli G, et al. Surg Endosc 2007;21: 2004–2011. | Belli G, et al. Surg Endosc 2007;21: 2004–2011. | Belli G, et al. Br J Surg 2009;96: 1041–1048. | Belli G, et al. Surg Endosc 2007;21: 2004–2011. |
|  | Endo Y, et al. Surg Laparosc Endosc Percutan Tech 2009;19:e171. | Endo Y, et al. Surg Laparosc Endosc Percutan Tech 2009;19:e171. | Endo Y, et al. Surg Laparosc Endosc Percutan Tech 2009;19:e171. | Endo Y, et al. Surg Laparosc Endosc Percutan Tech 2009;19:e171. |
|  | Hu B, et al. World J Gastroenterol 2011;17: 4725–4728. | Hu B, et al. World J Gastroenterol 2011;17: 4725–4728. | Hu B, et al. World J Gastroenterol 2011;17: 4725–4728. | Kaneko H, et al. Am J Surg 2005;189:190–194. |
|  | Kaneko H, et al.  Am J Surg 2005;189:190–194. | Kaneko H, et al.  Am J Surg 2005;189:190–194. | Kaneko H, et al.  Am J Surg 2005;189:190–194. | Lai EC, et al. Arch Surg 2009;144: 143–147. |
|  | Ker CG, et al. Int J Hepatol 2011;2011:596792. | Kim HH, et al. J Korean Surg Soc2011;80: 412–419. | Ker CG, et al. Int J Hepatol 2011;2011:596792. | Laurent A, et al.  Arch Surg 2003;138: 763–769. |
|  | Kim HH, et al. J Korean Surg Soc2011;80: 412–419. | Lai EC, et al. Arch Surg 2009;144: 143–147. | Kim HH, et al. J Korean Surg Soc2011;80: 412–419. | Nguyen KT, et al.  Arch Surg 2011;146: 348–356. |
|  | Lai EC, et al. Arch Surg 2009;144: 143–147. | Laurent A, et al.  Arch Surg 2003;138:763–769. | Lai EC, et al. Arch Surg 2009;144: 143–147. | Shimada M, et al.  Surg Endosc 2001;15: 541–544. |
|  | Laurent A, et al.  Arch Surg 2003;138:763–769. | Lee KF, et al. World J Surg 2011;35(10): 2268–2274. | Laurent A, et al. Arch Surg 2003;138:763–769. | Sarpel U, et al. Ann Surg Oncol 2009;16: 1572–1577. |
|  | Lee K, et al. World J Gastroenterol 2011;35:2268–2274. | Sarpel U, et al. Ann Surg Oncol 2009;16: 1572–1577. | Lee KF, et al. World J Surg 2011;35(10): 2268–2274. | Tranchart H, et al.  Surg Endosc 2010;24:1170–1176. |
|  | Nguyen KT, et al.  Arch Surg 2011;146:348–356. | Shimada M, et al.  Surg Endosc 2001;15:541–544. | Nguyen KT, et al.  Arch Surg 2011;146:348–356. |  |
|  | Sarpel U, et al. Ann Surg Oncol 2009;16: 1572–1577. | Tranchart H, et al. Surg Endosc 2010;24: 1170–1176. | Sarpel U, et al. Ann Surg Oncol 2009;16: 1572–1577. |  |
|  | Shimada M, et al. Surg Endosc 2001;15: 541–544. | Wu Z, et al. Zhonghua Qiang Jing Wai Ke Za Zhi 2010;3(6):507–512. | Shimada M, et al. Surg Endosc 2001;15: 541–544. |  |
|  | Tranchart H, et al.  Surg Endosc 2010;24:1170–1176. |  | Tranchart H, et al.  Surg Endosc 2010;24:1170–1176. |  |
|  | Truant S, et al. Surg Endosc 2011;25: 3668–3677. |  | Truant S, et al. Surg Endosc 2011;25: 3668–3677. |  |
