# Supplementary material for: Management of hepatocellular carcinoma: an overview of major findings from meta-analyses
Source: Oncotarget. 2016 May 4;7(23):34703–51. doi: 10.18632/oncotarget.9157 (PMC5085185; doi:10.18632/oncotarget.9157)
Supplement: Supplementary file 5 [file oncotarget-07-34703-s005.docx]

| Supplementary Table S14: Overlap of included studies among meta-analyses regarding anatomic versus nonanatomic resection | | | | | | |
| --- | --- | --- | --- | --- | --- | --- |
| **First author** | **Chen** | **Cucchetti** | **Li** | **Tang** | **Ye** | **Zhou** |
| Journal (Year) | Dig Dis Sci (2011) | Ann Surg Oncol (2012) | Hepato-gastroenterology (2011) | Hepato-gastroenterology (2013) | Asian Pac J Cancer Prev (2012) | Langenbecks Arch Surg (2011) |
| Publication type | Full text | Full text | Full text | Full text | Full text | Full text |
| No. Included studies | 9 | 18 | 4 | 12 | 11 | 16 |
| No. Included RCTs | 0 | 0 | 0 | 0 | 0 | 0 |
| Included studies | Capussotti L,  et al. Eur J Surg Oncol 2005;31: 986–993. | Capussotti L, et al. Eur J Surg Oncol 2005;31: 986–993. | Cho YB, et al. Hepatogastroenterology 2007;54:1766–1769. | Cho YB, et al. Hepatogastroenterology 2007;54:1766–1769. | Arii S, et al. Oncology 2010;78(Suppl 1):125–130. | Cho YB, et al. Hepatogastroenterology 2007;54:1766–1769. |
|  | Hasegawa K, et al. Ann Surg 2005;242: 252–259. | Cho YB, et al. Hepatogastroenterology 2007;54:1766–1769. | Dahiya D, et al. Surgery 2010;147:676–685. | Eltawil KM, et al. World J Surg Oncol 2010;8:43 | Chang Moo Kang 2010 | Eltawil KM, et al. World J Surg Oncol 2010;8:43 |
|  | Kaibori M, et al. Surgery 2006;139: 385–394. | Dahiya D, et al. Surgery 2010;147:676–685. | Suh KS, et al. J Hepatobiliary Pancreat Surg 2005;12(5): 365–370. | Hasegawa K, et al. Ann Surg 2005;242:252–259. | Kaibori M, et al. Surgery 2006;139: 385–394. | Kaibori M, et al. Surgery 2006;139: 385–394. |
|  | Kaneko H, et al. Am J Surg 2005;189: 190–194. | Eguchi S, et al. Surgery 2008; 143:469–475. | Ueno S, et al. J Hepatobiliary Pancreat Surg 2008;15:493–500. | Kaibori M, et al. Surgery 2006;139:385–394. | Karim 2010 | Kamiyama T, et al. J Surg Oncol 2010;101:54–60. |
|  | Regimbeau JM, et al. Surgery 2002;131: 311–317. | Hasegawa K, et al. Ann Surg 2005;242: 252–259. |  | Kang CM, et al. J Surg Res 2010;160:81–89. | Kobayashi A, et al. J Hepatobiliary Pancreat Surg 2008;15: 515–521. | Kang CM, et al. J Surg Res 2010;160:81–89. |
|  | Tanaka K, et al. Surgery 2008;143: 607–615. | Kaibori M, et al. Surgery 2006;139: 385–394. |  | Nanashima A, et al.  Acta Chir Belg 2008;108:532–537. | Nanashima A, et al. Acta Chir Belg 2008;108: 532–537. | Kobayashi A, et al. J Hepatobiliary Pancreat Surg 2008;15:515–521. |
|  | Ueno S, et al. J Hepatobiliary Pancreat Surg 2008;15: 493–500. | Kamiyama T, et al. J Surg Oncol 2010;101: 54–60. |  | Tanaka K, et al. Surgery 2008;143:607–615. | Tanaka K, et al. Surgery 2008;143: 607–615. | Hasegawa K, et al. Ann Surg 2005;242: 252–259. |
|  | Wakai T, et al. Ann Surg Oncol. 2007;14: 1356–1365. | Kang CM, et al. J Surg Res 2010;160:81–89. |  | Ueno S, et al. J Hepatobiliary Pancreat Surg 2008;15:493–500. | Tanaka S, et al. J Am Coll Surg 2009;208(3):368–374. | Nanashima A, et al.  Acta Chir Belg 2008;108:532–537. |
|  | Yamashita Y, et al. J Am Coll Surg 2007;205: 19–26. | Kobayashi A, et al. J Hepatobiliary Pancreat Surg 2008;15:515–521. |  | Wakai T, et al. Ann Surg Oncol 2007;14: 1356–1365. | Wakai T, et al. Ann Surg Oncol 2007;14: 1356–1365. | Regimbeau JM, et al. Surgery 2002;131: 311–317. |
|  | Ziparo V, et al. Eur J Surg Oncol 2002;28: 723–728. | Nanashima A, et al. Acta Chir Belg 2008;108:532–537. |  | Wang K, et al. Zhonghua Zhong Liu Lin Chuang 2007;6(34):330–333. | Regimbeau JM, et al. Surgery 2002;131: 311–317. | Tanaka K, et al. Surgery 2008;143:607–615. |
|  |  | Regimbeau JM, et al. Surgery 2002;131: 311–317. |  | Wang JG, et al. Guangxi Medical Journal 2010;6:659–662. | Yamazaki O, et al. J Hepatobiliary Pancreat Sci 2010;17: 349–358. | Tanaka S, et al. J Am Coll Surg 2009;208(3):368–374. |
|  |  | Tanaka K, et al. Surgery 2008;143:607–615. |  | Yamashita Y, et al. J Am Coll Surg 2007;205: 19–26. |  | Ueno S, et al. J Hepatobiliary Pancreat Surg 2008;15:493–500. |
|  |  | Ueno S, et al. J Hepatobiliary Pancreat Surg 2008;15:493–500. |  |  |  | Wakai T, et al. Ann Surg Oncol 2007;14: 1356–1365. |
|  |  | Wakai T, et al. Ann Surg Oncol 2007;14: 1356–1365. |  |  |  | Yamamoto M, et al. Surgery 2001;130: 443–448. |
|  |  | Yamamoto M, et al. Surgery 2001;130: 443–448. |  |  |  | Yamashita Y, et al. J Am Coll Surg 2007;205: 19–26. |
|  |  | Yamashita Y, et al. J Am Coll Surg 2007;205: 19–26. |  |  |  | Yamazaki O, et al. J Hepatobiliary Pancreat Sci 2010;17:349–358. |
|  |  | Yamazaki O, et al. J Hepatobiliary Pancreat Sci 2010;17:349–358. |  |  |  |  |
|  |  | Ziparo V, et al. Eur J Surg Oncol 2002;28:723–728. |  |  |  |  |
