# Supplementary material for: Management of hepatocellular carcinoma: an overview of major findings from meta-analyses
Source: Oncotarget. 2016 May 4;7(23):34703–51. doi: 10.18632/oncotarget.9157 (PMC5085185; doi:10.18632/oncotarget.9157)
Supplement: Supplementary file 6 [file oncotarget-07-34703-s006.docx]

| Supplementary Table S19: Overlap of included studies among meta-analyses regarding surgical resection plus adjuvant chemotherapy | | | |
| --- | --- | --- | --- |
| **First author** | **Ono** | **Wang** | **Zheng** |
| Journal (Year) | Cancer (2001) | Can J Gastroenterol (2013) | Int J Cancer (2014) |
| Publication type | Full text | Full text | Full text |
| No. Included studies | 3 | 8 | 48 |
| No. Included RCTs | 3 | 8 | 13 |
| Included studies | Not reported | Hasegawa K, et al. Hepatology 2006;44:891–5. | Arii S 1994 |
|  |  | Izumi R, et al. Hepatology 1994;20:295–301. | Asahara T 1999 |
|  |  | Lai EC, et al. Arch Surg 1998;133:183–8. | Chau TG 2006 |
|  |  | Ono T, et al. Semin Oncol 1997;24:S6–18-S6–25. | Chen J 2011 |
|  |  | Tanaka S, et al. Hepatogastroenterology 2005;52: 862–5. | Cheng SQ 2005 |
|  |  | Ueno S, et al. Cancer 1999;86:248–54. | Fan J 2001 |
|  |  | Xia Y, et al. Ann Surg Oncol 2010;17:3137–44. | Fan J 2005 |
|  |  | Yamamoto M, et al. Br J Surg 1996;83:336–40. | Ge L 2008 |
|  |  |  | Harada A 1992 |
|  |  |  | Hasegawa K 2006 |
|  |  |  | He YZ(a) 2001 |
|  |  |  | He YZ(b) 2001 |
|  |  |  | He YZ(c) 2001 |
|  |  |  | Huang YH 2000 |
|  |  |  | Huang ZJ 2009 |
|  |  |  | Itamoto T 1992 |
|  |  |  | Izumi R 1994 |
|  |  |  | Kim DY 2011 |
|  |  |  | Lai EC 1998 |
|  |  |  | Lei ZM 2003 |
|  |  |  | Li HH(a) 2000 |
|  |  |  | Li HH(b) 2000 |
|  |  |  | Li Q(a) 2006 |
|  |  |  | Li Q(b) 2006 |
|  |  |  | Liao JQ 2011 |
|  |  |  | Liu YB 2004 |
|  |  |  | Misawa K 1993 |
|  |  |  | Nagabuchi E 1991 |
|  |  |  | Ni C 2005 |
|  |  |  | Niguma T 2005 |
|  |  |  | Nonami T 1991 |
|  |  |  | Peng BG 2006 |
|  |  |  | Peng BG 2009 |
|  |  |  | Ren ZG 2004 |
|  |  |  | Shi Y 2003 |
|  |  |  | Shimoda M 2001 |
|  |  |  | Takenaka K(a) 1995 |
|  |  |  | Takenaka K(b) 1995 |
|  |  |  | Tanaka K 1999 |
|  |  |  | Tanaka S 2005 |
|  |  |  | Togo S 2005 |
|  |  |  | Ueno M 2011 |
|  |  |  | Ueno S 1999 |
|  |  |  | Une Y 1995 |
|  |  |  | Wang TH 2008 |
|  |  |  | Wu HZ 2001 |
|  |  |  | Xia Y 2010 |
|  |  |  | Yamamoto M 1996 |
|  |  |  | Yu ZP 2009 |
|  |  |  | Zhang GQ 2008 |
|  |  |  | Zhang Q 2011 |
|  |  |  | Zhong C 2009 |
