# Supplementary material for: Management of hepatocellular carcinoma: an overview of major findings from meta-analyses
Source: Oncotarget. 2016 May 4;7(23):34703–51. doi: 10.18632/oncotarget.9157 (PMC5085185; doi:10.18632/oncotarget.9157)
Supplement: Supplementary file 7 [file oncotarget-07-34703-s007.docx]

| Supplementary Table S21: Overlap of included studies among meta-analyses regarding RFA versus surgical resection | | | | | | | | | | | | | | | | | | | |
| --- | --- | --- | --- | --- | --- | --- | --- | --- | --- | --- | --- | --- | --- | --- | --- | --- | --- | --- | --- |
| **First author** | | **Cai** | | **Chen** | | | **Cucchetti** | | | **Duan** | | | **Feng** | | | | **Fu** | | |
| Journal (Year) | | HPB (2013) | | Zhonghua Wai Ke Za Zhi (2008) | | | J Hepatol (2013) | | | World J Surg Oncol (2013) | | | J Cancer Res Clin Oncol (2014) | | | | Hepato-gastroenterology (2014) | | |
| Publication type | | Abstract | | Full text | | | Full text | | | Full text | | | Full text | | | | Full text | | |
| No. Included studies | | 5 | | 6 | | | 17 | | | 12 | | | 23 | | | | 5 | | |
| No. Included RCTs | | Not reported | | 1 | | | Not reported | | | 2 | | | 3 | | | | 5 | | |
| Included studies | | Not reported | | Chen MS, et al.  Ann Surg 2006;243: 321–328. | | | Abu-Hilal M, et al. J Gastrointest Surg 2008;12:1521–1526. | | | Chen MS, et al. Ann Surg 2006;243:321–328. | | | Abu-Hilal M, et al. J Gastrointest Surg 2008;12:1521–1526. | | | | Chen MS, et al. Zhonghua Yi Xue Za Zhi 2005;85(2): 80–83. | | |
|  | |  | | Cho CM, et al. Korean J Hepatol 2005;11:59–71. | | | Chen MS, et al. Ann Surg 2006;243:321–328. | | | Cho CM, et al. Korean J Hepatol 2005;11:59–71. | | | Chen MS, et al. Ann Surg 2006;243:321–328. | | | | Chen MS, et al. Ann Surg 2006;243: 321–328. | | |
|  | |  | | Gao W, et al. Chin J Med Imaging Technol 2007;23: 254–257. | | | Cho CM, et al. Korean J Hepatol 2005;11:59–71. | | | Guglielmi A, et al. J Gastrointest Surg 2008;12:192–198. | | | Desiderio J, et al. Langenbeck’s Arch Surg 2013;398:55–62. | | | | Feng K, et al. J Hepatol 2012;57: 794–802. | | |
|  | |  | | Hong SN, et al. J Clin Gastroenterol 2005;39: 247–252. | | | Guglielmi A, et al. J Gastrointest Surg 2008;12:192–198. | | | Guo WX, et al. World J Surg 2013, 37(3):602–607 | | | Feng K, et al. J Hepatol 2012;57:794–802. | | | | Huang J, et al. Ann Surg 2010;252: 903–912. | | |
|  | |  | | Montorsi M, et al. J Gastrointest Surg 2005;9:62–67. | | | Hasegawa K, et al. J Hepatol 2008;49: 589–594. | | | Hasegawa K, et al. J Hepatol 2008;49:589–594. | | | Guglielmi A, et al. J Gastrointest Surg 2008;12:192–198. | | | | Lü MD, et al. Zhonghua Yi Xue Za Zhi 2006;86(12): 801–805. | | |
|  | |  | | Vivarelli M, et al. Ann Surg 2004;240: 102–107. | | | Hiraoka A, et al. Hepatogastroenterology 2008;55:2171–2174. | | | Hiraoka A, et al. Hepatogastroenterology 2008;55:2171–2174. | | | Guo WX, et al. World J Surg 2013, 37(3):602–607. | | | |  | | |
|  | |  | |  | | | Hong SN, et al. J Clin Gastroenterol 2005;39:247–252. | | | Hong SN, et al. J Clin Gastroenterol 2005;39:247–252. | | | Hasegawa K, et al. J Hepatol 2008;49:589–594. | | | |  | | |
|  | |  | |  | | | Huang J, et al. Ann Surg 2010;252:903–912. | | | Huang J, et al. Ann Surg 2010;252:903–912. | | | Hiraoka A, et al. Hepatogastroenterology 2008;55:2171–2174. | | | |  | | |
|  | |  | |  | | | Lupo L, et al. HPB (Oxford) 2007;9: 429–434. | | | Lü MD, et al. Zhonghua Yi Xue Za Zhi 2006;86(12):801–805. | | | Huang J, et al. Ann Surg 2010;252:903–912. | | | |  | | |
|  | |  | |  | | | Montorsi M, et al. J Gastrointest Surg 2005;9:62–67. | | | Lupo L, et al. HPB (Oxford) 2007;9:429–434. | | | Hung HH, et al. Clin Gastroenterol Hepatol 2011;9:79–86. | | | |  | | |
|  | |  | |  | | | Nishikawa H, et al. BMC Gastroenterol 2011;11:143. | | | Ueno S, et al. J Hepatobiliary Pancreat Surg 2009;16:359–366. | | | Ikeda K, et al. Liver Int 2011;31:692–699. | | | |  | | |
|  | |  | |  | | | Ogihara M, et al. HPB (Oxford) 2005;7: 214–221. | | | Vivarelli M, et al. Ann Surg 2004;240:102–107. | | | Imai K, et al. Hepatol Res 2012;43:853–864. | | | |  | | |
|  | |  | |  | | | Peng ZW, et al. Radiology 2012;262:1022–1033. | | |  | | | Kong WT, et al. Chin-Ger J Clin Oncol 2011;10(7): 399–405. | | | |  | | |
|  | |  | |  | | | Takayama T, et al. J Hepatobiliary Pancreat Sci 2010;17:422–424. | | |  | | | Lupo L, et al. HPB (Oxford) 2007;9:429–434. | | | |  | | |
|  | |  | |  | | | Ueno S, et al. J Hepatobiliary Pancreat Surg 2009;16:359–366. | | |  | | | Nishikawa H, et al. BMC Gastroenterol 2011;11:143. | | | |  | | |
|  | |  | |  | | | Vivarelli M, et al. Ann Surg 2004;240:102–107. | | |  | | | Peng ZW, et al. Radiology 2012;262:1022–1033. | | | |  | | |
|  | |  | |  | | | Wang JH, et al. J Hepatol 2012;56:412–418. | | |  | | | Pompili M, et al. J Hepatol 2013;59:89–97. | | | |  | | |
|  | |  | |  | | |  | | |  | | | Sung NH, et al. J Clin Gastroenterol 2005;39: 247–252. | | | |  | | |
|  | |  | |  | | |  | | |  | | | Tohme S, et al. HPB 2013;15:210–217. | | | |  | | |
|  | |  | |  | | |  | | |  | | | Ueno S, et al. J Hepatobiliary Pancreat Surg 2009;16:359–366. | | | |  | | |
|  | |  | |  | | |  | | |  | | | Wang JH, et al. J Hepatol 2012;56:412–418. | | | |  | | |
|  | |  | |  | | |  | | |  | | | Wong KM, et al. Indian J Gastroenterol 2012;32: 253–257. | | | |  | | |
|  | |  | |  | | |  | | |  | | | Yun WK, et al. Hepatol Int 2011;5:722–729. | | | |  | | |
| Overlap of included studies among meta-analyses regarding RFA versus surgical resection (continued) | | | | | | | | | | | | | | | | | |  |  |
| **First author** | | | **Hu** | | **Li** | | | **Liu** | | | | **Liu** | | | **Ni** | | **Qi** |  |  |
| Journal (Year) | | | HPB (2013) | | J Gastroenterol Hepatol (2012) | | | World J Gastroenterol (2010) | | | | Surg Laparosc Endosc Percutan Tech (2010) | | | J Cancer Res Clin Oncol (2013) | | J Clin Gastroenterol (2014) |  |  |
| Publication type | | | Abstract | | Full text | | | Full text | | | | Full text | | | Full text | | Full text |  |  |
| No. Included studies | | | 18 | | 6 | | | 10 | | | | 8 | | | 10 | | 3 |  |  |
| No. Included RCTs | | | 4 | | 2 | | | 0 | | | | 8 | | | 6 | | 3 |  |  |
| Included studies | | | Not reported | | Abu-Hilal M, et al. J Gastrointest Surg 2008;12:1521–1526. | | | Cho CM, et al. Korean J Hepatol 2005;11:59–71. | | | | Abu-Hilal M, et al. J Gastrointest Surg 2008;12:1521–1526. | | | Cho YB, et al. J Gastroenterol Hepatol 2007;22: 1643–1649. | | Chen MS, et al. Ann Surg 2006;243: 321–328. |  |  |
|  | | |  | | Chen MS, et al. Ann Surg 2006;243:321–328. | | | Gao W, et al. Chin J Med Imaging Technol 2007;23:254–257. | | | | Guglielmi A, et al. J Gastrointest Surg 2008;12:192–198. | | | Chen MS, et al. Zhonghua Yi Xue Za Zhi 2005;85(2):80–83. | | Feng K, et al. J Hepatol 2012;57: 794–802. |  |  |
|  | | |  | | Hiraoka A, et al. Hepatogastroenterology 2008;55:2171–2174. | | | Guglielmi A, et al. J Gastrointest Surg 2008;12:192–198. | | | | Liang HH, et al. Ann Surg Oncol 2008;15:3484–3493. | | | Chen MS, et al. Ann Surg 2006;243:321–328. | | Huang J, et al. Ann Surg 2010;252: 903–912. |  |  |
|  | | |  | | Huang J, et al. Ann Surg 2010;252:903–912. | | | Hong SN, et al. J Clin Gastroenterol 2005;39:247–252. | | | | Ogihara M, et al. HPB (Oxford) 2005;7: 214–221. | | | Feng K, et al.  J Hepatol 2012;57:794–802. | |  |  |  |
|  | | |  | | Lupo L, et al. HPB (Oxford) 2007;9: 429–434. | | | Montorsi M, et al. J Gastrointest Surg 2005;9:62–67. | | | | Ueno S, et al. J Hepatobiliary Pancreat Surg 2009;16:359–366. | | | Hasegawa K, et al. J Hepatol 2008;49:589–594. | |  |  |  |
|  | | |  | | Santambrogio R, et al. Ann Surg Oncol 2009;16:3289–3298. | | | Peng ZW, et al. Radiology 2012;262:1022–1033. | | | | Vivarelli M, et al. Ann Surg 2004;240: 102–107. | | | Hasegawa K, et al. J Hepatol 2013;58:724–729. | |  |  |  |
|  | | |  | |  | | | Vivarelli M, et al. Ann Surg 2004;240: 102–107. | | | | Wong J, et al. Asian J Surg 2009;32: 13–20. | | | Huang GT, et al. Ann Surg 2005;242:36–42. | |  |  |  |
|  | | |  | |  | | | Wakai T, et al. World J Gastroenterol 2006;12:546–552. | | | | Yamakado K, et al. Radiology 2008;247:260–266. | | | Huang J, et al. Ann Surg 2010;252:903–912. | |  |  |  |
|  | | |  | |  | | | Zhang LQ, et al. Disan Junyi Daxue Xuebao 2007;29: 457–459. | | | |  | | | Lü MD, et al. Zhonghua Yi Xue Za Zhi 2006;86(12): 801–805. | |  |  |  |
|  | | |  | |  | | | Zhou T, et al. Gandan Waike Zazhi 2007;15:424–427. | | | |  | | | Tohme S, et al. HPB 2013;15: 210–217. | |  |  |  |
| Overlap of included studies among meta-analyses regarding RFA versus surgical resection (continued) | | | | | | | | | | | | | | | | | | |  |
| **First author** | **Sun** | | | | | **Wang** | | | **Weis** | | **Xu** | | | **Zhou** | | **Zhou** | | |  |
| Journal (Year) | World Chinese J Digestology (2011) | | | | | PLoS One (2014) | | | Cochrane Database Syst Rev (2013) | | World J Surg Oncol (2012) | | | Zhonghua Wai Ke Za Zhi (2011) | | BMC Gastroenterol (2010) | | |  |
| Publication type | Full text | | | | | Full text | | | Full text | | Full text | | | Full text | | Full text | | |  |
| No. Included studies | 11 | | | | | 28 | | | 3 | | 13 | | | 4 | | 10 | | |  |
| No. Included RCTs | 2 | | | | | 3 | | | 3 | | 2 | | | 4 | | 1 | | |  |
| Included studies | Chen MS, et al. Ann Surg 2006;243: 321–328. | | | | | Abu-Hilal M, et al. J Gastrointest Surg 2008;12:1521–1526. | | | Chen MS, et al. Ann Surg 2006;243: 321–328. | | Abu-Hilal M, et al. J Gastrointest Surg 2008;12:1521–1526. | | | Chen MS, et al. Ann Surg 2006;243: 321–328. | | Abu-Hilal M, et al. J Gastrointest Surg 2008;12:1521–1526. | | |  |
|  | Cho CM, et al. Korean J Hepatol 2005;11: 59–71. | | | | | Bu XY, et al. Chin Arch Gen Surg (Electronic Edition) 2009;3(2): 127–131. | | | Feng K, et al. J Hepatol 2012;57: 794–802. | | Chen MS, et al. Ann Surg 2006;243: 321–328. | | | Huang J, et al. Ann Surg 2010;252: 903–912. | | Chen MS, et al. Ann Surg 2006;243: 321–328. | | |  |
|  | Guglielmi A, et al. J Gastrointest Surg 2008;12:192–198. | | | | | Chen MS, et al. Ann Surg 2006;243: 321–328. | | | Huang J, et al. Ann Surg 2010;252: 903–912. | | Guglielmi A, et al. J Gastrointest Surg 2008;12:192–198. | | | Lü MD, et al. Zhonghua Yi Xue Za Zhi 2006;86(12): 801–805. | | Cho CM, et al. Korean J Hepatol 2005;11:59–71. | | |  |
|  | Guo WX, et al. World J Surg 2010;34: 2671–2676. | | | | | Cho CM, et al. Korean J Hepatol 2005;11: 59–71. | | |  | | Hiraoka A, et al. Hepatogastroenterology 2008;55:2171–2174. | | | Vivarelli M, et al. Ann Surg 2004;240: 102–107. | | Guglielmi A, et al. J Gastrointest Surg 2008;12:192–198. | | |  |
|  | Hiraoka A, et al. Hepatogastroenterology 2008;55:2171–2174. | | | | | Feng K, et al. J Hepatol 2012;57: 794–802. | | |  | | Hong SN, et al. J Clin Gastroenterol 2005;39:247–252. | | |  | | Hiraoka A, et al. Hepatogastroenterology 2008;55:2171–2174. | | |  |
|  | Huang J, et al. Ann Surg 2010;252: 903–912. | | | | | Fu J, et al. Journal of Jilin University (Medicine Edition) 2011;37(4):733–737. | | |  | | Huang J, et al. Ann Surg 2010;252: 903–912. | | |  | | Hong SN, et al. J Clin Gastroenterol 2005;39:247–252. | | |  |
|  | Lü MD, et al. Zhonghua Yi Xue Za Zhi 2006;86(12): 801–805. | | | | | Gao W, et al. Chin J Med Imaging Technol 2007;23:254–257. | | |  | | Hung HH, et al. Clin Gastroenterol Hepatol 2011;9:79–86. | | |  | | Lupo L, et al. HPB (Oxford) 2007;9: 429–434. | | |  |
|  | Ogihara M, et al. HPB (Oxford) 2005;7: 214–221. | | | | | Guglielmi A, et al. J Gastrointest Surg 2008;12:192–198. | | |  | | Nanashima A, et al. J Surg Oncol 2010;101:481–485. | | |  | | Montorsi M, et al. J Gastrointest Surg 2005;9:62–67. | | |  |
|  | Peng ZW, et al. Radiology 2012;262:1022–1033. | | | | | Guo WX, et al. World J Surg 2010;34: 2671–2676. | | |  | | Nishikawa H, et al. BMC Gastroenterol 2011;11:143. | | |  | | Santambrogio R, et al. Ann Surg Oncol 2009;16:3289–3298. | | |  |
|  | Ueno S, et al. J Hepatobiliary Pancreat Surg 2009;16:359–366. | | | | | Hasegawa K, et al. J Hepatol 2008;49: 589–594. | | |  | | Takahashi S, et al. Dig Dis 2007;25:303–309. | | |  | | Vivarelli M, et al. Ann Surg 2004;240: 102–107. | | |  |
|  | Vivarelli M, et al. Ann Surg 2004;240: 102–107. | | | | | Hiraoka A, et al. Hepatogastroenterology 2008;55:2171–2174. | | |  | | Tashiro H, et al. J Surg Oncol 2011;104:3–9. | | |  | |  | | |  |
|  |  | | | | | Hong SN, et al. J Clin Gastroenterol 2005;39:247–252. | | |  | | Ueno S, et al. J Hepatobiliary Pancreat Surg 2009;16:359–366. | | |  | |  | | |  |
|  |  | | | | | Huang J, et al. Ann Surg 2010;252: 903–912. | | |  | | Vivarelli M, et al. Ann Surg 2004;240: 102–107. | | |  | |  | | |  |
|  |  | | | | | Huang J, et al. J Gastrointest Surg 2011;15:311–320. | | |  | |  | | |  | |  | | |  |
|  |  | | | | | Hung HH, et al. Clin Gastroenterol Hepatol 2011;9:79–86. | | |  | |  | | |  | |  | | |  |
|  |  | | | | | Ikeda K, et al. Liver Int 2011;31:692–699. | | |  | |  | | |  | |  | | |  |
|  |  | | | | | Kobayashi M, et al. Cancer 2009;115: 571–580. | | |  | |  | | |  | |  | | |  |
|  |  | | | | | Kong WT, et al. Chin-Ger J Clin Oncol 2011;10(7):399–405. | | |  | |  | | |  | |  | | |  |
|  |  | | | | | Liu H, et al. Pracitical Clinical Medicine 2011;12(11):16–18. | | |  | |  | | |  | |  | | |  |
|  |  | | | | | Lupo L, et al. HPB (Oxford) 2007;9: 429–434. | | |  | |  | | |  | |  | | |  |
|  |  | | | | | Montorsi M, et al. J Gastrointest Surg 2005;9:62–67. | | |  | |  | | |  | |  | | |  |
|  |  | | | | | Nishikawa H, et al. BMC Gastroenterol 2011;11:143. | | |  | |  | | |  | |  | | |  |
|  |  | | | | | Peng ZW, et al. Radiology 2012;262:1022–1033. | | |  | |  | | |  | |  | | |  |
|  |  | | | | | Santambrogio R, et al. Ann Surg Oncol 2009;16:3289–3298. | | |  | |  | | |  | |  | | |  |
|  |  | | | | | Ueno S, et al. J Hepatobiliary Pancreat Surg 2009;16:359–366. | | |  | |  | | |  | |  | | |  |
|  |  | | | | | Vivarelli M, et al. Ann Surg 2004;240: 102–107. | | |  | |  | | |  | |  | | |  |
|  |  | | | | | Wang JH, et al. J Hepatol 2012;56: 412–418. | | |  | |  | | |  | |  | | |  |
|  |  | | | | | Zhou T, et al. Gandan Waike Zazhi 2007;15:424–427. | | |  | |  | | |  | |  | | |  |
