# Supplementary material for: Management of hepatocellular carcinoma: an overview of major findings from meta-analyses
Source: Oncotarget. 2016 May 4;7(23):34703–51. doi: 10.18632/oncotarget.9157 (PMC5085185; doi:10.18632/oncotarget.9157)
Supplement: Supplementary file 8 [file oncotarget-07-34703-s008.docx]

| Supplementary Table S23: Overlap of included studies among meta-analyses regarding RFA versus PEI or PAI | | | | | | | | |  |
| --- | --- | --- | --- | --- | --- | --- | --- | --- | --- |
| **First author** | | **Bouza** | | **Cho** | | **Dong** | | **Germani** |  |
| Journal (Year) | | BMC Gastroenterol (2009) | | Hepatology (2009) | | World J Gastroenterol (2014) | | J Hepatol (2010) |  |
| Publication type | | Full text | | Full text | | Full text | | Full text |  |
| No. Included studies | | 6 | | 4 | | 5 | | 5 |  |
| No. Included RCTs | | 6 | | 4 | | 5 | | 5 |  |
| Included studies | | Brunello F, et al. Scand J Gastroenterol 2008;43:727–735. | | Brunello F, et al. Scand J Gastroenterol 2008;43:727–735. | | Brunello F, et al. Scand J Gastroenterol 2008;43:727–735. | | Brunello F, et al. Scand J Gastroenterol 2008;43:727–735. |  |
|  | | Lencioni RA, et al. Radiology 2003;228: 235–240. | | Lin SM, et al. Gastroenterology 2004;127:1714–1723. | | Lencioni RA, et al. Radiology 2003;228:235–240. | | Lencioni RA, et al. Radiology 2003;228: 235–240. |  |
|  | | Lin SM, et al. Gastroenterology 2004;127:1714–1723. | | Lin SM, et al. Gut 2005;54:1151–1156. | | Lin SM, et al. Gastroenterology 2004;127: 1714–1723. | | Lin SM, et al. Gastroenterology 2004;127: 1714–1723. |  |
|  | | Lin SM, et al. Gut 2005;54:1151–1156. | | Shiina S, et al. Gastroenterology 2005;129:122–130. | | Lin SM, et al. Gut 2005;54:1151–1156. | | Livraghi T, et al. Radiology 1999;210:655–661. |  |
|  | | Livraghi T, et al. Radiology 1999;210: 655–661. | |  | | Shiina S, et al. Gastroenterology 2005;129:122–130. | | Shiina S, et al. Gastroenterology 2005;129:122–130. |  |
|  | | Shiina S, et al. Gastroenterology 2005;129:122–130. | |  | |  | |  |  |
| Overlap of included studies among meta-analyses regarding RFA versus PEI or PAI (continued) | | | | | | | | | |
| **First author** | **Orlando** | | **Shen** | | **Weis** | | **Xu** | | |
| Journal (Year) | Am J Gastroenterol (2009) | | J Gastroenterol Hepatol (2013) | | Cochrane Database Syst Rev (2013) | | Eur J Med Res (2014) | | |
| Publication type | Full text | | Full text | | Full text | | Full text | | |
| No. Included studies | 5 | | 5 | | 6 | | 6 | | |
| No. Included RCTs | 5 | | 4 | | 6 | | 6 | | |
| Included studies | Brunello F, et al. Scand J Gastroenterol 2008;43: 727–735. | | Brunello F, et al. Scand J Gastroenterol 2008;43:727–735. | | Brunello F, et al. Scand J Gastroenterol 2008;43:727–735. | | Brunello F, et al. Scand J Gastroenterol 2008;43:727–735. | | |
|  | Lencioni RA, et al. Radiology 2003;228: 235–240. | | Giorgio A, et al. Anticancer Res. 2011;31:2291–5. | | Giorgio A, et al. Anticancer Res. 2011;31:2291–5. | | Giorgio A, et al. Anticancer Res. 2011;31:2291–5. | | |
|  | Lin SM, et al. Gastroenterology 2004;127:1714–1723. | | Lencioni RA, et al. Radiology 2003;228:235–240. | | Lencioni RA, et al. Radiology 2003;228:235–240. | | Lencioni RA, et al. Radiology 2003;228:235–240. | | |
|  | Livraghi T, et al. Radiology 1999;210:655–661. | | Lin SM, et al. Gut 2005;54:1151–1156. | | Lin SM, et al. Gastroenterology 2004;127:1714–1723. | | Lin SM, et al. Gastroenterology 2004;127:1714–1723. | | |
|  | Shiina S, et al. Gastroenterology 2005;129:122–130. | | Shiina S, et al. Gastroenterology 2005;129:122–130. | | Lin SM, et al. Gut 2005;54:1151–1156. | | Lin SM, et al. Gut 2005;54:1151–1156. | | |
|  |  | |  | | Shiina S, et al. Gastroenterology 2005;129:122–130. | | Shiina S, et al. Gastroenterology 2005;129:122–130. | | |
