# Supplementary material for: Management of hepatocellular carcinoma: an overview of major findings from meta-analyses
Source: Oncotarget. 2016 May 4;7(23):34703–51. doi: 10.18632/oncotarget.9157 (PMC5085185; doi:10.18632/oncotarget.9157)
Supplement: Supplementary file 9 [file oncotarget-07-34703-s009.docx]

| Supplementary Table S25: Overlap of included studies among meta-analyses regarding RFA plus TACE | | | | | | | | | |  |
| --- | --- | --- | --- | --- | --- | --- | --- | --- | --- | --- |
| **First author** | **Dong** | | **Han** | | **Jiang** | | | **Kong** | |  |
| Journal (Year) | World J Gastroenterol (2014) | | J Intervent Radiol (China) (2013) | | Tumour Biol (2014) | | | Tumour Biol (2014) | |  |
| Publication type | Full text | | Full text | | Full text | | | Full text | |  |
| No. Included studies | 5 | | 8 | | 19 | | | 19 | |  |
| No. Included RCTs | 0 | | 0 | | 8 | | | 8 | |  |
| Included studies | Cheng BQ, et al. JAMA 2008;299:1669–1677. | | Bloomston M, et al. Am Surg 2002;68:827–831. | | Aikata H, et al. Hepatology 2006;44:A487. | | | Aikata H, et al. Hepatology 2006;44:A487. | |  |
|  | Morimoto M, et al. Cancer 2010;116:5452–5460. | | Cheng BQ, et al. JAMA 2008;299:1669–1677. | | Cheng BQ, et al. JAMA 2008;299:1669–1677. | | | Cheng BQ, et al. JAMA 2008;299:1669–1677. | |  |
|  | Peng ZW, et al. J Clin Oncol 2013;31:426–432. | | Dai XD, et al. Fan She Xue Shi Jian (Chinese) 2010;25:799–802. | | Hu YQ, et al. Dang Dai Yi Xue (Chinese) 2011;17:232–233. | | | Hu YQ, et al. Dang Dai Yi Xue (Chinese) 2011;17:232–233. | |  |
|  | Shibata T, et al. Radiology 2009;252:905–913. | | Hu YQ, et al. Dang Dai Yi Xue (Chinese) 2011;17:39–40. | | Kang CB, et al. Chin J Hepatobiliary Surg (Chinese) 2007;13:828–830. | | | Kang CB, et al. Chin J Hepatobiliary Surg (Chinese) 2007;13: 828–830. | |  |
|  | Zhao M, et al. Zhonghua Yi Xue Za Zhi 2011;91:1167–1172. | | Ji TL, et al. Xian Dai Zhong Liu Yi Xue (Chinese) 2004;12:540–544. | | Kim JH, et al. Ann Surg Oncol 2011;18:1624–1629. | | | Kim JH, et al. Ann Surg Oncol 2011;18: 1624–1629. | |  |
|  |  | | Lu LG, et al. Zhonghua Sheng Wu Yi Xue Gong Cheng Za Zhi (Chinese) 2007;13:294–297. | | Kim JW, et al. Eur J Radiol 2012;81:e189–193. | | | Kim JW, et al. Eur J Radiol 2012;81: e189–193. | |  |
|  |  | | Wu PH, et al. Zhonghua Fang She Xue Za Zhi (Chinese) 2002;37:901–904. | | Li ZR, et al. Nan Fang Yi Ke Da Xue Xue Bao (Chinese) 2007;27:1749–1751. | | | Li ZR, et al. Nan Fang Yi Ke Da Xue Xue Bao (Chinese) 2007;27:1749–1751. | |  |
|  |  | | Yang P, et al. Adv Ther 2008;25: 787–794. | | Liang MH. Zhong Guo Lao Nian Yi Xue Za Zhi (Chinese) 2011;31:2862–2864. | | | Liang MH. Zhong Guo Lao Nian Yi Xue Za Zhi (Chinese) 2011;31:2862–2864. | |  |
|  |  | |  | | Luo ZG, et al. Yunyang Yi Xue Yuan Xue Bao (Chinese) 2008;27:22–25. | | | Luo ZG, et al. Yunyang Yi Xue Yuan Xue Bao (Chinese) 2008;27: 22–25. | |  |
|  |  | |  | | Morimoto M, et al. Cancer 2010;116:5452–5460. | | | Morimoto M, et al. Cancer 2010;116: 5452–5460. | |  |
|  |  | |  | | Peng ZW, et al. Eur J Surg Oncol 2010;36:257–263. | | | Peng ZW, et al. Eur J Surg Oncol 2010;36:257–263. | |  |
|  |  | |  | | Shen L, et al. Zhonghua Chao Sheng Yin Xiang Xue Za Zhi (Chinese) 2004;13:577–580. | | | Shen L, et al. Zhonghua Chao Sheng Yin Xiang Xue Za Zhi (Chinese) 2004;13:577–580. | |  |
|  |  | |  | | Shen SQ, et al. Hepatogastroenterology 2005;52:1403–1407. | | | Shen SQ, et al. Hepatogastroenterology 2005;52:1403–1407. | |  |
|  |  | |  | | Shibata T, et al. Radiology 2009;252:905–913. | | | Shibata T, et al. Radiology 2009;252:905–913. | |  |
|  |  | |  | | Wang YB, et al. Ai Zheng (Chinese) 2005;24:827–833. | | | Wang YB, et al. Ai Zheng (Chinese) 2005;24:827–833. | |  |
|  |  | |  | | Xu L, et al. Zhonghua Wai Ke Za Zhi (Chinese) 2008;46: 1617–1620. | | | Xu L, et al. Zhonghua Wai Ke Za Zhi (Chinese) 2008;46:1617–1620. | |  |
|  |  | |  | | Yang P, et al. Adv Ther 2008;25:787–794. | | | Yang P, et al. Adv Ther 2008;25:787–794. | |  |
|  |  | |  | | Yang W, et al. Hepatol Res 2009;39:231–240. | | | Yang W, et al. Hepatol Res 2009;39:231–240. | |  |
|  |  | |  | | Zhang HC, et al. Zhong Guo Yi Shi Jin Xiu Za Zhi (Chinese) 2007;30:67–68. | | | Zhang HC, et al. Zhong Guo Yi Shi Jin Xiu Za Zhi (Chinese) 2007;30:67–68. | |  |
|  | | | | | | | | | |  |
| Overlap of included studies among meta-analyses regarding RFA plus TACE (continued 1) | | | | | | | | | |  |
| **First author** | **Liao** | | **Liu** | | | **Lu** | | **Ni** | |  |
| Journal (Year) | PLoS One (2013) | | Tumour Biol (2014) | | | Eur J Gastroenterol Hepatol (2013) | | World J Gastroenterol (2013) | |  |
| Publication type | Full text | | Full text | | | Full text | | Full text | |  |
| No. Included studies | 1 | | 7 | | | 7 | | 8 | |  |
| No. Included RCTs | 1 | | 7 | | | 7 | | 8 | |  |
| Included studies | Yang P, et al. Adv Ther 2008;25: 787–794. | | Aikata H, et al. Hepatology 2006;44:A487. | | | Aikata H, et al. Hepatology 2006;44:A487. | | Cheng BQ, et al. JAMA 2008;299:1669–1677. | |  |
|  |  | | Kang CB, et al. Zhonghua Gandan Waike Za Zhi (Chinese) 2007;13: 828–830. | | | Cheng BQ, et al. JAMA 2008;299:1669–1677. | | Kang CB, et al. Zhonghua Gandan Waike Za Zhi (Chinese) 2007;13:828–830. | |  |
|  |  | | Morimoto M, et al. Cancer 2010;116:5452–5460. | | | Kang CB, et al. Chin J Hepatobiliary Surg (Chinese) 2007;13: 828–830. | | Morimoto M, et al. Cancer 2010;116: 5452–5460. | |  |
|  |  | | Peng ZW, et al. Eur J Surg Oncol 2010;36:257–263. | | | Morimoto M, et al. Cancer 2010;116:5452–5460. | | Peng ZW, et al. Radiology 2012;262:689–700. | |  |
|  |  | | Peng ZW, et al. Radiology 2012;262:689–700. | | | Peng ZW, et al. Radiology 2012;262:689–700. | | Shen SQ, et al. Hepatogastroenterology 2005;52:1403–1407. | |  |
|  |  | | Shibata T, et al. Radiology 2009;252:905–913. | | | Shibata T, et al. Radiology 2009;252:905–913. | | Shibata T, et al. Radiology 2009;252:905–913. | |  |
|  |  | | Yang P, et al. Adv Ther 2008;25:787–794. | | | Yang P, et al. Adv Ther 2008;25:787–794. | | Yang P, et al. Adv Ther 2008;25:787–794. | |  |
|  |  | |  | | |  | | Zhang Z, et al. Zhonghua Wai Ke Za Zhi 2002;40:826–829. | |  |
| Overlap of included studies among meta-analyses regarding RFA plus TACE (continued 2) | | | | | | | | | | |
| **First author** | | **Ni** | | **Yan** | | | **Yan** | | **Zhao** | |
| Journal (Year) | | J Cancer Res Clin Oncol (2013) | | Dig Dis Sci (2012) | | | Dig Dis Sci (2013) duplicates | | J Intervent Radiol (China) (2013) | |
| Publication type | | Full text | | Full text | | | Full text | | Full text | |
| No. Included studies | | 6 | | 19 | | | 18 | | 21 | |
| No. Included RCTs | | 6 | | 8 | | | 8 | | 21 | |
| Included studies | | Morimoto M, et al. Cancer 2010;116:5452–5460. | | Aikata H, et al. Hepatology 2006;44:A487. | | | Aikata H, et al. Hepatology 2006;44:A487. | | Chen JM, et al. Shandong Yi Yao 2010;50:42–43. | |
|  | | Peng ZW, et al. Radiology 2012;262:689–700. | | Cheng BQ, et al. JAMA 2008;299:1669–1677. | | | Hu YQ, et al. Dang Dai Yi Xue (Chinese) 2011;17:232–233. | | Dai XD, et al. Fan She Xue Shi Jian (Chinese) 2010;25:799–802. | |
|  | | Shen SQ, et al. Hepatogastroenterology 2005;52:1403–1407. | | Hu YQ, et al. Dang Dai Yi Xue (Chinese) 2011;17:232–233. | | | Kang CB, et al. Chin J Hepatobiliary Surg (Chinese) 2007;13:828–830. | | Fang ZX, et al. Xian Dai Xiao Hua Ji Jie Ru Zhen Liao 2012;17: 1–4. | |
|  | | Shibata T, et al. Radiology 2009;252:905–913. | | Kang CB, et al. Chin J Hepatobiliary Surg (Chinese) 2007;13: 828–830. | | | Kim JH, et al. Ann Surg Oncol 2011;18: 1624–1629. | | Jin DG, et al. Zhong Guo Zhong Xi Yi Jie He Wai Ke Za Zhi 2009;15:141–143. | |
|  | | Zhang Z, et al. Zhonghua Wai Ke Za Zhi 2002;40:826–829. | | Kim JH, et al. Ann Surg Oncol 2011;18: 1624–1629. | | | Kim JW, et al. Eur J Radiol 2012;81: e189–193. | | Kang CB, et al. Chin J Hepatobiliary Surg (Chinese) 2007;13:828–830. | |
|  | | Zhao M, et al. Zhonghua Yi Xue Za Zhi 2011;91:1167–1172. | | Kim JW, et al. Eur J Radiol 2012;81: e189–193. | | | Li ZR, et al. Nan Fang Yi Ke Da Xue Xue Bao (Chinese) 2007;27:1749–1751. | | Hu YQ, et al. Dang Dai Yi Xue (Chinese) 2011;17:39–40. | |
|  | |  | | Li ZR, et al. Nan Fang Yi Ke Da Xue Xue Bao (Chinese) 2007;27: 1749–1751. | | | Liang MH. Zhong Guo Lao Nian Yi Xue Za Zhi (Chinese) 2011;31:2862–2864. | | Li QZ, et al. Qi Qi Ha Er Yi Xue Yuan Xue Bao 2012;33: 1717–1718. | |
|  | |  | | Liang MH. Zhong Guo Lao Nian Yi Xue Za Zhi (Chinese) 2011;31: 2862–2864. | | | Luo ZG, et al. Yunyang Yi Xue Yuan Xue Bao (Chinese) 2008;27: 22–25. | | Li ZR, et al. Nan Fang Yi Ke Da Xue Xue Bao (Chinese) 2007;27:1749–1751. | |
|  | |  | | Luo ZG, et al. Yunyang Yi Xue Yuan Xue Bao (Chinese) 2008;27: 22–25. | | | Morimoto M, et al. Cancer 2010;116: 5452–5460. | | Liang MH. Zhong Guo Lao Nian Yi Xue Za Zhi (Chinese) 2011;31:2862–2864. | |
|  | |  | | Morimoto M, et al. Cancer 2010;116: 5452–5460. | | | Peng ZW, et al. Eur J Surg Oncol 2010;36:257–263. | | Liu YM, et al. World J Gastroenterol 2006;12:5060–5063. | |
|  | |  | | Peng ZW, et al. Eur J Surg Oncol 2010;36:257–263. | | | Shen SQ, et al. Hepatogastroenterology 2005;52:1403–1407. | | Lu LG, et al. Zhonghua Sheng Wu Yi Xue Gong Cheng Za Zhi (Chinese) 2007;13:294–297. | |
|  | |  | | Shen SQ, et al. Hepatogastroenterology 2005;52:1403–1407. | | | Shibata T, et al. Radiology 2009;252:905–913. | | Luo ZG, et al. Yunyang Yi Xue Yuan Xue Bao (Chinese) 2008;27: 22–25. | |
|  | |  | | Shibata T, et al. Radiology 2009;252:905–913. | | | Wang YB, et al. Ai Zheng (Chinese) 2005;24:827–833. | | Shen L, et al. Zhonghua Chao Sheng Yin Xiang Xue Za Zhi (Chinese) 2004;13:577–580. | |
|  | |  | | Wang YB, et al. Ai Zheng (Chinese) 2005;24:827–833. | | | Xu L, et al. Zhonghua Wai Ke Za Zhi (Chinese) 2008;46:1617–1620. | | Song W, et al. Zhong Guo Xian Dai Pu Tong Wai Ki 2008;11: 203–207. | |
|  | |  | | Xu L, et al. Zhonghua Wai Ke Za Zhi (Chinese) 2008;46:1617–1620. | | | Yang P, et al. Adv Ther 2008;25:787–794. | | Tang SY, et al. Harbin University Xue Bao (Chinese) 2005;39:183–184, 187. | |
|  | |  | | Yang P, et al. Adv Ther 2008;25:787–794. | | | Yang W, et al. Hepatol Res 2009;39:231–240. | | Wang HB, et al. Zhong Guo Zong He Lin Chuang 2008;24: 941–944. | |
|  | |  | | Yang W, et al. Hepatol Res 2009;39:231–240. | | | Zhang HC, et al. Zhong Guo Yi Shi Jin Xiu Za Zhi (Chinese) 2007;30:67–68. | | Wang HT, et al. Henan Wai Ke Xue Za Zhi 2012;18:78–79. | |
|  | |  | | Zhang HC, et al. Zhong Guo Yi Shi Jin Xiu Za Zhi (Chinese) 2007;30:67–68. | | |  | | Yang P, et al. Adv Ther 2008;25:787–794. | |
|  | |  | |  | | |  | | Zhang CP, et al. Zhongguo Zhong Liu Lin Chuang (Chinese) 2004;31:1309–1310. | |
|  | |  | |  | | |  | | Zhang ZW. Dang Dai Yi Xue (Chinese) 2011;17:70–71. | |
|  | |  | |  | | |  | | Zhao M, et al. Zhonghua Yi Xue Za Zhi 2011;91:11 67–1172. | |
