# Supplementary material for: Management of hepatocellular carcinoma: an overview of major findings from meta-analyses
Source: Oncotarget. 2016 May 4;7(23):34703–51. doi: 10.18632/oncotarget.9157 (PMC5085185; doi:10.18632/oncotarget.9157)
Supplement: Supplementary file 10 [file oncotarget-07-34703-s010.docx]

| Supplementary Table S28: Overlap of included studies among meta-analyses regarding TACE or TAE versus no active treatment | | | | | | | | |
| --- | --- | --- | --- | --- | --- | --- | --- | --- |
| **First author** | **Camma** | | **Geschwind** | | **Leng** | | **Llovet** | |
| Journal (Year) | Radiology (2002) | | Am J Clin Oncol (2003) | | ANZ J Surg (2014) | | Hepatology (2003) | |
| Publication type | Full text | | Full text | | Full text | | Full text | |
| No. Included studies | 5 | | 4 | | 3 | | 7 | |
| No. Included RCTs | 5 | | 4 | | 2 | | 7 | |
| Included studies | Bruix J, et al. Hepatology 1998;27:1578–1583. | | Bruix J, et al. Hepatology 1998;27:1578–1583. | | Li Q, et al. World J Surg 2006; 30: 2004–2011. | | Bruix J, et al. Hepatology 1998;27:1578–1583. | |
|  | GETCH. N Engl J Med 1995;332:1256–1261. | | GETCH. N Engl J Med 1995;332: 1256–1261. | | Luo J, et al. Ann Surg Oncol 2011;18:413–420. | | GETCH. N Engl J Med 1995;332:1256–1261. | |
|  | Lin DY, et al. Gastroenterology 1988;94:453–456. | | Madden MV, et al. Gut 1993;34: 1598–600. | | Peng BG, et al. Am J Surg 2009;198:313–318. | | Lin DY, et al. Gastroenterology 1988;94:453–456. | |
|  | Llovet JM, et al. J Hepatol 2001;34:11A. | | Pelletier G, et al. J Hepatol 1998;29:129–134. | |  | | Llovet JM, et al. Lancet 2002;359(9319):1734–1739. | |
|  | Pelletier G, et al. J Hepatol 1998;29: 129–134. | |  | |  | | Lo CM, et al. Hepatology 2002;35:1164–1171. | |
|  |  | |  | |  | | Pelletier G, et al. J Hepatol 1998;29:129–134. | |
|  |  | |  | |  | | Pelletier G, et al. J Hepatol 1990;11:181–184. | |
| Overlap of included studies among meta-analyses regarding TACE or TAE versus no active treatment (continued) | | | | | | | |  |
| **First author** | | **Marelli** | | **Oliveri** | | **Xue** | |  |
| Journal (Year) | | Cardiovasc Intervent Radiol (2007) | | Cochrane Database Syst Rev (2011) | | BMC Gastroenterol (2013) | |  |
| Publication type | | Full text | | Full text | | Full text | |  |
| No. Included studies | | 9 | | 8 | | 8 | |  |
| No. Included RCTs | | 9 | | 8 | | 0 | |  |
| Included studies | | Bruix J, et al. Hepatology 1998;27:1578–1583. | | Akamatsu M, et al. Liver Int 2004;24(6):625–629. | | Chung GE, et al. Radiology 2011;258:627–634. | |  |
|  | | GETCH. N Engl J Med 1995;332:1256–1261. | | Bruix J, et al. Hepatology 1998;27:1578–1583. | | Kim JH, et al. Aliment Pharmacol Ther 2009;29: 1291–1298. | |  |
|  | | Lin DY, et al. Gastroenterology 1988;94:453–456. | | Doffoël M, et al. Euro J Cancer 2008;44(4):528–538. | | Kim KM, et al. J Gastroenterol Hepatol 2009;24:806–814. | |  |
|  | | Llovet JM, et al. Lancet 2002;359(9319):1734–1739. | | GETCH. N Engl J Med 1995;332:1256–1261. | | Lee HS, et al. Cancer 1997;11:2087–2094. | |  |
|  | | Lo CM, et al. Hepatology 2002;35:1164–1171. | | Llovet JM, et al. Lancet 2002;359(9319):1734–1739. | | Luo J, et al. Ann Surg Oncol 2011;18:413–420. | |  |
|  | | Madden MV, et al. Gut 1993;34:1598–600. | | Lo CM, et al. Hepatology 2002;35:1164–1171. | | Niu ZJ, et al. Med Oncol 2012;29:2992–2997. | |  |
|  | | Pelletier G, et al. J Hepatol 1998;29:129–134. | | Pelletier G, et al. J Hepatol 1998;29:129–134. | | Peng ZW, et al. Cancer 2012;118:4725–4736. | |  |
|  | | Pelletier G, et al. J Hepatol 1990;11:181–184. | | Pelletier G, et al. J Hepatol 1990;11:181–184. | | Zhou Q, et al. Asian Pac J Cancer Prev 2011;12: 2847–2850. | |  |
|  | | Yoshikawa M, et al. Cancer Chemother Pharmacol 1994;33:S149–S152. | |  | |  | |  |
| Abbreviations: GETCH, Groupe d’Etude et de Traitment du Carcinome Hepatocellulaire. | | | | | | | |  |
