# Supplementary material for: Management of hepatocellular carcinoma: an overview of major findings from meta-analyses
Source: Oncotarget. 2016 May 4;7(23):34703–51. doi: 10.18632/oncotarget.9157 (PMC5085185; doi:10.18632/oncotarget.9157)
Supplement: Supplementary file 11 [file oncotarget-07-34703-s011.docx]

| Supplementary Table S34: Overlap of included studies among meta-analyses regarding TACE + traditional Chinese medicine versus TACE alone | | | | | | |  |
| --- | --- | --- | --- | --- | --- | --- | --- |
| **First author** | **Cheung** | | | **Cho** | **Meng** | |  |
| Journal (Year) | Evid Based Complement Alternat Med (2013) | | | Expert Opin Investig Drugs (2009) | J Altern Complement Med (2008) | |  |
| Publication type | Full text | | | Full text | Full text | |  |
| No. Included studies | 67 | | | 30 | 37 | |  |
| No. Included RCTs | 67 | | | 30 | Not reported | |  |
| Included studies | Ayi XG, et al. Xian Dai Zhong Liu Yi Xue 2011;19(1):111–114. | | | Feng ZQ, et al. Chin Arch Tradit Chin Med 2007;25:2404–2407. | Cao GW, et al. Shandong Yi Yao 2005;45:13–14. | |  |
|  | Bao LQ. Hubei Zhong Yi Xue Yuan Xue Bao 2007;9(4):54–55. | | | Hu HL, et al. Henan J Oncol 2001;14:412–413. | Cao MR, et al. Zhongguo Zhong Xi Yi Jie He Za Zhi 2003;23: 713–714. | |  |
|  | Cao GW, et al. Shandong Yi Yao 2005;45:13–14. | | | Li CQ, et al. China J Cancer Prev Treat 2003;10: 1209–1210. | Chen C, et al. Zhong Xi Yi Jie He Gan Bing Za Zhi 2001;11: 183–184. | |  |
|  | Chen SC, et al. Fujian Zhong Yi Xue Yuan Xue Bao 2007;17(5):14–16. | | | Liang QL, et al. Chin Arch Tradit Chin Med 2006;24: 186–188. | Feng J. Guangxi Zhong Yi Xue Yuan Xue Bao 2002;5:51–52. | |  |
|  | Dan ZS, et al. Zhongguo Zhong Xi Yi Jie He Za Zhi 2007;27(1):83–84. | | | Lin J, et al. Chin J Pract Chin Mod Med 2006;19:692–693. | Guo TS, et al. Shiyong Yi Xue Za Zhi 2005;21:1846–1847. | |  |
|  | Deng L, et al. Xin Zhong Yi 2009;41(4):28–29. | | | Liu JL, et al. Tradit Chin Med Res 2005;18:34–36. | Li QM, et al. Sichuan Zhong Liu Fang Zhi 2003;16:160–161. | |  |
|  | Dong HT, et al. Zhongguo Zhong Yi Yao Xin Xi Za Zhi 2007;14(12):62–63. | | | Meng ZQ, et al. J Chin Integr Med 2003;1:187–188. | Li RJ. Zhong Yuan Yi Kan 2005;14:69. | |  |
|  | Dong HT, et al. Zhongguo Zhong Liu Lin Chuang 2008;35(7):378–380. | | | Ou YY, et al. Guangzhou Med J 2006;37:69–70. | Li WH, et al. World Clin Drugs 2006;27:304–306. | |  |
|  | Feng J. Guangxi Zhong Yi Xue Yuan Xue Bao 2002;5:51–52. | | | Ren HP, et al. Chin J Integr Tradit West Med 2004;24:838–840. | Lin LZ, et al. Zhongguo Zhong Xi Yi Jie He Za Zhi 2005;25:8–11. | |  |
|  | Guo TS, et al. Shiyong Yi Xue Za Zhi 2005;21:1846–1847. | | | Shao ZX, et al. Zhongguo Zhong Xi Yi Jie He Za Zhi 2001;21:168–170. | Liu XL, et al. Ningxia Yi Xue Yuan Xue Bao 2002;24:105–106. | |  |
|  | Han WQ. Zhongguo Zhong Yi Yao Xian Dai Yuan Cheng Jiao Yu 2009;7(2):103. | | | Sun XP, et al. Chin Imaging J Integr Tradit West Med 2007;5:84–86. | Long JA, et al. Zhong Xi Yi Jie He Gan Bing Za Zhi 2001;11:275–277. | |  |
|  | Hou EC, et al. Zhongguo Zhong Xi Yi Jie He Za Zhi 2009;29(3):225–227. | | | Wang CJ, et al. Chin J Integr Tradit West Med Digestion 2002;10:211–214. | Tan XY, et al. Zhongliu Fang Zhi Yan Jiu 2005;35:313–314. | |  |
|  | Huang JY, et al. Shi Yong Zhong Liu Za Zhi 2002;17(5):352–353. | | | Wang LJ, et al. Mod Oncol 2007;15:1812–1813. | Tian HQ, et al. Henan Zhong Yi Yao Xue Kan 2001;16:47–48. | |  |
|  | Huang JY. Shi Jie Zhong Yi Yao 2008;3(5):271. | | | Wang T. J Gansu Coll Tradi Chin Med 2004;21:19–20. | Tian XL. Zhong Yuan Yi Kan 2006;33:32–34. | |  |
|  | Jia YS, et al. Zhong Guo Zhong Yao Za Zhi 2003;28(7): 683–684. | | | Wen HY, et al. Shaanxi Zhong Yi 2006;27:26–28. | Wang HZ. ZhongGuo Zhong Xi Yi Jie He Za Zhi 1998;18: 411–413. | |  |
|  | Li DJ, et al. Chinese-German Journal of Clinical Oncology 2009;8(2):65–68. | | | Wu XD, et al. Chin J Integr Tradit West Med Liver Dis 2002;12:43–44. | Wang QP, et al. Linchuang Huicui 2006;21:580–581. | |  |
|  | LI M. Zhongguo Yi Yao Dao Bao 2007;31(4):40–41. | | | Xu K, et al. J Tradit Chin Med 2000;20:185–186. | Wang RP, et al.Anhui Zhong Yi Xue Yuan Xue Bao 2002;21:9–11. | |  |
|  | Li RT, et al. Hunan Zhong Yi Za Zhi 2008;24(2):60–61. | | | Xu YQ, et al. J New Chin Med 2007;39:86–87. | Wang ZX, et al. Henan Zhong Yi 2001;21:48–49. | |  |
|  | Liang JX, et al. Guang Zhou Yi Yao 2005;36(3):45–47. | | | Xu ZG, et al. Chin J Surg Integr Tradit West Med 2005;11:480–481. | Wen HY, et al. Shaanxi Zhong Yi 2006;21:26–28. | |  |
|  | Liang M, et al. Zhongguo Yao Fang 2008;19(33):2619–2621. | | | Xue YB, et al. Mod J Integr Tradit Chin West Med 2002;11:2218–2219. | Wu JX. Zhejiang Zhong Xi Yi Jie He Za Zhi 1999;9:100–101. | |  |
|  | Liang TJ, et al. Journal of Traditional Chinese Medicine 2005;16(6):146. | | | Yi C, et al. Pharmacol Clin Clin Mater Med 1998;14: 39–40. | Wu WG, et al. Zhong Xi Yi Jie He Gan Bing Za Zhi 2001;11:50–51. | |  |
|  | Ling ZQ. Ji Lin Yi Xue 2010;31(15):2284. | | | Yu Y. Mod Diagn Treat 2002;13:1–3. | Wu XD, et al. Beijing Zhong Yi Yao Da Xue Xue Bao 2003;26:67–68. | |  |
|  | Liu HQ, et al. Hannan Yi Xue 2007;18(8):19–20. | | | Zhang H, et al. Chin J Med Offic 2007;35:857–859. | Xiang DB, et al.Xiandai Zhongliu Yi Xue 2006;14:861–862. | |  |
|  | Lu AP, et al. Zhongguo She Qu Yi Shi 2009;20(11):167. | | | Zhang R, et al. Shaanxi J Tradit Chin Med 2007;28:1117–11118. | Xu ZW, et al. Zhejiang Zhong Xi Yi Jie He Za Zhi 2000;10: 711–713. | |  |
|  | Lu RM, et al. Zhong Yi Yao Xin Xi 2010;27(6):33–35. | | | Zhang YH, et al. China Pharm 2007;18:611–612. | Yang JM. Yi Yao Lun Tan Za Zhi 2006;27:26–27. | |  |
|  | Lu YX, et al. Zhong Yi Yao Xin Xi 2010;27(6):33–35. | | | Zhang YM, et al. Shaanxi Zhong Yi Xue Yuan Xue Bao 2005;28:31–32. | Yu QT, et al. Guangxii Zhong Yi Xue Yuan Xue Bao 2004;7:35–37. | |  |
|  | Meng SX. Zhongguo Zhong Yi Ji Zheng 2008;17(11): 1523–1524. | | | Zhao ZH, et al. J Emerg Tradit Chin Med 2005;14:1053–1054. | Zhang CQ, et al. Beijing Yi Xue 2005;27:357–359. | |  |
|  | Shi GJ, et al. Henan Zhong Yi Xue Yuan Xue Bao 2005;20(120):37–38. | | | Zheng WD, et al. Shanghai J Tradit Chin Med 2002;12:7–8. | Zhang L, et al. Chin J Primary Med 2005;12:1010–1011. | |  |
|  | Qiao LJ, et al. Zhong Yi Xue Bao 2010;25(148):393–395. | | | Zhou JF, et al. Her Med 2005;24:135–136. | Zhang SY, et al. Zhong Yi Yao Xin Xi 1996;4:29–31. | |  |
|  | Sun ZJ, et al. Zhong Liu Fang Zhi Yan Jiu. 2002;29:67–68. | | | Zhou XY, et al. Jiangsu J Tradit Chin Med 2002;23: 15–17. | Zhang YF, et al. ZhongGuo Zhong Xi Yi Jie He Wai Ke Za Zhi 2000;6:179–180. | |  |
|  | Tang XY, et al. Xin Zhong Yi 2010;42(11):78–80. | | |  | Zhang YM, et al. Shaanxi Zhong Yi Xue Yuan Xue Bao 2005;28:31–32. | |  |
|  | Tian HQ, et al. Henan Zhong Yi Yao Xue Kan 2001;16:47–48. | | |  | Zhao HR, et al. Xinjiang Zhong Yi Yao 2004;22:27–28. | |  |
|  | Tian XL. Zhong Yuan Yi Kan 2006;33:32–34. | | |  | Zhao XW, et al. Zhong Yi Yao Xue Bao 2005;33:28–29. | |  |
|  | Wang CJ, et al. Chin J Integr Tradit West Med Digestion 2002;10:211–214. | | |  | Zhou BG, et al. Xin Zhong Yi 2002;34:37–38. | |  |
|  | Wang HM, et al. Modern Journal of Integrated Traditional Chinese and Western Medicine 2009;18(12):1334–1335. | | |  | Zhou BG, et al. Shaanxi Zhong Liu Yi Xue 1999;7:159–161. | |  |
|  | Wang RP, et al. Anhui Zhong Yi Xue Yuan Xue Bao 2002;21:9–11. | | |  | Zhou JS, et al. Zhong Guo Ji Ceng Yi Yao 2006;13: 571–572. | |  |
|  | Wang RP, et al. Nanjing Zhong Yi Yao Da Xue Xue Bao 2008;24(2):130–132. | | |  | Zhu XF. Zhongliu Jichu Yu Linchuang 2006;19:132–134. | |  |
|  | Wang YG, et al. Lin Chuang Hui Cui 2007;22(19): 1429–1430. | | |  |  | |  |
|  | Wang ZX, et al. Zhongguo Shi Yong Yi Yao 2008;30(3):37–38. | | |  |  | |  |
|  | Weng H, et al. Sichuan Zhong Yi 2008;26(4):59–60. | | |  |  | |  |
|  | Wu HM, et al. Liaoning Journal of Traditional Chinese Medicine 2000;27(3):127–128. | | |  |  | |  |
|  | Wu JX. Zhejiang Zhong Xi Yi Jie He Za Zhi 1999;9(2): 100–101. | | |  |  | |  |
|  | Wu XD, et al. Beijing Zhong Yi Yao Da Xue Xue Bao 2003;26(1):67–68. | | |  |  | |  |
|  | Xu JX, et al. Nei Meng Gu Yi Xue Za Zhi 2006;38(2):180–181. | | |  |  | |  |
|  | Xu YQ, et al. Shi Zhen Guo Yi Guo Yao 2007;18(9): 2238–2239. | | |  |  | |  |
|  | Xu YQ, et al. J New Chin Med 2007;39:86–87. | | |  |  | |  |
|  | Xue YB, et al. Mod J Integr Tradit Chin West Med 2002;11:2218–2219. | | |  |  | |  |
|  | Yang LJ. Hei Long Jiang Zhong Yi Yao 2010;1:7–8. | | |  |  | |  |
|  | Yang JM. Yi Yao Lun Tan Za Zhi 2006;27:26–27. | | |  |  | |  |
|  | Yang XL. Zhong Yi Yan Jiu 2006;19(7):30–31. | | |  |  | |  |
|  | Yi JZ, et al. Zhong Liu 2008;28(11):997–1000. | | |  |  | |  |
|  | Yu ML, et al. Zhongguo Yi Yao Zhi Nan 2010;8(7):123–125. | | |  |  | |  |
|  | Yuan CJ, et al. Hubei Zhong Yi Xue Yuan Xue Bao 2010;12(5):50–51. | | |  |  | |  |
|  | Yuan HX, et al. Lin Chuang Zhong Liu Xue Za Zhi 2005;10:64–66. | | |  |  | |  |
|  | Zhai RQ, et al. Shanghai Zhong Yi Yao Za Zhi 2010;44(11): 48–50. | | |  |  | |  |
|  | Zhang CQ, et al. Beijing Yi Xue 2005;27:357–359. | | |  |  | |  |
|  | Zhang H, et al. Hunan Zhong Yi Yao Da Xue Xue Bao 2007;27(6):55–57. | | |  |  | |  |
|  | Zhang HT, et al. Zhong Xi Yi Jie He Gan Bing Za Zhi 2008;18(3):178–180. | | |  |  | |  |
|  | Zhang JM, et al. Shi Yong Zhong Xi Yi Jie He Lin Chuang 2008;8(2):20–21. | | |  |  | |  |
|  | Zhang Q, et al. Zhong Yi Za Zhi 2007;48(3):235–236. | | |  |  | |  |
|  | Zhang XY. Dang Dai Yi Xue 2011;17(6):161–164. | | |  |  | |  |
|  | Zhang YF, et al. ZhongGuo Zhong Xi Yi Jie He Wai Ke Za Zhi 2000;6:179–180. | | |  |  | |  |
|  | Zhao XW, et al. Zhong Yi Yao Xue Bao 2005;33:28–29. | | |  |  | |  |
|  | Zhao ZH, et al. Zhongguo Zhong Yi Yao Xin Xi Za Zhi 2006;13(1):63. | | |  |  | |  |
|  | Zhou XY, et al. Jiangsu J Tradit Chin Med 2002;23:15–17. | | |  |  | |  |
|  | Zhou XZ, et al. Hunan Zhong Yi Yao Da Xue Xue Bao 2010;30(11):55–57. | | |  |  | |  |
|  | Zou JF, et al. Zhongguo Zhong Xi Yi Jie He Xiao Hua Za Zhi 2004;12(1):52–53. | | |  |  | |  |
| Overlap of included studies among meta-analyses regarding TACE + traditional chinese medicine versus TACE alone (continued) | | | | | | | |
| **First author** | | **Meng** | **Sun** | | | **Wu** | |
| Journal (Year) | | Explore (NY) (2011) | Afr J Tradit Complement Altern Med (2012) | | | J Cancer Res Ther (2014) | |
| Publication type | | Full text | Full text | | | Full text | |
| No. Included studies | | 12 | 10 | | | 9 | |
| No. Included RCTs | | 11 | 0 | | | 0 | |
| Included studies | | Cao GW, et al. Shandong Yi Yao 2005;45:13–14. | Cao J, et al. Shandong Medicine 2009;49(4): 74–76. | | | Mod Dig Interv 2013;18:32–3. | |
|  | | Li J, et al. Yi Xue Li Lun Yu Shi Jian 1998;11:341–343. | Chen YL, et al. Journal of Tianjin University of Traditional Chinese Medicine 2006;25(3):166–167. | | | Dong HT, et al. Chin J Inf Tradit Chin Med 2008;15:66–7. | |
|  | | Song HY, et al. Zhong Xi Yi Jie He Gan Bing Za Zhi 2000;10(4):5–6. | Chen GH, et al. Chin J Int egr T rad West Med Dig 2007;15(4):239–241. | | | Li Q, et al. Acta Univ Tradit Med Sinensis Pharmacol Shanghai 2008;22:32–4. | |
|  | | Sun ZJ, et al. Zhong Liu Fang Zhi Yan Jiu. 2002;29:67–68. | Deng L, et al. Journal of New Chinese Medicine 2009;41(4):28–29. | | | Liang Y, et al. Mod J Integr Tradit Chin West Med 2008;17:1628–30. | |
|  | | Wang HZ. ZhongGuo Zhong Xi Yi Jie He Za Zhi 1998;18:411–413. | Guan CN, et al. China Journal of Chinese Materia Medica 2006;31(6):510–512. | | | Liu XH, et al. Chin J Mod Drug Appl 2009;134–5. | |
|  | | Wang RP, et al.Anhui Zhong Yi Xue Yuan Xue Bao 2002;21:9–11. | Huang RW, et al. Hebei Medicine 20006;12(5):443–445. | | | Liu YQ, et al. Chin Rural Health Serv Adm 2010;30:402–4. | |
|  | | Wen HY, et al. Shaanxi Zhong Yi 2006;21:26–28. | Wang HM, et al. Modern Journal of Integrated Traditional Chinese and Western Medicine 2009;18(12):1334–1335. | | | Xue S, et al. Jiangsu J Tradit Chin Med 2010;42:22–4. | |
|  | | Xiang DB, et al.Xiandai Zhongliu Yi Xue 2006;14:861–862. | Xu P, et al. Evaluation and analysis of drug–use in hospitals of China 2010;10(5):457–458. | | | Yang YG, et al. Chin Rural Health Serv Adm 2006;22:20. | |
|  | | Yuan HX, et al. Lin Chuang Zhong Liu Xue Za Zhi 2005;10:64–66. | Yang SM, et al. Medicine Industry Information 2006;3(17):209–210. | | | Zhou JS, et al. Zhong Guo Ji Ceng Yi Yao 2006;13: 571–572. | |
|  | | Zhang H, et al. Zhongguo Zhong Yi Yao Xin Xi Za Zhi. 2004;11:439–440. | Yu ML, et al. Guide of China Medicine 2009;8(7):123–125. | | |  | |
|  | | Zheng Q, et al. Jiang Su Yi Yao. 2005;31:469–470. |  | | |  | |
|  | | Zhang SY, et al. Zhong Yi Yao Xin Xi 1996;4:29–31. |  | | |  | |
