# Supplementary material for: Management of hepatocellular carcinoma: an overview of major findings from meta-analyses
Source: Oncotarget. 2016 May 4;7(23):34703–51. doi: 10.18632/oncotarget.9157 (PMC5085185; doi:10.18632/oncotarget.9157)
Supplement: Supplementary file 12 [file oncotarget-07-34703-s012.docx]

| Supplementary Table S36: Overlap of included studies among meta-analyses regarding sorafenib | | | | | | | |  |
| --- | --- | --- | --- | --- | --- | --- | --- | --- |
| **First author** | | **Cinco** | **Duffy** | | **Shen** | | **Wang** |  |
| Journal (Year) | | Hepatol Int (2011) | Hepatology (2013) | | J Clin Gastroenterol (2013) | | Asian Pac J Cancer Prev (2013) |  |
| Publication type | | Full text | Full text | | Full text | | Full text |  |
| No. Included studies | | 2 | 4 | | 5 | | 4 |  |
| No. Included RCTs | | 2 | 4 | | 5 | | 4 |  |
| Included studies | | Not reported | Abou-Alfa GK, et al. JAMA 2010;304: 2154–2160. | | Abou-Alfa GK, et al. JAMA 2010;304:2154–2160. | | Abou-Alfa GK, et al. JAMA 2010;304: 2154–2160. |  |
|  | |  | Cheng AL, et al. Lancet Oncol 2009;10:25–34. | | Cheng AL, et al. Lancet Oncol 2009;10:25–34. | | Cheng AL, et al. Lancet Oncol 2009;10:25–34. |  |
|  | |  | Kudo M, et al. Eur J Cancer 2010;47:2117–2127. | | Kudo M, et al. Eur J Cancer 2010;47:2117–2127. | | Kudo M, et al. Eur J Cancer 2010;47: 2117–2127. |  |
|  | |  | Llovet JM, et al. N Engl J Med 2008;359:378–390. | | Llovet JM, et al. N Engl J Med 2008;359:378–390. | | Llovet JM, et al. N Engl J Med 2008;359: 378–390. |  |
|  | |  |  | | Sansonno D, et al. Oncologist 2012;17:359–366. | |  |  |
| Overlap of included studies among meta-analyses regarding sorafenib (continued) | | | | | | | | |
| **First author** | **Zhang** | | | **Zhang** | | **Zou** | | |
| Journal (Year) | Anticancer Drugs (2010) | | | Hepatobiliary Pancreat Dis Int (2012) | | Acta Academiae Medicinae Sinicae (2011) | | |
| Publication type | Full text | | | Full text | | Full text | | |
| No. Included studies | 3 | | | 3 | | 2 | | |
| No. Included RCTs | 3 | | | 3 | | 2 | | |
| Included studies | Abou-Alfa GK, et al. JAMA 2010;304:2154–2160. | | | Abou-Alfa GK, et al. JAMA 2010;304:2154–2160. | | Cheng AL, et al. Lancet Oncol 2009;10:25–34. | | |
|  | Cheng AL, et al. Lancet Oncol 2009;10:25–34. | | | Cheng AL, et al. Lancet Oncol 2009;10:25–34. | | Llovet JM, et al. N Engl J Med 2008;359:378–390. | | |
|  | Llovet JM, et al. N Engl J Med 2008;359:378–390. | | | Llovet JM, et al. N Engl J Med 2008;359:378–390. | |  | | |
