# Supplementary material for: Management of hepatocellular carcinoma: an overview of major findings from meta-analyses
Source: Oncotarget. 2016 May 4;7(23):34703–51. doi: 10.18632/oncotarget.9157 (PMC5085185; doi:10.18632/oncotarget.9157)
Supplement: Supplementary file 13 [file oncotarget-07-34703-s013.docx]

| Supplementary Table S37: Overlap of included studies among meta-analyses regarding antiviral therapy | | | | | | | | | | | | | | | | | | | |
| --- | --- | --- | --- | --- | --- | --- | --- | --- | --- | --- | --- | --- | --- | --- | --- | --- | --- | --- | --- |
| **First author** | | | **Breitenstein** | | **Huang** | | | | | **Jiang** | | | | | | **Lan** | | | |
| Journal (Year) | | | Br J Surg (2009) | | J Viral Hepat (2013) | | | | | World J Surg Oncol (2013) | | | | | | J Gastroenterol Hepatol Res (Hong Kong) (2013) | | | |
| Publication type | | | Full text | | Full text | | | | | Full text | | | | | | Full text | | | |
| No. Included studies | | | 7 | | 23 | | | | | 10 | | | | | | 10 | | | |
| No. Included RCTs | | | 7 | | 10 | | | | | 8 | | | | | | 0 | | | |
| Included studies | | | Ikeda K, et al. Hepatology 2000;32:228–232. | | Akamatsu M, et al. Liver Int 2006;26: 536–542. | | | | | Ishikawa T, et al. Hepatogastroenterology 2012;59:529–532. | | | | | | Chan AC, et al. Arch Surg 2011;146(6): 675–681. | | | |
|  | | | Kubo S, et al. Br J Surg 2002;89:418–422. | | Hagihara H, et al. Int J Clin Oncol 2011;16:210–220. | | | | | Kubo S, et al. Br J Surg 2002;89:418–422. | | | | | | Chuma M, et al. J Gastroenterol 2009;44:991–999. | | | |
|  | | | Lin SM, et al. Cancer 2004;100:376–382. | | Hung CH, et al. J Gastroenterol Hepatol 2005;20:1553–1559. | | | | | Li M, et al. J Gastroenterol Hepatol 2009;24(8):1437–1444. | | | | | | Hung IF, et al. Am J Gastroenterol 2008;103:1663–1673. | | | |
|  | | | Lo CM, et al. Ann Surg 2007;245: 831–842. | | Ikeda K, et al. Hepatol Res 2010;40: 1168–1175. | | | | | Li N, et al. Ann Surg Oncol 2010;17(1): 179–185. | | | | | | Koda M, et al. Intern Med 2009;48:11–17. | | | |
|  | | | Mazzaferro V, et al. Hepatology 2006;44:1543–1554. | | Ishikawa T, et al. Hepatogastroenterology 2012;59:529–532. | | | | | Lin SM, et al. Cancer 2004;100:376–382. | | | | | | Kubo S, et al. Hepatol Res 2007;37:94–100. | | | |
|  | | | Shiratori Y, et al. Ann Intern Med 2003;138:299–306. | | Jeong S, et al. World J Gastroenterol 2007;13:5188–5195. | | | | | Lo CM, et al. Ann Surg 2007;245:831–842. | | | | | | Kuzuya T, et al. J Gastroenterol Hepatol 2007; 22:1929–1935. | | | |
|  | | | Sun HC, et al. J Cancer Res Clin Oncol 2006;132:458–465. | | Jeong SC, et al. World J Gastroenterol 2007;13:5343–5350. | | | | | Mazzaferro V, et al. Hepatology 2006;44:1543–1554. | | | | | | Li N, et al. Ann Surg Oncol 2010;17(1): 179–185. | | | |
|  | | |  | | Katagiri S, et al. Nihon Shokakibyo Gakkai Zasshi 2008;105: 795–801. | | | | | Shiratori Y, et al. Ann Intern Med 2003;138:299–306. | | | | | | Piao CY, et al. Acta Med Okayama 2005;59:217–224. | | | |
|  | | |  | | Kubo S, et al. Br J Surg 2002;89:418–422. | | | | | Sun HC, et al. J Cancer Res Clin Oncol 2006;132:458–465. | | | | | | Shuqun C, et al. Hepatogastroenterology 2006;53:249–252. | | | |
|  | | |  | | Kudo M, et al. Oncology 2007;72(Suppl. 1): 132–138. | | | | | Piao CY, et al. Acta Med Okayama 2005;59:217–224. | | | | | | Yoshida H, et al. Hepatol Int 2008;2: 89–94. | | | |
|  | | |  | | Lin SM, et al. Cancer 2004;100:376–382. | | | | |  | | | | | |  | | | |
|  | | |  | | Lo CM, et al. Ann Surg 2007;245:831–842. | | | | |  | | | | | |  | | | |
|  | | |  | | Mazzaferro V, et al. Hepatology 2006;44:1543–1554. | | | | |  | | | | | |  | | | |
|  | | |  | | Miyaguchi S, et al. Hepatogastroenterology 2002;49:724–729. | | | | |  | | | | | |  | | | |
|  | | |  | | Nishiguchi S, et al. Intervirology 2005;48(1):71–75. | | | | |  | | | | | |  | | | |
|  | | |  | | Qu LS, et al. J Surg Oncol 2010;102: 796–801. | | | | |  | | | | | |  | | | |
|  | | |  | | Sakaguchi Y, et al. Intervirology 2005;48:64–70. | | | | |  | | | | | |  | | | |
|  | | |  | | Shiratori Y, et al. Ann Intern Med 2003;138:299–306. | | | | |  | | | | | |  | | | |
|  | | |  | | Someya T, et al. J Gastroenterol 2006;41:1206–1213. | | | | |  | | | | | |  | | | |
|  | | |  | | Sun HC, et al. J Cancer Res Clin Oncol 2006;132:458–465. | | | | |  | | | | | |  | | | |
|  | | |  | | Suou T, et al. Hepatol Res 2001;20:301–311. | | | | |  | | | | | |  | | | |
|  | | |  | | Tanimoto Y, et al. Ann Surg Oncol 2012;19:418–425. | | | | |  | | | | | |  | | | |
|  | | |  | | Zhang CH, et al. Int J Cancer 2011;129: 1254–1264. | | | | |  | | | | | |  | | | |
| Overlap of included studies among meta-analyses regarding antiviral therapy (continued 1) | | | | | | | | | | | | | | | | | | |  |
| **First author** | **Li** | | | | | | | **Miao** | | | | | **Miyake** | | | | | **Moriguchi** |  |
| Journal (Year) | Chinese J Cancer Prevention and Treatment (2013) | | | | | | | World J Gastroenterol (2010) | | | | | J Viral Hepat (2010) | | | | | Hepatology (2006) |  |
| Publication type | Full text | | | | | | | Full text | | | | | Full text | | | | | Abstract |  |
| No. Included studies | 8 | | | | | | | 13 | | | | | 10 | | | | | 4 |  |
| No. Included RCTs | 8 | | | | | | | 5 | | | | | 3 | | | | | 4 |  |
| Included studies | Chen LT, et al. Ann Surg 2012;255:8–17. | | | | | | | Akamatsu M, et al. Liver Int 2006;26:536–542. | | | | | Jeong S, et al. World J Gastroenterol 2007;13:5188–5195. | | | | | NA |  |
|  | Ikeda K, et al. Hepatology 2000;32:228–232. | | | | | | | Hung CH, et al. J Gastroenterol Hepatol 2005;20:1553–1559. | | | | | Jeong SC, et al. World J Gastroenterol 2007;13:5343–5350. | | | | |  |  |
|  | Kubo S, et al. Br J Surg 2002;89:418–422. | | | | | | | Ikeda K, et al. Hepatology 2000;32:228–232. | | | | | Kubo S, et al. Ann Intern Med 2001;134:963–967. | | | | |  |  |
|  | Lin SM, et al. Cancer 2004;100:376–382. | | | | | | | Jeong S, et al. World J Gastroenterol 2007;13:5188–5195. | | | | | Kubo S, et al. Br J Surg 2002;89:418–422. | | | | |  |  |
|  | Lo CM, et al. Ann Surg 2007;245:831–842. | | | | | | | Jeong SC, et al. World J Gastroenterol 2007;13:5343–5350. | | | | | Kudo M, et al. Oncology 2007;72(Suppl. 1): 132–138. | | | | |  |  |
|  | Mazzaferro V, et al. Hepatology 2006;44:1543–1554. | | | | | | | Kuzuya T, et al. J Gastroenterol Hepatol 2007; 22:1929–1935. | | | | | Nishiguchi S, et al. Intervirology 2005;48(1):71–75. | | | | |  |  |
|  | Shiratori Y, et al. Ann Intern Med 2003;138:299–306. | | | | | | | Lin SM, et al. Cancer 2004;100:376–382. | | | | | Sakaguchi Y, et al. Intervirology 2005;48:64–70. | | | | |  |  |
|  | Sun HC, et al. J Cancer Res Clin Oncol 2006;132:458–465. | | | | | | | Lo CM, et al. Ann Surg 2007;245:831–842. | | | | | Shiratori Y, et al. Ann Intern Med 2003;138:299–306. | | | | |  |  |
|  |  | | | | | | | Mazzaferro V, et al. Hepatology 2006;44:1543–1554. | | | | | Suou T, et al. Hepatol Res 2001;20:301–311. | | | | |  |  |
|  |  | | | | | | | Omata M, et al. Clin Gastroenterol Hepatol 2005;3:S141–S143. | | | | | Uenishi T, et al. J Surg Oncol 2008;98: 358–362. | | | | |  |  |
|  |  | | | | | | | Piao CY, et al. Acta Med Okayama 2005;59: 217–224. | | | | |  | | | | |  |  |
|  |  | | | | | | | Sakaguchi Y, et al. Intervirology 2005;48:64–70. | | | | |  | | | | |  |  |
|  |  | | | | | | | Someya T, et al. J Gastroenterol 2006;41:1206–1213. | | | | |  | | | | |  |  |
| Overlap of included studies among meta-analyses regarding antiviral therapy (continued 2) | | | | | | | | | | | | | | | | | | |  |
| **First author** | | **Shen** | | | | **Singal** | | | | | | **Sun** | | | **Wang** | | | |  |
| Journal (Year) | | J Hepatol (2010) | | | | Aliment Pharmacol Ther (2010) | | | | | | PLoS One (2014) | | | Can J Gastroenterol (2013) | | | |  |
| Publication type | | Full text | | | | Full text | | | | | | Full text | | | Full text | | | |  |
| No. Included studies | | 13 | | | | 10 | | | | | | 13 | | | 9 | | | |  |
| No. Included RCTs | | 9 | | | | 5 | | | | | | 1 | | | 9 | | | |  |
| Included studies | | Chen LT, et al. Ann Surg 2012;255:8–17. | | | | Hung CH, et al. J Gastroenterol Hepatol 2005;20:1553–1559. | | | | | | Chan ACY, et al. Arch Surg 2011;146: 675–681. | | | Ikeda K, et al. Hepatology 2000;32:228–232. | | | |  |
|  | | Jeong S, et al. World J Gastroenterol 2007;13:5188–5195. | | | | Ikeda K, et al. Hepatology 2000;32:228–232. | | | | | | Chuma M, et al. J Gastroenterol 2009;44:991–999. | | | Kubo S, et al. Ann Intern Med 2001;134:963–967. | | | |  |
|  | | Jeong SC, et al. World J Gastroenterol 2007;13:5343–5350. | | | | Jeong SC, et al. World J Gastroenterol 2007;13:5343–5350. | | | | | | Hann HW, et al. Int J Cancer 2011;128: 740–743. | | | Kubo S, et al. Br J Surg 2002;89: 418–422. | | | |  |
|  | | Kubo S, et al. Ann Intern Med 2001;134:963–967. | | | | Kubo S, et al. Br J Surg 2002;89:418–422. | | | | | | Ke Y, et al. Cancer Biol Med 2013;10:158–164. | | | Lin SM, et al. Cancer 2004;100:376–382. | | | |  |
|  | | Kubo S, et al. Br J Surg 2002;89:418–422. | | | | Kudo M, et al. Oncology 2007;72(Suppl. 1): 132–138. | | | | | | Koda M, et al. Intern Med 2009;48:11–17. | | | Lo CM, et al. Ann Surg 2007;245: 831–842. | | | |  |
|  | | Kudo M, et al. Oncology 2007;72(Suppl. 1): 132–138. | | | | Lin SM, et al. Cancer 2004;100:376–382. | | | | | | Kubo S, et al. Hepatol Res 2007;37:94–100. | | | Mazzaferro V, et al. Hepatology 2006;44:1543–1554. | | | |  |
|  | | Ikeda K, et al. Hepatology 2000;32:228–232. | | | | Mazzaferro V, et al. Hepatology 2006;44:1543–1554. | | | | | | Kuzuya T, et al. J Gastroenterol Hepatol 2007;22: 1929–1935. | | | Nishiguchi S, et al. Intervirology 2005;48(1):71–75. | | | |  |
|  | | Lin SM, et al. Cancer 2004;100:376–382. | | | | Sakaguchi Y, et al. Intervirology 2005;48:64–70. | | | | | | Lee JW, et al. J Hepatol 2012;56:S398. | | | Shiratori Y, et al. Ann Intern Med 2003;138:299–306. | | | |  |
|  | | Lo CM, et al. Ann Surg 2007;245:831–842. | | | | Shiratori Y, et al. Ann Intern Med 2003;138:299–306. | | | | | | Nishikawa H, et al. Hepatol Res 2014;44:608–620. | | | Sun HC, et al. J Cancer Res Clin Oncol 2006;132:458–465. | | | |  |
|  | | Mazzaferro V, et al. Hepatology 2006;44:1543–1554. | | | | Suou T, et al. Hepatol Res 2001;20:301–311. | | | | | | Su CW, et al. PLoS One 2013;8:e66457. | | |  | | | |  |
|  | | Miyaguchi S, et al. Hepatogastroenterology 2002;49:724–729. | | | |  | | | | | | Wu CY, et al. JAMA 2012;308: 1906–1914. | | |  | | | |  |
|  | | Shiratori Y, et al. Ann Intern Med 2003;138:299–306. | | | |  | | | | | | Yin J, et al. J Clin Oncol 2013;31: 3647–3655. | | |  | | | |  |
|  | | Sun HC, et al. J Cancer Res Clin Oncol 2006;132:458–465. | | | |  | | | | | | Yoshida H, et al. Hepatol Int 2008;2:89–94. | | |  | | | |  |
|  | | Suou T, et al. Hepatol Res 2001;20:301–311. | | | |  | | | | | |  | | |  | | | |  |
| Overlap of included studies among meta-analyses regarding antiviral therapy (continued 3) | | | | | | | | | | | | | | | | | | |  |
| **First author** | | | | **Wong** | | | **Xu** | | | | **Zhang** | | | | | | **Zhang** | |  |
| Journal (Year) | | | | Aliment Pharmacol Ther (2011) | | | Hepatol Res (2014) | | | | Mol Clin Oncol (2014) | | | | | | Int J Cancer (2009) | |  |
| Publication type | | | | Full text | | | Full text | | | | Full text | | | | | | Full text | |  |
| No. Included studies | | | | 9 | | | 9 | | | | 14 | | | | | | 8 | |  |
| No. Included RCTs | | | | 0 | | | 5 | | | | 9 | | | | | | 6 | |  |
| Included studies | | | | Chuma M, et al. J Gastroenterol 2009;44:991–999. | | | Chen LT, et al. Ann Surg 2012;255:8–17. | | | | Chen LT, et al. Ann Surg 2012;255:8–17. | | | | | | Kubo S, et al. Ann Intern Med 2001;134:963–967. | |  |
|  | | | | Hung IF, et al. Am J Gastroenterol 2008;103:1663–1673. | | | Ikeda K, et al. Hepatol Res 2010;40:1168–1175. | | | | Hagihara H, et al. Int J Clin Oncol 2011;16:210–220. | | | | | | Kubo S, et al. Br J Surg 2002;89: 418–422. | |  |
|  | | | | Koda M, et al. Intern Med 2009;48:11–17. | | | Kubo S, et al. Br J Surg 2002;89:418–422. | | | | Ikeda K, et al. Hepatology 2000;32:228–232. | | | | | | Lin SM, et al. Cancer 2004;100:376–382. | |  |
|  | | | | Kubo S, et al. Hepatol Res 2007;37:94–100. | | | Lo CM, et al. Ann Surg 2007;245:831–842. | | | | Jeong S, et al. World J Gastroenterol 2007;13:5188–5195. | | | | | | Lo CM, et al. Ann Surg 2007;245: 831–842. | |  |
|  | | | | Kuzuya T, et al. J Gastroenterol Hepatol 2007;22:1929–1935. | | | Mazzaferro V, et al. Hepatology 2006;44:1543–1554. | | | | Jeong SC, et al. World J Gastroenterol 2007;13:5343–5350. | | | | | | Mazzaferro V, et al. Hepatology 2006;44: 1543–1554. | |  |
|  | | | | Li N, et al. Ann Surg Oncol 2010;17(1):179–185. | | | Oon CJ, et al. Cancer Invest 2003;21: 394–399. | | | | Kubo S, et al. Br J Surg 2002;89:418–422. | | | | | | Nishiguchi S, et al. Intervirology 2005;48(1):71–75. | |  |
|  | | | | Piao CY, et al. Acta Med Okayama 2005;59:217–224. | | | Someya T, et al. J Gastroenterol 2006;41:1206–1213. | | | | Kudo M, et al. Oncology 2007;72(Suppl. 1): 132–138. | | | | | | Shiratori Y, et al. Ann Intern Med 2003;138:299–306. | |  |
|  | | | | Shuqun C, et al. Hepatogastroenterology 2006;53:249–252. | | | Sun HC, et al. J Cancer Res Clin Oncol 2006;132:458–465. | | | | Lin SM, et al. J Hepatol 2007;46:45–52. | | | | | | Sun HC, et al. J Cancer Res Clin Oncol 2006;132:458–465. | |  |
|  | | | | Yoshida H, et al. Hepatol Int 2008;2: 89–94. | | | Uenishi T, et al. Hepatol Res 2006;36:195–200. | | | | Lo CM, et al. Ann Surg 2007;245:831–842. | | | | | |  | |  |
|  | | | |  | | |  | | | | Mazzaferro V, et al. Hepatology 2006;44:1543–1554. | | | | | |  | |  |
|  | | | |  | | |  | | | | Miyaguchi S, et al. Hepatogastroenterology 2002;49:724–729. | | | | | |  | |  |
|  | | | |  | | |  | | | | Shiratori Y, et al. Ann Intern Med 2003;138:299–306. | | | | | |  | |  |
|  | | | |  | | |  | | | | Sun HC, et al. J Cancer Res Clin Oncol 2006;132:458–465. | | | | | |  | |  |
|  | | | |  | | |  | | | | Suou T, et al. Hepatol Res 2001;20:301–311. | | | | | |  | |  |
| Overlap of included studies among meta-analyses regarding antiviral therapy (continued 4) | | | | | | | | | | | | | | | | | | |  |
| **First author** | | | | **Zhou** | | | | | **Zhuang** | | | | | **Zhuang** | | | | |  |
| Journal (Year) | | | | World J Surg (2014) | | | | | PLoS One (2013) | | | | | Zhonghua Gan Zang Bing Za Zhi (2012) | | | | |  |
| Publication type | | | | Full text | | | | | Full text | | | | | Full text | | | | |  |
| No. Included studies | | | | 19 | | | | | 13 | | | | | 8 | | | | |  |
| No. Included RCTs | | | | 1 | | | | | 13 | | | | | 8 | | | | |  |
| Included studies | | | | An HJ, et al. J Gastroenterol Hepatol 2010;25:1876–1882. | | | | | Chen LT, et al. Ann Surg 2012;255:8–17. | | | | | Ikeda K, et al. Hepatology 2000;32:228–232. | | | | |  |
|  | | | | Cai X, et al. J Fujian Med Univ 2008;42:438–440. | | | | | Chung YH, et al. Cancer 2000;88:1986–1991. | | | | | Kubo S, et al. Br J Surg 2002;89:418–422. | | | | |  |
|  | | | | Chan ACY, et al. Arch Surg 2011;146:675–681. | | | | | Ikeda K, et al. Hepatology 2000;32:228–232. | | | | | Li M, et al. J Gastroenterol Hepatol 2009;24:1437–1444. | | | | |  |
|  | | | | Cheng F, et al. Acta Univ Med Nanjing 2010;31:882–888. | | | | | Lai CL, et al. Hepatology 1993;17:389–394. | | | | | Lin SM, et al. Cancer 2004;100:376–382. | | | | |  |
|  | | | | Choi JG, et al. J Clin Gastroenterol 2012;46:413–419. | | | | | Lai CL, et al. Bro Jo Cancer 1989;60:928–933. | | | | | Lo CM, et al. Ann Surg 2007;245:831–842. | | | | |  |
|  | | | | Feng J, et al. China Modern Doctor 2012;50:50–54. | | | | | Li M, et al. J Gastroenterol Hepatol 2009;24:1437–1444. | | | | | Mazzaferro V, et al. Hepatology 2006;44: 1543–1554. | | | | |  |
|  | | | | Hung IF, et al. Am J Gastroenterol 2008;103:1663–1673. | | | | | Lin SM, et al. Cancer 2004;100:376–382. | | | | | Shiratori Y, et al. Ann Intern Med 2003;138:299–306. | | | | |  |
|  | | | | Kim BK, et al. Liver Int 2008;28:393–401. | | | | | Llovet JM, et al. Hepatology 2000;31:54–58. | | | | | Sun HC, et al. J Cancer Res Clin Oncol 2006;132: 458–465. | | | | |  |
|  | | | | Li N, et al. Ann Surg Oncol 2010;17(1):179–185. | | | | | Lo CM, et al. Ann Surg 2007;245:831–842. | | | | |  | | | | |  |
|  | | | | Qu LS, et al. J Gastrointest Surg 2010;14:1111–1120. | | | | | Mazzaferro V, et al. Hepatology 2006;44: 1543–1554. | | | | |  | | | | |  |
|  | | | | Su CW, et al. PLoS One 2013;8:e66457. | | | | | Nishiguchi S, et al. Intervirology 2005;48(1): 71–75. | | | | |  | | | | |  |
|  | | | | Urata Y, et al. J Hepatobiliary Pancreat Sci 2012;19:685–696. | | | | | Shiratori Y, et al. Ann Intern Med 2003;138:299–306. | | | | |  | | | | |  |
|  | | | | Wu CY, et al. JAMA 2012;308:1906–1914. | | | | | Sun HC, et al. J Cancer Res Clin Oncol 2006;132:458–465. | | | | |  | | | | |  |
|  | | | | Wu JC, et al. J Hepatol 2009;51:890–897. | | | | |  | | | | |  | | | | |  |
|  | | | | Yang M, et al. Med J Chin PLA 2010;35:726–732. | | | | |  | | | | |  | | | | |  |
|  | | | | Yang T, et al. Eur J Surg Oncol 2012;38:683–691. | | | | |  | | | | |  | | | | |  |
|  | | | | Yeh CT, et al. Hepatology 2010;52:1922–1933. | | | | |  | | | | |  | | | | |  |
|  | | | | Yin J, et al. J Clin Oncol 2013;31:3647–3655. | | | | |  | | | | |  | | | | |  |
|  | | | | Zhang Z, et al. J Hepatobiliary Surg 2013;21:177–180. | | | | |  | | | | |  | | | | |  |
